# Microbial Communities and Their Predicted Metabolic Functions in Growth Laminae of a Unique Large Conical Mat from Lake Untersee, East Antarctica

**DOI:** 10.3389/fmicb.2017.01347

**Published:** 2017-08-04

**Authors:** Hyunmin Koo, Nazia Mojib, Joseph A. Hakim, Ian Hawes, Yukiko Tanabe, Dale T. Andersen, Asim K. Bej

**Affiliations:** ^1^Department of Biology, University of Alabama at Birmingham, Birmingham AL, United States; ^2^Gateway Antarctica, University of Canterbury Christchurch, New Zealand; ^3^National Institute of Polar Research Tachikawa, Japan; ^4^Carl Sagan Center, SETI Institute, Mountain View CA, United States

**Keywords:** East Antarctica, cyanobacteria, heterotrophic bacteria, mat lamina, QIIME, PICRUSt

## Abstract

In this study, we report the distribution of microbial taxa and their predicted metabolic functions observed in the top (U1), middle (U2), and inner (U3) decadal growth laminae of a unique large conical microbial mat from perennially ice-covered Lake Untersee of East Antarctica, using NextGen sequencing of the 16S rRNA gene and bioinformatics tools. The results showed that the U1 lamina was dominated by cyanobacteria, specifically *Phormidium* sp., *Leptolyngbya* sp., and *Pseudanabaena* sp. The U2 and U3 laminae had high abundances of Actinobacteria, Verrucomicrobia, Proteobacteria, and Bacteroidetes. Closely related taxa within each abundant bacterial taxon found in each lamina were further differentiated at the highest taxonomic resolution using the oligotyping method. PICRUSt analysis, which determines predicted KEGG functional categories from the gene contents and abundances among microbial communities, revealed a high number of sequences belonging to carbon fixation, energy metabolism, cyanophycin, chlorophyll, and photosynthesis proteins in the U1 lamina. The functional predictions of the microbial communities in U2 and U3 represented signal transduction, membrane transport, zinc transport and amino acid-, carbohydrate-, and arsenic- metabolisms. The Nearest Sequenced Taxon Index (NSTI) values processed through PICRUSt were 0.10, 0.13, and 0.11 for U1, U2, and U3 laminae, respectively. These values indicated a close correspondence with the reference microbial genome database, implying high confidence in the predicted metabolic functions of the microbial communities in each lamina. The distribution of microbial taxa observed in each lamina and their predicted metabolic functions provides additional insight into the complex microbial ecosystem at Lake Untersee, and lays the foundation for studies that will enhance our understanding of the mechanisms responsible for the formation of these unique mat structures and their evolutionary significance.

## Introduction

Microbial mats are often considered to be modern day analogs of ancient organo-sedimentary structures such as microbialites and stromatolites, and the geological record indicates their origin as early as ∼3.5 Ga BP (Gerdes, 2010; Seckbach and Oren, 2010). Morphological diversity and longevity of stromatolitic formations from the Archaean and throughout the Proterozoic indicate its success as a growth strategy, and comparisons with extant examples of microbial mats can explain the possible reasons behind this success (Grotzinger and Knoll, 1999; Hofmann et al., 1999). Modern microbial mats often represent metabolically layered structures, capable of oxygenic photosynthesis at the top of a mat where light is most abundant (Foster and Green, 2011). In the deeper segments in the mats, reduced light and depleted oxygen enables the growth of organisms capable of a cascade of anaerobic metabolisms (Kühl and Fenchel, 2000). Such layering is often visible as color changes, with an orange surface from abundant light-protective carotenoid pigments, transitioning to green light-harvesting phycobilin and chlorophyll pigments and pink layers corresponding to photosynthetic sulfur bacteria in anoxic zones. There are many examples of this spatial organization of microbial communities with defined metabolic roles in the literature, and such close proximity of interacting metabolic structures allows for diffusion to be an effective coupling mechanism that provides key advantages to this mat structure (Seckbach and Oren, 2010). Viewed as an integrated unit, the microbial mat shows metabolic coupling, facilitated by this one-dimensional, organized structure, that allows a more efficient utilization of resources than any one organism could do alone (Stahl, 1995; Paerl and Pinckney, 1996; Franks and Stolz, 2009). Indeed, some argue that the organized complexity and physiological cooperativity of a microbial mat is analogous to that of eukaryotic tissues (Webb et al., 2003; Guerrero and Berlanga, 2010).

These laminated microbial mats are often best developed under extreme conditions such as thermal springs, sulfur springs, high salinity and icy polar environments where conditions preclude large metazoan bioturbators that would otherwise disrupt mat formation (Seckbach and Oren, 2010). Microbial mats in ice-covered Antarctic lakes are thought to be particularly well developed as, in addition to the exclusion of higher eukaryotes through extreme conditions and isolation, they are also protected from physical perturbation – wind and wave forcing – by thick, perennial ice cover (Priscu and Foreman, 2009; Cavicchioli, 2015). To date, most of our understanding of microbial mats in the Antarctic continent has come from perennially ice-covered lakes in the McMurdo Dry Valleys (Priscu and Foreman, 2009; Jungblut et al., 2016). In particular, benthic photosynthetic cyanobacterial mats cohabited by heterotrophic bacterial communities have been implicated as the key biological components in maintaining the lake’s productivity and nutrient cycling (Laybourn-Parry and Pearce, 2007; Quesada et al., 2008). However, in addition to the McMurdo Dry Valleys, a wide range of freshwater lakes have been described in the East Antarctic ice plateau, ice-free oases and foothills of the Transantarctic Mountains. Among these, Lake Untersee (71.34°S, 13.45°E) is the largest of the perennially ice-covered lakes in the interior of central Queen Maud Land (Loopmann et al., 1988; Wand et al., 1997, 2006). The lake presents an exclusively microbial ecosystem and consists of unique conical microbial mats that appear as smooth cones, rising to ∼0.5 m above the lake bottom, unlike anything reported thus far from other Antarctic or temperate lakes (Andersen et al., 2011).

Microbial community structures in ice-covered Antarctic lakes have been studied using a range of culture-dependent and culture-independent molecular techniques (Laybourn-Parry and Pearce, 2007; Laybourn-Parry and Wadham, 2014). For instance, microbial community compositions have been investigated in (1) ice core samples from Lake Vida by clone library, and denaturing gradient gel electrophoresis (DGGE) culture-independent approaches targeting the 16S rRNA gene (Mosier et al., 2007); (2) water column samples from Lake Hoare by culturing chemoorganotrophic isolates on media (Clocksin et al., 2007) and molecular probe of SYBR gold (Thurman et al., 2012); (3) water samples from Lake Bonney by restriction fragment length polymorphism (RFLP) analysis (Glatz et al., 2006); and (4) benthic microbial mat samples from Lake Joyce by microscopic analysis (Hawes et al., 2011). Besides microbial mats in marine and other tropical ecosystems (Mobberley et al., 2012; Louyakis et al., 2017), NextGen sequencing technology has been applied to other ecosystems in Antarctica including lakes, sediments, soils, endoliths, and hypoliths. Examples include: (1) benthic mats from Lake Fryxell using Illumina Miseq platform (Jungblut et al., 2016), (2) water samples from Ace Lake using pyrosequencing (Lauro et al., 2011), (3) water samples from Lake Fryxell and Bonney using pyrosequencing (Vick-Majors et al., 2014), (4) soils, endoliths and hypoliths of Victoria Valley in Eastern Antarctica using pyrosequencing (Van Goethem et al., 2016), (5) surface sediments of King George Island and Bransfield Strait of Northwestern Antarctic Peninsula by pyrosequencing (Franco et al., 2017), (6) sediments of freshwater inland lakes and estuarine environment of Byers Peninsula in Antarctica (Gugliandolo et al., 2016), and (7) ice and freshwater water samples from lakes in Schirmacher Oasis of East Antarctica (Mojib et al., 2009).

In this study, we report the taxonomic composition of the microbial community and their predicted metabolic functions of the top (U1), middle (U2), and inner (U3) laminae of a large conical mat from Lake Untersee. We have used NextGen sequencing technology and bioinformatics tools on the community DNA targeting the 16S rRNA gene. The objective of this study was to describe microbial taxa in each of the top three laminae and use a taxonomy-based bioinformatics tool with the appropriate validation method to predict the likely metabolic processes performed by distinct and concentrically distributed microbial communities. We believe the results from this study will help understand the mechanisms for the formation of these unique laminated conical mats in the ecosystem of Lake Untersee.

## Materials and Methods

### Description of Lake Untersee and Sample Collection

Located in the Gruber Mountains, Lake Untersee is 563 m above sea level, 6.5 km long and 2.5 km wide, with an area of 11.4 km^2^ and dammed at its North end by the terminus of the Anuchin Glacier. The lake floor is mostly covered with photosynthetic benthic microbial mats with a conspicuous distribution of vertically rising pigmented large conical mats. Microbial pigments are concentrated near the tops of cones, which appear purple/pink color due to phycoerythrin (Andersen et al., 2011). These mats are soft, composed of organic-rich alternating clear and mineral-rich opaque ∼0.5 mm-thick laminations with a high inheritance of microbes, showing no evidence of lithification (Andersen et al., 2011).

**FIGURE 1:**
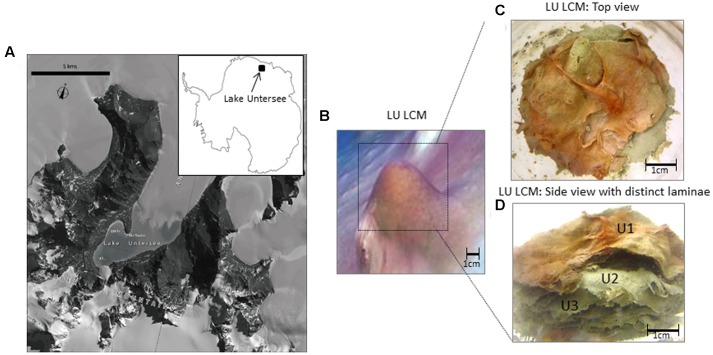
Geographical location, structure and laminae of a large conical mat of Lake Untersee, Antarctica. **(A)** Geographical location and satellite image map of Lake Untersee (71.34° S 13.27° E) Antarctica. (Satellite imagery copyright DigitalGlobe, Inc. Provided by NGA Commercial Imagery Program). **(B)** Underwater photograph of a large conical microbial mat in Lake Untersee. **(C)** Large conical microbial mat in Lake Untersee used in this study showing the top layer with bright-colored predominantly pigmented cyanobacteria. **(D)** Individual lamina of a large conical microbial mat from Lake Untersee used in this study. Notice the top layer consisted of bright colored pigmentation primarily from cyanobacteria. (LU, Lake Untersee; LCM, Large Conical Mat); (U1 = top lamina; U2 = middle lamina, and U3 = inner lamina).

A single core of a conical mat from Lake Untersee (**Figures 1A,B**) was collected by scientific divers utilizing SCUBA by accessing the lake ecosystem through a hole made on the ∼3.5 m surface ice using methods described by Andersen (2007). The core was collected by gently inserting a 50 mm diameter sterile polycarbonate core tube through the top of a conical mat structure, then sealing it with rubber stoppers and returning it to the surface. The core was stored in Antarctic Logistics Centre International (ALCI), Cape Town, South Africa facility first at -20°C in a walk-in freezer and then transported to UAB in dry ice and kept in -20°C freezer until used. The top three laminae (herein U1, top lamina; U2, middle lamina; and U3, inner lamina) were separated from the core and selected individually (**Figures 1C,D**) for sequencing. Each lamina was carefully separated using sterile forceps and rinsed with distilled water to avoid carryover of microorganisms between laminae before DNA extraction.

### Purification of Community DNA

Five samples (∼1 cm^2^ each) from different segments of each lamina of the large conical mat were sliced with a sterile scalpel, transferred into separate microcentrifuge tubes consisting of beads (MO BIO Laboratories Inc., Carlsbad, CA, United States), and homogenized in a high velocity bead beater (BIO101/Savant FastPrep FP120 – Qiagen, Inc., Valencia, CA, United States). DNA was then purified using the PowerBiofilm^®^DNA isolation Kit (MO BIO Laboratories) (Abbasian et al., 2015). The large conical mats used in this study are unique, found only in Lake Untersee. Therefore, to minimize sample collection while obtaining sufficient DNA to investigate the microbial composition of the mat laminae, we used five spatially representative samples from each lamina, and pooled the purified DNA into single samples for amplicon sequencing. The concentration and the quality of the pooled DNA from each mat lamina was determined by using a Lambda II spectrophotometer (Perkin Elmer, Norwalk, Conn.) followed by agarose gel electrophoresis (1% wt/vol agarose in 1X Tris-Acetate-EDTA (TAE) buffer, pH 7.8) to confirm that each sample consists of mostly high molecular weight DNA (>2 kbp) (Ausubel et al., 1987). The DNA was then dried in a Savant Speedvac Evaporator SVC 100H and stored at 4°C until used for sequencing.

### Parallel Bacterial Tag-Encoded FLX-Amplicon Pyrosequencing (bTEFAP)

Bacterial tag-encoded FLX amplicon pyrosequencing (bTEFAP) was performed as described previously using the primers 341F (5′–CCTACGGGAGGCAGCAG–3′) (Muyzer et al., 1993) and 907R (5′–CCGTCAATTCMTTTGAGTTT–3′) (Lane et al., 1985) targeting the V3–V5 region of the 16S rRNA (Dowd et al., 2008a; Suchodolski et al., 2009; Finegold et al., 2010; Smith et al., 2010; Andreotti et al., 2011; Koo et al., 2014a,b). For cyanobacterial diversity, oligonucleotide primers CYA106F (5′- CGGACGGGTGAGTAACGCGTGA-3′) and CYA781R (5′-GACTACWGGGGTATCTAATCCCWTT-3′) (Nübel et al., 1997) targeting the 16S rRNA gene were used. First, a sequencing library was established by using a one-step PCR method with a total of 30 cycles of template DNA amplification with the HotStarTaq Plus Master Mix kit (Qiagen, Inc., Valencia, CA, United States), and amplicons originating and extending from the aforementioned bacteria- and cyanobacteria-specific primers. Then, tag-encoded FLX amplicon pyrosequencing was performed on a Roche 454 FLX instrument with Titanium reagents following the Titanium procedures and RTL protocols at the Research and Testing Laboratory (Lubbock, TX, United States)^1^ (Dowd et al., 2008b). The raw sequence reads have been deposited on the publicly available database located at the SRA of NCBI under accession number SRP078055.

### Sequence Processing Using Bioinformatics Tools

All total of 15,999 raw barcoded pyrosequence reads from the bacterial and cyanobacterial 16S rRNA genes from U1, U2, and U3 laminae were merged into fna, qual, and mapping file formats (**Table 1**). Then, the *split_libraries.py* command was used to split the sequence libraries based upon the barcodes specified in the mapping file by using the Quantitative Insights into Microbial Ecology (QIIME, v1.8.0) pipeline (Caporaso et al., 2010b). Chimera sequence reads were removed using *identify_chimeric_seqs.py* module of USEARCH (Edgar, 2010). The poor-quality sequence reads that did not match sufficiently with the primer sequences, barcode sequences, or contained any ambiguous reads, or homopolymers of more than 8 bp, or had average quality scores of less than 28 were excluded from the rest of the analysis (Kunin et al., 2010) resulting into a total of 14,137 sequence reads (**Table 1**). The quality of sequence reads in all samples were evaluated by PRINSEQ (Schmieder and Edwards, 2011). The result showed a normally distributed sequence length (mean sequence length value of 380.78 ± 72.60 bp) and GC content (mean value of 54.55 ± 2.24%). The majority of the sequences had a mean Phred quality score of 32–33, and no occurrence of ambiguous nucleotides (N) (Schmieder and Edwards, 2011). After the trimming processes, reads were clustered into a total of 1,630 unique Operational Taxonomic Units (OTUs) at a 3% distance using UCLUST (**Table 1**) (Edgar, 2010). Then, representative sequences were selected from each OTU and aligned using PyNAST (Caporaso et al., 2010a). The Ribosomal Database Program (RDP) classifier^2^, trained using the Greengenes (v13.8) database^3^ (McDonald et al., 2012), was used to assign taxonomy for each representative sequence at a confidence threshold of 80% (0.8) (Wang et al., 2007). A single OTU table consisting of all OTUs, taxonomic and abundance information was used to generate stacked column bar graphs of relative abundance using Microsoft Excel software (Microsoft, Seattle, WA, United States). In order to normalize sequence reads across all samples, the “single_rarefaction.py” command in QIIME was used, which resulted in an even sampling depth of 4,460 reads per sample. The subsampled sequence reads were then used for downstream Alpha [observed OTUs, Shannon diversity index (Shannon et al., 1964), and Simpson diversity index (Simpson, 1949)], as well as Beta (principle coordinate analysis (PCoA) plot) diversity analyses. The real_edge table and real_node_table files generated from QIIME (v1.8.0) were exported into Cytoscape (v2.8.2) (Shannon et al., 2003) and an edge-weighted force-directed eweight layout was selected to generate OTU network (Pope et al., 2012). Top 25 most highly abundant taxa at the family level were selected, and then used to generate the heatmap using “*heatmap.2*” function in R package^4^.

**Table 1:** Raw and trimmed sequence reads following tag-encoded FLX amplicon pyrosequencing.

	Top layer (U1)	Middle layer (U2)		Inner layer (U3)		Total (U1+U2+U3)
				
Primer set	Cyanobacteria-specific	Bacteria	Cyanobacteria-specific	Bacteria	Cyanobacteria-specific	
Number of raw sequences	5,003	1,714	3,960	1,488	3,834	15,999
Number of trimmed sequences	4,766	4,904		4,467		14,137
Number of OTUs	237	665		728		1,630
Shannon diversity	3.625	6.615		6.830		
Simpson diversity	0.693	0.960		0.961		


*The number of OTUs and calculated alpha diversity indices, Shannon diversity and Simpson diversity, of U1, U2, and U3 laminae of large conical mat samples from Lake Untersee are listed. Normalized sequence reads were used to measure Shannon and Simpson diversity indices.*

### Oligotyping Analysis

Oligotyping (v1.0) bioinformatics tools was used to assign an oligotype identity from similarly clustered sequence reads based on their nucleotide variation (Eren et al., 2013, 2014; Schmidt et al., 2014). In this study, family Cytophagaceae, Opitutaceae, and Xanthomonadaceae, and genus *Phormidium* and *Leptolyngbya* sequence reads were extracted separately from the “*seq.fna*” file generated through the QIIME pipeline (v1.8.0), and the five extracted sequence reads were used separately to calculate Shannon entropy (Shannon et al., 1964) using “*entropy-analysis*” command embedded in oligotyping (v1.0). Since the number of sequences from the five extracted sequence files were below 5,000 per reads, a value of the minimum count of the most abundant unique sequence in an oligotype (M) was set up as 5 for Opitutaceae, 3 for *Phormidium*, 2 for Cytophagaceae and *Leptolyngbya*, 1 for Xanthomonadaceae, and a value of the minimum actual abundance of an oligotype across all layer samples (A) was set up as 10 for Opitutaceae or 0 for Cytophagaceae, Xanthomonadaceae, *Phormidium*, and *Leptolyngbya* to conduct oligotyping (v1.0) (Menke et al., 2014). Oligotypes that did not meet these criteria were removed, and ≥80% reads were retained for each oligotyping results. After completing the analysis, each oligotype representative sequence was subjected to BLAST search in NCBI non-redundant (nr) database^5^.

### PICRUSt Analysis for Predicted Metabolic Functions

Phylogenetic Investigation of Communities by Reconstruction of Unobserved States (PICRUSt v1.0.0^6^) (Langille et al., 2013) analysis was conducted to determine the predicted metabolic functions of the microbial communities in three large conical mat laminae. Cyanobacteria and heterotrophic bacterial sequences were separated from the *seq.fna* file and each was used for the PICRUSt analysis. As suggested in the PICRUSt (v1.0.0) protocol, OTUs were closed-referenced picked against the Greengenes (v13.5) database at a 97% identity from each sequence file. The resultant OTU table was then used to predict metabolic functions by referencing the Kyoto Encyclopedia of Genes and Genome (KEGG) Orthology (KO) Database^7^ (Kanehisa and Goto, 2000; Kanehisa et al., 2014), using the “*predict_metagenomes.py*” command in PICRUSt (v1.0.0). The likely accuracy of the PICRUSt predictions was estimated using the Nearest Sequenced Taxon Index (NSTI) value, which is the average branch length separating OTUs in each sample from the reference genome (Langille et al., 2013). The NSTI value is considered to be the standard method for validation of the PICRUSt-predicted KEGG functional groups (Langille et al., 2013; Staley et al., 2014; Zeng et al., 2015; Hu et al., 2016; Lokesh and Kiron, 2016; Lopes et al., 2016; Yuan et al., 2016). Low NSTI values imply a close relationship to organisms in the known microbial reference genome databases, representing high accuracy of the predicted KEGG functional groups. A pairwise statistical comparison of the predicted metabolic functions in the three laminae (U1 vs. U2; U1 vs. U3; and U2 vs. U3) was carried out using STAMP (Parks et al., 2014), two sided *G*-test (w/Yates’) + Fisher’s statistical test with the DP: asymptotic-CC confidence interval method with the Benjamini–Hochberg FDR multiple test correction using a *P*-value of <0.05 (Mason et al., 2012; Li et al., 2014).

## Results

### Taxonomic Distribution in Top Three Laminae

**FIGURE 2:**
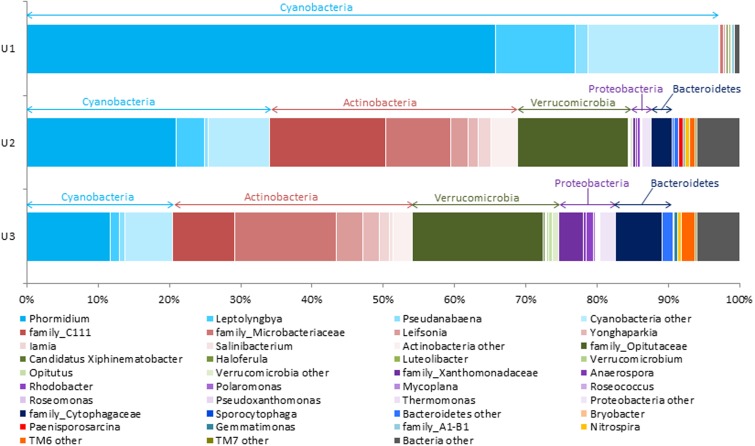
Stacked column bar graph representing the relative abundance and distribution of the most abundant taxa up to genus level across the three laminae in this study. The figure shows that while phylum Cyanobacteria was mostly dominant in U1, the relative abundance of Cyanobacteria decreased through U2 and U3. At the highest resolution, *Phormidium* was found to be the most abundant taxa within the Cyanobacteria in the all laminae. The U3 had a higher abundance of Microbacteriaceae, Opitutaceae, Xanthomonadaceae, and Cytophagaceae as compared to U2, except for C111, the abundance of which was found to be decreased in U3. Sequence data analyses was performed using QIIME (v1.8.0), and the stacked column bar graph was generated using Microsoft Excel software (Microsoft, Seattle, WA, United States).

The relative abundances of microbial taxa identified to the most resolvable level (up to family or genus) in each of the three laminae are elaborated in **Figure 2**. The U1 lamina represented by the highest relative abundances of members from phylum cyanobacteria (91.2%). Within cyanobacteria, *Phormidium* (65.8%) was found to be a dominant taxon followed by *Leptolyngbya* (11.1%), and *Pseudanabaena* (1.8%) (**Figure 2**). Pyrosequencing using the bacteria-specific primers on the U1 lamina produced a negligible number of poor quality sequence reads (NCBI accession number: SRP104174). Additionally, the cyanobacteria-specific primers used on the same U1 sample showed a low occurrence of sequences belonging to heterotrophs (<1%).

The U2 and U3 laminae were dominated by heterotrophic bacterial taxa. Within the heterotrophs, family C111 and Microbacteriaceae, Opitutaceae, Cytophagaceae, and Xanthomonadaceae were found to be the highly abundant taxa in U2 and U3 laminae (**Figure 2**). At a higher taxa resolution (up to genus level) from within these families, *Leifsonia*, *Iamia*, *Rhodobacter*, and *Opitutus* were detected in both U2 and U3 laminae (**Figure 2**).

Further, Actinobacteria (34.4%) and Verrucomicrobia (16%) were found to be the dominant heterotrophic bacterial taxa, and cyanobacteria were represented by relatively low abundances of *Phormidium* (20.6%), *Leptolyngbya* (4%), and *Pseudanabaena*: (0.5%) in the U2 lamina (**Figure 2**). The taxonomic distribution of heterotrophic microbial communities in the U3 lamina revealed high relative abundances of Actinobacteria (33.3%) and Verrucomicrobia (20.4%), and low relative abundances of cyanobacteria representing *Phormidium* (11.5%), *Leptolyngbya* (1.3%), and *Pseudanabaena* (0.8%) (**Figure 2**). Interestingly, *Polaromonas*, *Roseomonas*, and *Thermomonas* were only detected in the U3 lamina (**Figure 2**). A detailed taxonomic list at the genus level for each of the three laminae has been included in the Supplementary Table 1. The overall phylum and genus level taxonomic distribution of cyanobacteria and heterotrophic bacteria and their relative abundances in U1, U2, and U3 laminae have been elaborated in Supplementary Figures 1A–H, respectively.

### Differentiation of Taxa Using Oligotyping Method

**FIGURE 3:**
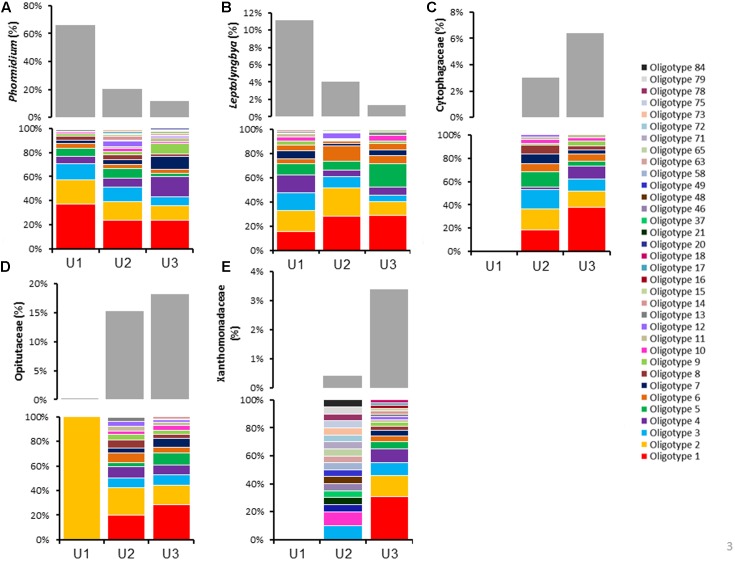
Oligotype profiles describing the distribution and abundances of each dominant microbial taxon in three laminae (U1, U2, and U3). Color bars within each (but not between) graph represent the oligotypes within **(A)** *Phormidium*, **(B)** *Leptolyngbya*, **(C)** Cytophagaceae, **(D)** Opitutaceae, and **(E)** Xanthomonadaceae. The gray bars above each color graph represent the abundance of sequence reads that were assigned to different bacterial taxa. Oligotyping analyses were performed using the open-source pipeline for oligotyping, available at http://oligotyping.org. Note that no Cytophagaceae and Xanthomonadaceae sequences were identified in U1 lamina, as shown in graphs **(C,E)**.

To improve the resolution of the taxa up to species level, sequences extracted from *Phormidium*, *Leptolyngbya*, Cytophagaceae, Opitutaceae, and Xanthomonadaceae from the three laminae were subjected to oligotyping followed by BLAST analyses (**Figure 3** and Supplementary Table 2). Total sequences, the number of oligotypes, and the number of shared oligotype for these taxa were elaborated in **Table 2**. Family C111 and Microbacteriaceae were excluded from oligotyping analysis due to the high variation among each of the representative sequences, as well as limited genera information available for these families within 16S rRNA reference taxonomic databases (e.g., Greengenes). The representative oligotypes, their diversity and relative abundances in U1, U2, and U3 laminae are listed in **Figure 3**, and in Supplementary Table 2. BLAST results of the representative oligotypes from the *Phormidium* taxon showed that *Phormidium* cf. *uncinatum* CYN108 and *Phormidium* sp. Ant-Orange were found to be the most highly abundant taxa in three laminae (**Figure 3A** and Supplementary Table 2). *Phormidium autumnale*, *Phormidium* cf. *uncinatum* CAWBG523, and *Phormidium* spp. were also found in each of the three laminae (**Figure 3A** and Supplementary Table 2). *Leptolyngbya antarctica* ANT and *Leptolyngbya* sp. were found to be the most abundant taxa in U1, U2, and U3 laminae (**Figure 3B** and Supplementary Table 2). Uncultured Bacteroidetes, *Cytophaga* sp., and *Flexibacter aggregans* were found to be highly abundant in U2 and U3 laminae (**Figure 3C** and Supplementary Table 2). There were no sequences assigned to Cytophagaceae in U1 lamina (**Figure 3C**). All oligotypes identified within Opitutaceae in U1, U2, and U3 laminae closely matched with genus *Opitutus* spp. (**Figure 3D** and Supplementary Table 2). In addition, *Pseudoxanthomonas* sp., *Pseudomonas* sp., *Xanthomonas* sp., and *Stenotrophomonas* sp. belonging to Family Xanthomonadaceae were highly dominant in U2 and U3 laminae (**Figure 3E** and Supplementary Table 2). The identity and the e-value of each taxa resulting from the BLAST analysis have been listed in Supplementary Table 2.

**Table 2:** Oligotyping results for the U1, U2, and U3 laminae of a large conical mat samples from Lake Untersee used in this study.

Taxon	Total sequence reads used for Oligotyping in U1/U2/U3	Number of Oligotype in U1	Number of Oligotype in U2	Number of Oligotype in U3	Number of shared Oligotype in all samples^∗^
**Genus**					
*Phormidium*	2,668/864/431	14	20	20	14
*Leptolyngbya*	461/178/51	15	15	12	12
**Family**					
Cytophagaceae	0/132/234	0	12	11	0
Opitutaceae	1/683/737	1	14	14	1
Xanthomonadaceae	0/20/150	0	18	67	0


*For each taxon, the total sequence reads used for oligotyping, number of oligotype in each lamina, and number of shared oligotype in all laminae are listed. ^∗^All samples represent the presence of oligotype in U1, U2, and U3.*

### Alpha and Beta Diversity Estimation of the Microbial Communities

**FIGURE 4:**
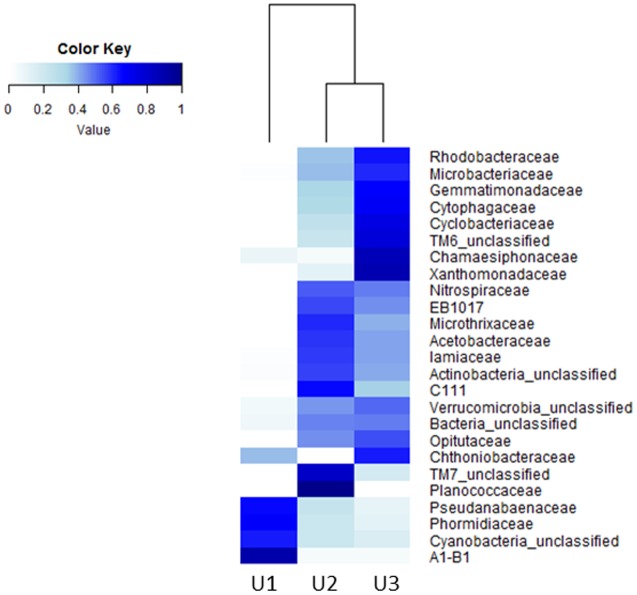
Heatmap generated by using the “heatmap.2” function in R package to show the microbial compositions of the top 25 taxa at family level in U1, U2, and U3 of a conical microbial mat from Lake Untersee, Antarctica. The rows indicate the bacterial taxa and the columns represent the three mat laminae used in this study.

A heatmap was constructed to represent the microbial abundances and compositions of top 25 taxa at family level in U1, U2, and U3 laminae (**Figure 4**). The relative abundance for each bacterial taxon was shown by color intensity, as depicted by the legend elaborated in **Figure 4**.

**FIGURE 5:**
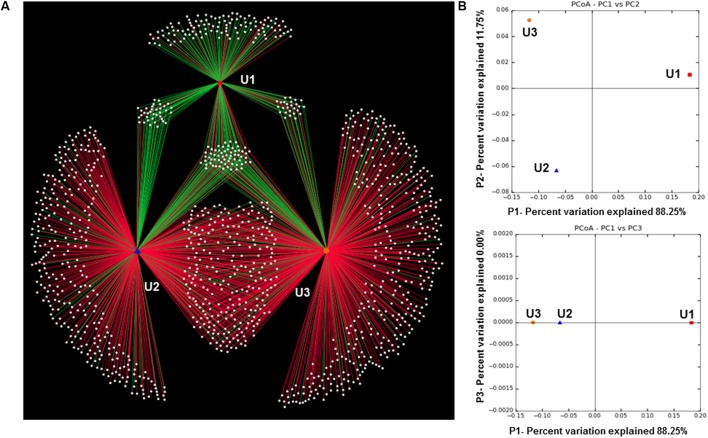
**(A)** Operational Taxonomic Units (OTU) network generated through QIIME (v1.8.0) and Cytoscape (v2.8.2). The green and red edges from the peripheral white nodes are OTUs representing cyanobacteria and heterotrophic bacteria, respectively. The Edge-Weighted Spring-Embedded Eweights format was used to generate the OTU network map; **(B)** two PCoA plots generated by QIIME (v1.8.0) representing significant differences in bacterial community cluster among U1, U2, and U3 of the conical microbial mat from Lake Untersee, Antarctica.

The Shannon diversity index (Shannon et al., 1964) and Simpson diversity index (Simpson, 1949) showed microbial diversity in each of the three laminae (**Table 1**). The weighted UniFrac generated OTU network (**Figure 5A**) and PCoA plots (**Figure 5B**) show distribution of the observed microbial taxa in U1, U2, and U3 laminae. The results showed that unique cyanobacterial OTUs clustered in the U1 lamina and a large number of common heterotrophic bacterial OTUs in U2 and U3 laminae (**Figure 5A**), which is also supported by the PCoA plot (**Figure 5B**). The rarefaction curves of sequences from all three laminae were saturated with respect to the species richness and diversity (Supplementary Figure 2).

### Predicted Metabolic Functions Using PICRUSt

**FIGURE 6:**
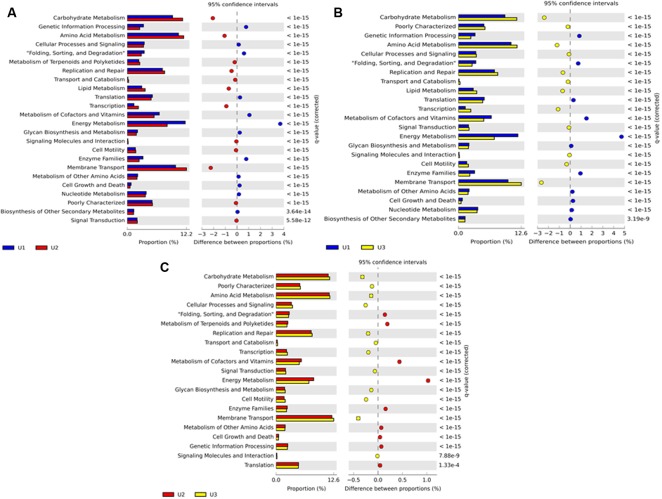
Stacked column bar graph representing the predicted metabolic attributes compare between **(A)** U1 and U2; **(B)** U1 and U3; and **(C)** U2 and U3 laminae of a large conical mat from Lake Untersee, Antarctica. All sequence reads were used to predict functions against the KEGG database (http://www.genome.jp/kegg/), which is implemented in PICRUSt (http://picrust.github.io/picrust/) bioinformatics software package. The mean Nearest Sequenced Taxon Index (NSTI) value for all three laminae was 0.11 ± 0.017 s.d.

Predicted metabolic functions of the microbial communities in U1, U2, and U3 laminae were investigated by using PICRUSt (v 1.0.0) (**Figure 6**). The KEGG level 2 results showed that the U1 lamina had a high number of sequences assigned to energy metabolism such as carbon fixation in photosynthetic organisms; oxidative phosphorylation; photosynthesis; and methane, nitrogen, and sulfur metabolism. The U2 and U3 laminae depicted functional categories such as amino acid and carbohydrate metabolisms, and membrane transport proteins (**Figure 6** and Supplementary Table 3). Results using PICRUSt on cyanobacteria collectively showed functional categories of transporters, photosynthesis proteins, ABC transporters, purine metabolism, peptidases, DNA repair and recombination proteins, photosynthesis, porphyrin and chlorophyll metabolisms, and photosynthesis-antenna proteins at KEGG level 3 (Supplementary Figure 3 and Table 4). The functional categories collectively for the heterotrophic bacteria at KEGG level 3 showed a high number of sequences in transporters, ABC transporters, DNA repair and recombination proteins, purine metabolism, phosphotransferase system (PTS), and a high number of sequences for ribosome and two-component systems (Supplementary Figure 3 and Table 4). Detailed PICRUSt results of additional metabolic functions at KEGG levels 2 and 3 have been elaborated in Supplementary Figure 3 and Tables 3, 4.

**FIGURE 7:**
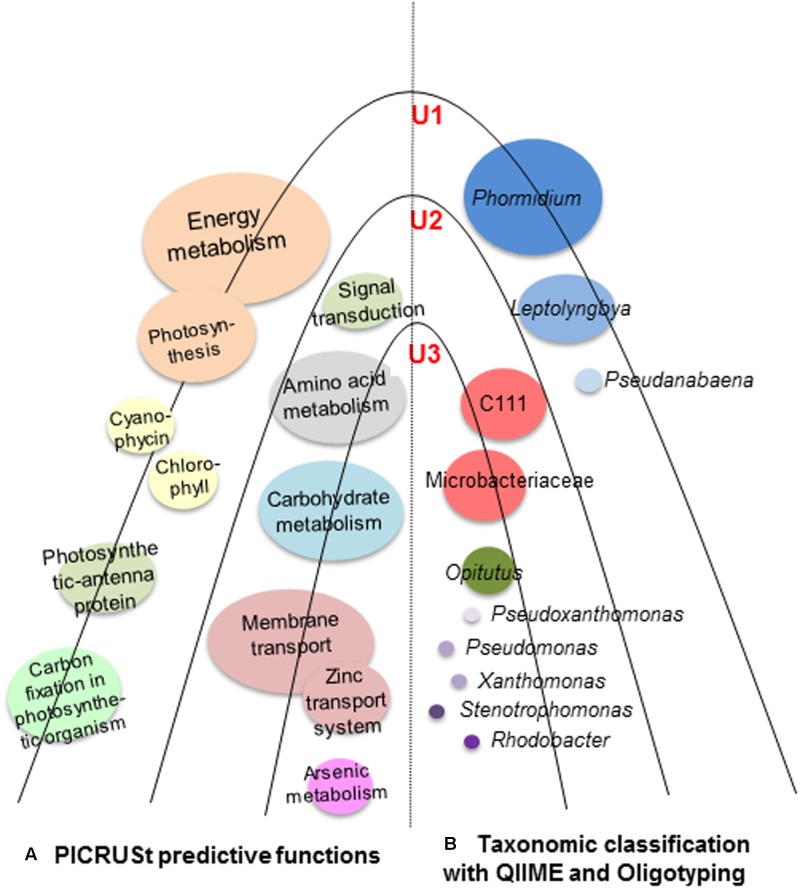
Schematic diagram of U1, U2 and U3 laminae of a large conical mat from Lake Untersee, Antarctica. This diagram shows **(A)** predicted metabolic functions (PICRUSt) elucidating the metabolism of the carbon, sulfur, and nitrogen in the conical mat; and **(B)** microbial taxonomic classification of the microbial communities in the conical mat was analyzed by both QIIME and Oligotyping. The U1 lamina was dominated by cyanobacteria, which contributed to high energy metabolism. The U2 and U3 laminae were dominated by heterotrophic bacteria, which were involved in various metabolic activities, particularly membrane transport, amino acid and carbohydrate metabolism. The mean Nearest Sequenced Taxon Index (NSTI) value for all three laminae was 0.11 ± 0.017 s.d.

The NSTI value was determined for each lamina (0.10 in U1; 0.13 in U2; and 0.11 in U3) and the mean value calculated to be 0.11 ± 0.017 s.d. for all laminae. These values are either comparable or better than the mean NSTI value ranging from 0.10 ± 0.016 s.d. to 0.23 ± 0.02 s.d. reported for microbial communities of samples collected from diverse environments (Langille et al., 2013; Staley et al., 2014; Zeng et al., 2015; Hu et al., 2016; Lokesh and Kiron, 2016; Lopes et al., 2016; Yuan et al., 2016). These values indicate that the predicted metabolic functions displayed by the observed microbial taxa in each of the three laminae are close to the known microbial reference genome databases, and thus imply a higher accuracy of the predictions. Predictive microbial functions along with the microbial taxonomic classification of the microbial communities of U1, U2, and U3 laminae of the conical mat have been summarized in **Figure 7**.

## Discussion

The mats in Antarctic ice-covered lakes are comprised of complex microbial communities and are composed primarily of cyanobacteria and heterotrophs (Andersen et al., 2011; Mackey et al., 2015; Jungblut et al., 2016). The large conical mats of Lake Untersee form distinct laminae with each layer being ∼0.5 mm in thickness, and a top lamina dominated by distinctive pigmented photosynthetic cyanobacteria (Andersen et al., 2011). Since these laminae are easily distinguishable, we have explored the taxonomic composition and predicted metabolic functions of microbial communities of the top three laminae (U1, U2, and U3) by using NextGen sequencing technology and bioinformatics tools. The results showed that cyanobacteria dominated the U1 lamina (**Figures 2**, **4**, **5**). More specifically, *Phormidium* sp. was the most prevalent cyanobacteria, followed by *Leptolyngbya* sp. and *Pseudanabaena* sp. in these laminae (**Figures 2**, **3**). These cyanobacterial taxa have previously been reported to occur in other benthic microbial mats, including the low irradiance environments of perennially ice-covered Antarctic lakes (Oguni and Takahashi, 1989; Taton et al., 2003; Jungblut et al., 2010; Andersen et al., 2011; Vincent and Quesada, 2012; Zhang et al., 2015). Hawes et al. (2013) demonstrated the composition of cyanobacterial communities of the microbial mats in areas recently flooded due to rising lake levels at Lake Vanda in the McMurdo Dry Valleys. They showed that the upper 2–4 laminae were dominated by orange-brown pigmented myxoxanthophyll intertwined with *Leptolyngbya* trichomes. Up to six laminae below the top layer were inhabited by phycobilin-rich green/pink-pigmented *Leptolyngbya*, *Oscillatoria* and *Phormidium* morphotypes. In a separate study, Sutherland and Hawes (2009) explored whether the changes in the cyanobacterial community composition in annual growth layers could potentially indicate proxies of past growth performances of microbial mats in various depths of Lake Hoare of the McMurdo Dry Valleys. The results from this study showed that there was no apparent effect of the species composition of cyanobacterial communities along with organic matter and extracellular polysaccharides in mats from all depths. Hawes et al. (2016) described the development of microbial mats in response to variations in irradiance in Lake Hoare of McMurdo Dry Valleys. This study revealed that photosynthetic biomass and species composition in mats remain remarkably stable for long period. The accrual of laminae and their thicknesses were directly correlated with the intensity of irradiance passing through the ice cover of this lake. The results from these studies corroborated our observation of the overall distribution of high abundances of *Leptolyngbya* and *Phormidium* in the U1 lamina of Lake Untersee large conical mats.

Further oligotyping analysis within *Phormidium* sp. showed three dominant species, *P. autumnale, P.* cf. *uncinatum* and *P. Ant-Orange* in U1, U2, and U3 laminae (**Figure 3A** and Supplementary Table 2). Particularly, the heightened observance of *P. autumnale* in the U1 lamina in this study corroborated previously reported microscopic and other biochemical analyses methods (Andersen et al., 2011). *P. autumnale* is a gliding photosynthetic cyanobacterium and is known to manifest horizontal to upward gliding movement along mucous-like extracellular polymeric substances (EPS), which is driven by changes in the microscale gradient of nutrients in their surrounding environment (Geurts, 1976; Freytet and Plet, 1996). Thus, the relatively high abundance of *Phormidium*, particularly *P. autumnale* in our study indicates perhaps their crucial role in the formation of the vertically oriented, uniquely structured large conical mats in Lake Untersee. However, such functions need to be verified by future experiments, to demonstrate gliding behavior, growth rate and gene regulation (Hawes et al., 2013).

Oligotyping analysis within *Leptolyngbya* sp. revealed two abundant species, *L. antarctica* ANT and *L*. sp. CENA538 (**Figure 3B** and Supplementary Table 2). Specifically, the dominant presence of *L. antarctica* ANT across all three laminae supports previous reports of this cyanobacterium occurring in the majority of benthic microbial communities of Antarctic lakes (Komárek, 2007; Zhang et al., 2015; Jungblut et al., 2016). The straight and irregularly coiled *L. antarctica* has been reported to form massively large mats particularly in the benthic environments of continually frozen lakes (Komárek, 2007). Their dominance in Lake Untersee’s limited photosynthetically active radiation (PAR) and UV-B environment could be attributed to the previously reported study where exposing *Leptolyngbya-*dominated microbial mats to increased UV-B relative PAR (400–700 nm) resulted in both significantly lower chlorophyll a concentrations and higher photochemical inhibition in the mats (George et al., 2001).

Lastly, the oligotyping analysis of the third most abundant cyanobacteria in our study, *Pseudanabaena* sp., revealed two species: *Pseudanabaena catenata* UAM 412, and *Pseudanabaena minima* CHAB705. Our observation corroborated previously reported studies of their widespread distribution in Antarctic aquatic ecosystems, but to a lesser abundance as compared to *Phormidium* and *Leptolyngbya* (Taton et al., 2006; Hawes et al., 2013).

Given the prominent abundance of the three aforementioned cyanobacteria in the U1 lamina, one can associate the notable observances of PICRUSt-predicted functional categories to these taxa as photosynthetic organisms, namely energy metabolism, cyanophycin, chlorophyll, photosynthesis protein, photosynthetic-antenna protein, and carbon fixation (**Figures 6**, **7**, Supplementary Figure 3, and Tables 3, 4). Furthermore, our PICRUSt results support previous reports that have demonstrated that cyanobacteria are involved in photosynthetic activities (Couradeau et al., 2012) and cyanophycin production, as mat-builders in all microbialites (White et al., 2015).

The taxonomic compositions in the U2 and U3 laminae revealed a diverse group of heterotrophic bacteria with a high abundance of Actinobacteria, Verrucomicrobia, Proteobacteria, and Bacteroidetes (**Figures 2**, **4**, **5**), which were comparable to the microbial mat communities in marine and freshwater ecosystems such as Shark Bay in Western Australia (Allen et al., 2009; Goh et al., 2009); Highbourne Cay in Bahamas (Reid et al., 2000; Myshrall et al., 2010; Mobberley et al., 2013); Pavilion Lake (Laval et al., 2000; White, 2014); Clinton Creek in Canada (Power et al., 2011; White, 2014); and Highborne Cay in Northern Exumas, Bahamas (Mobberley et al., 2012; Louyakis et al., 2017). As reported in other studies, the heightened abundance of Actinobacteria in Lake Untersee microbial mats could imply a possible role in recycling organic components from decomposed cyanobacteria from the top (U1) to the inner laminae (U2 and then U3) (Goodfellow and Williams, 1983; Glöckner et al., 2000; Rondon et al., 2000; Zwart et al., 2002; Cottrell et al., 2005; Warnecke et al., 2005; Ward and Bora, 2006; Ventura et al., 2007; Philosof et al., 2009). The sequence data of U1 lamina using the bacteria-specific primers revealed a negligible abundance of heterotrophs. Subsequent bioinformatics analyses were unable to process the sequence reads. The raw sequences reads for the U1 lamina representing bacterial taxa are available at NCBI under accession number SRP104174.

The oligotyping analysis within Verrucomicrobia showed the diverse psychrotolerant, obligatory alkaliphilic, polysaccharide-utilizing ultramicrobacterium *Opitutus* spp., including *Opitutus terrae*, of family Opitutaceae (**Figure 3D** and Supplementary Table 2) (Wilhelm et al., 2011; Larose et al., 2013). Considering their preference to anaerobic habitats, and anaerobic nitrogen fixation metabolism reported in previous studies (Wilhelm et al., 2011; Larose et al., 2013), the observation of Opitutaceae in the inner laminae led us to predict an oxygen-depleted microenvironment within the large conical mats of Lake Untersee. However, the existence of such a microenvironment and the nitrogen fixation properties of *Opitutus terrae* in the inner laminae requires further investigation.

Previous reports have suggested the ability of *Cytophaga* to enzymatically lyse cyanobacteria and Gram-positive bacteria, in addition to performing photooxidation through respiration and promoting nitrogen fixation by lowering the oxygen concentrations in the surrounding environment (Marshall, 1989; Madigan et al., 2008). Thus, the existence of Cytophagaceae in Lake Untersee mats in U2 and U3 laminae could demonstrate a possible mutually beneficial relationship with cyanobacteria, but such function needs to be verified by future studies.

PICRUSt analysis of the observed heterotrophic bacterial taxa showed increased predicted metabolic functions in U2 and U3 lamina, which have previously been reported to be common properties of heterotrophic bacterial communities (**Figures 6**, **7**, Supplementary Figure 1, and Tables 3, 4) (Newton et al., 2011; Varin et al., 2012). Previous reports showed that Actinobacteria and Proteobacteria are especially capable of conducting carbon metabolism, membrane transport system including zinc transport, and the stress response regulatory system (Vikram et al., 2016), which supports our PICRUSt results. Interestingly, PICRUSt predicted an elevated activity of arsenic metabolism in the U3 lamina, a mechanism that has been previously suggested to be a prominent characteristic in ancient microbial mats that are over 2.7 billion years old (Sforna et al., 2014; White et al., 2015). The mean NSTI values of our samples using PICRUSt was 0.11 ± 0.017 s.d., which is similar to or better than previously reported studies on the microbiota of: seepweed *Suaeda salsa* (Amaranthaceae) (NSTI = 0.17 ± 0.02 s.d.), Atlantic salmon skin (NSTI range from 0.10 ± 0.016 s.d. to 0.17 ± 0.065 s.d.), rabbit fecal samples (NSTI = 0.195 ± 0.05 s.d.), gut microbiota of miniature piglets (NSTI = 0.1469 ± 0.01902 s.d.); and environmental samples such as: hypersaline mat microbiome (mean NSTI = 0.23 ± 0.07 s.d.), soil samples from cold deserts of the Antarctic McMurdo Dry Valleys and hot deserts of the Southwestern United States (mid-range NSTI = 0.17 ± 0.02 s.d.), and rhizosphere microbial communities (mean NSTI = 0.23 ± 0.02 s.d.). Based on these NSTI values we infer with high confidence that our predicted metabolic functions are robustly supported by the reference microbial genome database (Fierer et al., 2012; Langille et al., 2013; Staley et al., 2014; Zeng et al., 2015; Hu et al., 2016; Lokesh and Kiron, 2016; Lopes et al., 2016; Yuan et al., 2016).

In summary, the large conical mats of Lake Untersee have thus far been considered unique amongst all reported perennially ice-covered lakes in the Antarctic continent (Andersen et al., 2011, 2015). These mats offer distinct laminae harboring diverse taxonomic compositions. The distribution of microbial communities showed high abundance of photosynthetic cyanobacteria in the outer U1 lamina. A high abundance of *P. autumnale* with gliding properties along the EPS-enriched U1 lamina indicate this species as a likely contributor for the vertical structure of these large conical mats. Predicted metabolic functions of the phototrophic cyanobacterial community in the U1 lamina showed this community capable of producing nutrients by photosynthesis. The U2 and U3 laminae consisted of a high abundance of heterotrophic bacterial taxa, of which their predicted metabolic functions determined by PICRUSt are likely involved in the decomposition of organics and metabolites. Further shotgun metagenomics analysis would substantiate detailed metabolic traits in each of these microbial communities, which could serve as an ideal model system to better understand the microbial processes that possibly contribute to the formation of these structures analogous to the ancient stromatolites.

## Author Contributions

HK conducted the bioinformatics analysis of the NGS data. DA, YT, and IH conducted field work in Antarctica including the collection and processing of a conical stromatolite mat sample from Lake Untersee. NM conducted DNA extraction and pursued the DNA samples to Research and Testing Laboratory (Lubbock, TX, United States) for Pyrosequencing. AB and DA conceived the study. HK, JH, and AB wrote the manuscript and all authors contributed and commented wherever appropriate in the manuscript. AB oversaw the overall progress of this study.

## Conflict of Interest Statement

The authors declare that the research was conducted in the absence of any commercial or financial relationships that could be construed as a potential conflict of interest.

## Footnotes

1 www.researchandtesting.com

2 http://rdp.cme.msu.edu/

3 http://greengenes.secondgenome.com/

4 http://CRAN.R-project.org/package=gplots

5 http://blast.ncbi.nlm.nih.gov/Blast.cgi

6 http://picrust.github.io/picrust/

7 http://www.genome.jp/kegg/ko.html
