# Supplementary material for: Microbial Communities and Their Predicted Metabolic Functions in Growth Laminae of a Unique Large Conical Mat from Lake Untersee, East Antarctica
Source: Front Microbiol. 2017 Aug 4;8:1347. doi: 10.3389/fmicb.2017.01347 (PMC5543034; doi:10.3389/fmicb.2017.01347)
Supplement: Supplementary file 1 [file Table_1.pdf]

Supplementary Table 1: Elaborated relative abundances of the all taxa found in all three laminae (U1, U2, and U3) of the conical microbial mat from Lake Untersee, Antarctica

**1. Phylum level**			
Taxa	U1 lamina	U2 lamina	U3 lamina
k__Bacteria;Other	0.80%	5.90%	6.00%
k__Bacteria;p__Acidobacteria	0.00%	0.10%	0.10%
k__Bacteria;p__Actinobacteria	0.90%	34.40%	33.30%
k__Bacteria;p__Armatimonadetes	0.00%	0.00%	0.00%
k__Bacteria;p__Bacteroidetes	0.00%	4.00%	8.10%
k__Bacteria;p__Chlorobi	0.00%	0.00%	0.00%
k__Bacteria;p__Chloroflexi	0.00%	0.00%	0.00%
k__Bacteria;p__Cyanobacteria	97.10%	33.50%	20.30%
k__Bacteria;p__Firmicutes	0.00%	0.80%	0.00%
k__Bacteria;p__Gemmatimonadetes	0.40%	0.30%	0.60%
k__Bacteria;p__Nitrospirae	0.00%	0.60%	0.50%
k__Bacteria;p__OD1	0.00%	0.00%	0.00%
k__Bacteria;p__Planctomycetes	0.00%	0.00%	0.10%
k__Bacteria;p__Proteobacteria	0.00%	3.20%	8.30%
k__Bacteria;p__TM6	0.00%	0.80%	1.90%
k__Bacteria;p__TM7	0.00%	0.40%	0.20%
k__Bacteria;p__Verrucomicrobia	0.80%	16.00%	20.40%
k__Bacteria;p__[Thermi]	0.00%	0.00%	0.00%
**2. Genus level**			
Taxa	U1 lamina	U2 lamina	U3 lamina
Leptolyngbya	11.10%	4.00%	1.30%
Phormidium	65.80%	20.60%	11.50%
Pseudanabaena	1.80%	0.50%	0.80%
Cyanobacteria other	18.40%	8.40%	6.60%

family_C111	0.10%	16.10%	8.50%
family_Microbacteriaceae	0.50%	9.00%	14.10%
Leifsonia	0.20%	2.30%	3.70%
Yonghaparkia	0.00%	1.40%	2.30%
Iamia	0.00%	1.80%	1.30%
Salinibacterium	0.00%	0.00%	0.50%
Actinobacteria other	0.10%	3.70%	2.70%
family_Opitutaceae	0.10%	15.30%	18.10%
Candidatus Xiphinematobacter	0.20%	0.00%	0.30%
Haloferula	0.20%	0.10%	0.10%
Luteolibacter	0.20%	0.00%	0.10%
Verrucomicrobium	0.00%	0.10%	0.20%
Opitutus	0.00%	0.10%	0.60%
Verrucomicrobia other	0.10%	0.30%	0.90%
family_Xanthomonadaceae	0.00%	0.40%	3.40%
Anaerospora	0.00%	0.20%	0.40%
Rhodobacter	0.00%	0.40%	0.90%
Polaromonas	0.00%	0.00%	0.30%
Mycoplana	0.00%	0.10%	0.10%
Roseococcus	0.00%	0.10%	0.20%
Roseomonas	0.00%	0.00%	0.10%
Pseudoxanthomonas	0.00%	0.10%	0.10%
Thermomonas	0.00%	0.00%	0.20%
Proteobacteria other	0.00%	1.20%	2.10%
family_Cytophagaceae	0.00%	3.00%	6.40%
Sporocytophaga	0.00%	0.20%	0.00%
Bacteroidetes other	0.00%	0.70%	1.60%
Bryobacter	0.00%	0.00%	0.10%
Paenisporosarcina	0.00%	0.60%	0.00%
Gemmatimonas	0.00%	0.20%	0.50%

family_A1-B1	0.40%	0.00%	0.00%
Nitrospira	0.00%	0.60%	0.50%
TM6 other	0.00%	0.80%	1.90%
TM7 other	0.00%	0.30%	0.20%
Bacteria other	0.80%	5.90%	6.00%

Supplementary Table 2: Oligotype ID, Oligotype Sequence, Representative Sequence, Abundance Oligotype, NCBI MEGABLAST results (including average E value and Identity), Taxonomical information (Phylum, Family, and Genus levels) of (a) *Phormidium*, (b) *Leptolyngbya*, (c) Cytophagaceae, (d) Opitutaceae, and (e) Xanthomonadaceae from all three laminae (U1, U2, and U3) of the conical microbial mat from Lake Untersee, Antarctica, used in this study.

Supplementary Table 2(a)

**Oligotype ID**	**Oligotype Sequence**	**Representative Sequence**	**Abundance Oligotype**	**NCBI MEGABLAST Results**	**Average E value**	**Average Identity (%)**	**Phylum**	**Family**	**Genus**
Oligotype 1	CTGATAT GCAT	GAATCTACCTTCAGGACGG AGACAACAGTTGGAAACG ACTGCTAACCCCCGATGTA CCGCAAGGGCAAATATTTA TAGCCTGAAGAAGAGCTCG CGTCCGATTAGCTAGTTGG AGAGGTAAAAGCTCACCAA GGCGACGATCGGTAGCTGG TCTGAGAGGACGATCAGCC ACACTGGGACTGAGACACG GCCCAGACTCCTACGGGAG GCAGCAGTGGGGAATTTTC CGCAATGGGCGAAAGCCTG ACGGAGCAAGACCGCGTG GGGGAAGAAGGCTCTTGGG TTGTAAACCCCTTTTCTCTG GGAAGAAAGTTGTGAAAG CAGCCTGACGGTACCAGAG GAATCAGCAT	1171	Phormidium cf. uncinatum CYN108	9.00E-174	99%	Cyanoba cteria	Oscillatoriac eae	*Phormidium*
				Phormidium sp. Ant-Orange	1.00E-171	99%	Cyanoba cteria	Oscillatoriac eae	*Phormidium*
Oligotype 2	AGACACC CATC	GAATCTACCTTCAGGACGG AGACAACAGTTGGAACGAC TGCTAACCCCCGATGTACC	652	Phormidium cf. uncinatum CYN108	4.00E-172	98%	Cyanoba cteria	Oscillatoriac eae	*Phormidium*

		GCAAGGGCAAATATTTATA<br>GCCTGAAGAAGAGCTCGCG<br>TCCGATTAGCTAGTTGGAG<br>AGGTAAAAGCTCACCAAGG<br>CGACGATCGGTAGCTGGTC<br>TGAGAGGACGATCAGCCAC<br>ACTGGGACTGAGACACGGC<br>CCAGACTCCTACGGGAGGC<br>AGCAGTGGGGAATTTTCCG<br>CAATGGGCGAAAGCCTGAC<br>GGAGCAAGACCGCGTGGG<br>GGAAGAAGGCTCTTGGGTT<br>GTAAACCCCTTTTCTCTGG<br>GAAGAAAGTTGTGAAAGC<br>AGCCTGACGGTACCAGAGG<br>AATCAGCATC							
Oligotype 3	GCTGGTA<br>AGCA	GAATCTACCTTCAGGACGG<br>AGACAACAGTTGGAAACG<br>ACTGCTAACCCCCGACTGT<br>ACCGCAAGGGCAAATATTT<br>ATAGCCTGAAGAAGAGCTC<br>GCGTCCGATTAGCTAGTTG<br>GAGAGGTAAAAGCTCACCA<br>AGGCGACGATCGGTAGCTG<br>GTCTGAGAGGACGATCAGC<br>CACACTGGGACTGAGACAC<br>GGCCCAGACTCCTACGGGA<br>GGCAGCAGTGGGGAATTTT<br>CCGCAATGGGCGAAAGCCT<br>GACGGAGCAAGACCGCGT<br>GGGGGAAGAAGGCTCTTGG<br>GTTGTAAACCCCTTTTCTCT<br>GGGAAGAAAGTTGTGAAA<br>GCAGCCTGACGGTACCAGA<br>GGAATCAGCA	459	Phormidium cf. uncinatum CYN108	1.00E-171	98%	Cyanoba<br>cteria	Oscillatoriac<br>eae	*Phormidium*
Oligotype 4	TGAGTCT<br>CTCC	GAATCTACCTTCAGGACGG<br>AGACAACAGTTGGAAACG<br>ACTGCTAACCCCCGATGTA<br>CCGCAAGGGCAAATATTTA<br>TAGCCTGAAGAAGAGCTCG<br>CGTCCGATTAGCTAGTTGG<br>AGAGGTAAAAGCTCACCAA<br>GGCGACGATCGGTAGCTGG<br>TCTGAGAGGACGATCAGCC	269	Phormidium autumnale Ant-Ph68	2.00E-180	99%	Cyanoba<br>cteria	Oscillatoriac<br>eae	*Phormidium*
				Phormidium autumnale CYN79	9.00E-179	99%	Cyanoba<br>cteria	Oscillatoriac<br>eae	*Phormidium*
				Phormidium autumnale SAG 35.90	4.00E-177	99%	Cyanoba<br>cteria	Oscillatoriac<br>eae	*Phormidium*

		ACACTGGGACTGAGACACGGCCCAGACTCCTACGGGAGGCAGCAGTGGGGAATTTTCCGCAATGGGCGAAAGCCTGACGGAGCAAGACCGCGTGGGGGAAGAAGGCTCTTGGGTTGTAAACCCCTTTTCTCTGGGAAGAACGCAATGACGGTACCAGAGGAATCAGCATCGGCTAACTCC		Phormidium cf. uncinatum CAWBG523	9.00E-179	99%	Cyanobacteria	Oscillatoriaceae	*Phormidium*
				Phormidium sp. LEGE 06363	4.00E-177	99%	Cyanobacteria	Oscillatoriaceae	*Phormidium*
				Phormidium sp. LEGE 07215	4.00E-177	99%	Cyanobacteria	Oscillatoriaceae	*Phormidium*
Oligotype 5	ACCTGGACAGC	GAATCTACCTTCAGGACGGAGACAACAGTTGGAAACGACTGCTAACCCCCGACTGTACCGCAAGGGCAAATATTTATAGCCTGAAGAAGAGCTCGCGTCCGATTAGCTAGTTGGAGAGGTAAAAGACTCACCAAGGCGACGATCGGTAGCTGGTCTGAGAGGACGATCAGCCACACTGGGACTGAGACACGGCCCAGACTCCTACGGGAGGCAGCAGTGGGGAATTTTCCGCAATGGGCGAAAGCCTGACGGAGCAAGACCGCGTGGGGGAAGAAGGCTCTTGGGTTGTAAACCCCTTTTCTCTGGGAAGAAAGTTGTGAAAGCAGCCTGACGGTACCAGAGGAATCAGC	230	Phormidium autumnale CYN79	7.00E-170	98%	Cyanobacteria	Oscillatoriaceae	*Phormidium*
				Phormidium sp. Ant-Orange	1.00E-167	99%	Cyanobacteria	Oscillatoriaceae	*Phormidium*
Oligotype 6	GACGCCAATCG	GAATCTACCTTCAGGACGGAGACAACAGTTGGAACGACTGCTAACCCCCGATGTACCGAAAGGGCAAATATTTATAGCCTGAAGAAGAGCTCGCGTCCGATTAGCTAGTTGGAGAGGTAAAGCTCACCAAGGCGACGATCGGTAGCTGGTCTGAGAGGACGATCAGCCACACTGGGACTGAGACACGGCCCAGACTCCTACGGGAGGCAGCAGTGGGGAATTTTCCGCAATGGGCGAAAGCCTGACGGAGCAAGACCGCGTGGGGGAAGAAGGCTCTTGGGTTGTAAACCCCTTTTCTCTGG	139	Phormidium cf. uncinatum CYN108	1.00E-172	98%	Cyanobacteria	Oscillatoriaceae	*Phormidium*
				Phormidium sp. Ant-Orange	2.00E-170	99%	Cyanobacteria	Oscillatoriaceae	*Phormidium*

		GAAGAAAGTTGTGAAAGC AGCCTGACGGTACCAGAGG AATCAGCATCG							
Oligotype 7	AAGGCAA TCCG	GAATCTACCTTCAGGACGG AGACAACAGTTGGAACGAC TGCTAACCCCCGATGTACC GCAAGGGCAAATATTTATA GCCTGAAGAAGAGCTCGCG TCCGATTAGCTAGTTGGAG AGGTAAAAGCTCACCAAGG CGACGATCGGTAGCTGGTC TGAGAGGACGATCAGCCAC ACTGGGACTGAGACACGGC CCAGACTCCTACGGGAGGC AGCAGTGGGGAATTTTCCG CAATGGGCGAAAGCCTGAC GGAGCAAGACCGCGTGGG GGAAGAAGGCTCTTGGGTT GTAAACCCCTTTTCTCTGG GAAGAACGCAATGACGGT ACCAGAGGAATCAGCATCG GCTAACTCCG	132	Phormidium autumnale Arct-Ph5	2.00E-180	99%	Cyanoba cteria	Oscillatoriac eae	*Phormidium*
				Phormidium autumnale Ant-Ph68	9.00E-179	99%	Cyanoba cteria	Oscillatoriac eae	*Phormidium*
				Phormidium autumnale CYN79	4.00E-177	99%	Cyanoba cteria	Oscillatoriac eae	*Phormidium*
				Phormidium autumnale SAG 35.90	2.00E-175	99%	Cyanoba cteria	Oscillatoriac eae	*Phormidium*
				Phormidium cf. uncinatum CAWBG523	4.00E-177	99%	Cyanoba cteria	Oscillatoriac eae	*Phormidium*
				Phormidium sp. LEGE 06363	2.00E-175	99%	Cyanoba cteria	Oscillatoriac eae	*Phormidium*
				Phormidium sp. LEGE 07215	2.00E-175	99%	Cyanoba cteria	Oscillatoriac eae	*Phormidium*
Oligotype 8	AGCCCGG TCAG	GAATCTACCTTCAGGACGG AGACAACAGTTGGAAACG ACTGCTAACCCCCGACTGT ACCGAAAGGGACAAATATT TATAGCCTGAAGAAGAGCT CGCGTCCGATTAGCTAGTT GGAGAGGTAAAAGACTCA CCAAGGCGACGATCGGTAG CTGGTCTGAGAGGACGATC AGCCACACTGGGACTGAGA CACGGCCCAGACTCCTACG GGAGGCAGCAGTGGGGAA TTTTCCGCAATGGGCGAAA GCCTGACGGAGCAAGACCG CGTGGGGGAAGAAGGCTCT TGGGTTGTAAACCCCTTTTC TCTGGGAAGAAAGTTGTGA AAGCAGCCTGACGGTACCA GAGGAATCAG	114	Phormidium cf. uncinatum CYN108	5.00E-171	98%	Cyanoba cteria	Oscillatoriac eae	*Phormidium*
Oligotype 9	GAGAATC ACTC	GAATCTACCTTCAGGACGG AGACAACAGTTGGAAACG ACTGCTAACCCCCGACTGT	100	Phormidium autumnale Arct-Ph5	7.00E-180	99%	Cyanoba cteria	Oscillatoriac eae	*Phormidium*

		ACCGCAAGGGCAAATATTT ATAGCCTGAAGAAGAGCTC GCGTCCGATTAGCTAGTTG GAGAGGTAAAAGCTCACCA AGGCGACGATCGGTAGCTG GTCTGAGAGGACGATCAGC CACACTGGGACTGAGACAC GGCCCAGACTCCTACGGGA GGCAGCAGTGGGGAATTTT CCGCAATGGGCGAAAGCCT GACGGAGCAAGACCGCGT GGGGGAAGAAGGCTCTTGG GTTGTAAACCCCTTTTCTCT GGGAAGAACGCAATGACG GTACCAGAGGAATCAGCAT CGGCTAACTC		Phormidium autumnale Ant-Ph68	3.00E-178	99%	Cyanobacteria	Oscillatoriaceae	*Phormidium*
				Phormidium autumnale CYN79	1.00E-176	99%	Cyanobacteria	Oscillatoriaceae	*Phormidium*
				Phormidium autumnale SAG 35.90	7.00E-175	99%	Cyanobacteria	Oscillatoriaceae	*Phormidium*
				Phormidium cf. uncinatum CAWBG523	1.00E-176	99%	Cyanobacteria	Oscillatoriaceae	*Phormidium*
				Phormidium sp. LEGE 06363	7.00E-175	99%	Cyanobacteria	Oscillatoriaceae	*Phormidium*
				Phormidium sp. LEGE 07215	7.00E-175	99%	Cyanobacteria	Oscillatoriaceae	*Phormidium*
Oligotype 10	AAGCACGATCA	GAATCTACCTTCAGGACGG AGACAACAGTTGGAAACG ACTGCTAACCCCCGACTGT ACCGAAAGGGACAAATAA TTTATAGCCTGAAGAAGAG CTCGCGTCCGATTAGCTAG TTGGAGAGGTAAAAGACTC ACCAAGGCGACGATCGGTA GCTGGTCTGAGAGGACGAT CAGCCACACTGGGACTGAG ACACGGCCCAGACTCCTAC GGGAGGCAGCAGTGGGGA ATTTTCCGCAATGGGCGAA AGCCTGACGGAGCAAGACC GCGTGGGGGAAGAAGGCT CTTGGGTTGTAAACCCCTTT TCTCTGGGAAGAAAGTTGT GAAAGCAGCCTGACGGTAC CAGAGGAATCA	65	Phormidium cf. uncinatum CYN108	2.00E-169	98%	Cyanobacteria	Oscillatoriaceae	*Phormidium*
				Phormidium sp. Ant-Orange	2.00E-165	99%	Cyanobacteria	Oscillatoriaceae	*Phormidium*
Oligotype 11	GCAGAAGAACT	GAATCTACCTTCAGGACGG AGACAACAGTTGGAAACG ACTGCTAACCCCCGACTGT ACCGCAAGGGCAAATATTT ATAGCCTGAAGAAGAGCTC GCGTCCGATTAGCTAGTTG GAGAGGTAAAAGACTCACC AAGGCGACGATCGGTAGCT GGTCTGAGAGGACGATCAG	46	Phormidium autumnale Arct-Ph5	3.00E-178	99%	Cyanobacteria	Oscillatoriaceae	*Phormidium*
				Phormidium autumnale Ant-Ph68	1.00E-176	99%	Cyanobacteria	Oscillatoriaceae	*Phormidium*
				Phormidium autumnale CYN79	7.00E-175	99%	Cyanobacteria	Oscillatoriaceae	*Phormidium*

		CCACACTGGGACTGAGACACGGCCCAGACTCCTACGGGAGGCAGCAGTGGGGAATTTTCCGCAATGGGCGAAAGCCTGACGGAGCAAGACCGCGTGGGGGAAGAAGGCTCTTGGGTTGTAAACCCCTTTTCTCTGGGAAGAACGCAATGACGGTACCAGAGGAATCAGCATCGGCTAACT		Phormidium autumnale SAG 35.90	3.00E-173	99%	Cyanobacteria	Oscillatoriaceae	*Phormidium*
				Phormidium cf. uncinatum CAWBG523	7.00E-175	99%	Cyanobacteria	Oscillatoriaceae	*Phormidium*
				Phormidium sp. LEGE 06363	3.00E-173	99%	Cyanobacteria	Oscillatoriaceae	*Phormidium*
				Phormidium sp. LEGE 07215	3.00E-173	99%	Cyanobacteria	Oscillatoriaceae	*Phormidium*
Oligotype 12	GAATTAAAACA	TGGGGAATTTTCCGCAATGGGCGAAAGCCTGACGGAGCAAGACCGCGTGGGGGAAGAAGGCTCTTGGGTTGTAAACCCCTTTTCTCTGGGAAGAAAGTTGTGAAAGCAGCCTGACGGTACCAGAGGAATCAGCATCGGCTAACTCCGTGCCAGCAGCCGCGGTAAGACGGAGGATGCAAGCGTTATCCGGAATGATTGGGCGTAAAGCGTCCGCAGGTGGCAGTTCAAGTCTGCTGTCAAAGACCGGGGCTCAACCTCGGAAAGGCAGTGGAAACTGAACAGCTAGAGTATGGTAGGGGCAAAGGGAATTCCTGGTGTAGCGGTGAAATGCGTAGAGATCAGGAAGAACA	35	Phormidium cf. uncinatum CYN108	2.00E-180	99%	Cyanobacteria	Oscillatoriaceae	*Phormidium*
				Phormidium uncinatum PACC 8693	9.00E-179	99%	Cyanobacteria	Oscillatoriaceae	*Phormidium*
				Phormidium cf. autumnale JR8	2.00E-180	99%	Cyanobacteria	Oscillatoriaceae	*Phormidium*
				Phormidium cf. autumnale CCALA 853	2.00E-180	99%	Cyanobacteria	Oscillatoriaceae	*Phormidium*
				Phormidium sp. KU003	9.00E-179	99%	Cyanobacteria	Oscillatoriaceae	*Phormidium*
Oligotype 13	GCAGGAAAATC	GAATCTACCTTCAGGACGGAGACAACAGTTGGAAACGACTGCTAACCCCCGACTGTACCGAAAGGGACGAAATAATTTATAGCCTGAAGAAGAGCTCGCGTCCGATTAGCTAGTTGGAGAGGTAAAAGACTCACCAAGGCGACGATCGGTAGCTGGTCTGAGAGGACGATCAGCCACACTGGGACTGAGACACGGCCCAGACTCCTACGGGAGGCAGCAGTGGGGAATTTTCCGCAATGGGCGAAAGCCTGACGGAGCAAGACCGCGTGGGGGAAGAAGG	34	Phormidium cf. uncinatum CYN108	4.00E-167	98%	Cyanobacteria	Oscillatoriaceae	*Phormidium*

		CTCTTGGGTTGTAAACCCC TTTTCTCTGGGAAGAAAGT TGTGAAAGCAGCCTGACGG TACCAGAGGAATC							
Oligotype 14	CAAAGTG GAAC	TGGGGAATTTTCCGCAATG GGCGAAAAGCCTGACGGA GCAAGACCGCGTGGGGA AGAAGGCTCTTGGGTTGTA AACCCCTTTTCTCTGGGAA GAAAGTTGTGAAAGCAGCC TGACGGTACCAGAGGAATC AGCATCGGCTAACTCCGTG CCAGCAGCCGCGGTAAGAC GGAGGATGCAAGCGTTATC CGGAATGATTGGGCGTAAA GCGTCCGCAGGTGGCAGTT CAAGTCTGCTGTCAAAGAC CGGGGCTCAACCTCGGAAA GGCAGTGGAAACTGAACA GCTAGAGTATGGTAGGGGC AAAGGGAATTCCTGGTGTA GCGGTGAAATGCGTAGAGA TCAGGAAGAAC	32	Phormidium sp. Ant-Orange	7.00E-180	99%	Cyanobacteria	Oscillatoriaceae	*Phormidium*
				Phormidium sp. Ant-Lunch	7.00E-180	99%	Cyanobacteria	Oscillatoriaceae	*Phormidium*
				Phormidium cf. uncinatum CYN108	3.00E-178	99%	Cyanobacteria	Oscillatoriaceae	*Phormidium*
				Phormidium cf. autumnale JR8	3.00E-178	99%	Cyanobacteria	Oscillatoriaceae	*Phormidium*
				Phormidium cf. autumnale CCALA 853	3.00E-178	99%	Cyanobacteria	Oscillatoriaceae	*Phormidium*
				Phormidium uncinatum PACC 8693	1.00E-176	99%	Cyanobacteria	Oscillatoriaceae	*Phormidium*
				Phormidium sp. KU003	1.00E-176	99%	Cyanobacteria	Oscillatoriaceae	*Phormidium*
Oligotype 15	CGGAAGA CCGT	GAATCTACCTTCAGGACGG AGACAACAGTTGGAACGAC TGCTAACCCCCGATGTACC GCAAGGGCAAATATTTATA GCCTGAAGAAGAGCTCGCG TCCGATTAGCTAGTTGGAG AGGTAAAGCTCACCAAGGC GACGATCGGTAGCTGGTCT GAGAGGACGATCAGCCAC ACTGGGACTGAGACACGGC CCAGACTCCTACGGGAGGC AGCAGTGGGGAATTTTCCG CAATGGGCGAAAGCCTGAC GGAGCAAGACCGCGTGGG GGAAGAAGGCTCTTGGGTT GTAAACCCCTTTTCTCTGG GAAGAACGCAATGACGGT ACCAGAGGAATCAGCATCG GCTAACTCCGT	31	Phormidium autumnale Arct-Ph5	2.00E-179	99%	Cyanobacteria	Oscillatoriaceae	*Phormidium*
				Phormidium autumnale Ant-Ph68	1.00E-177	99%	Cyanobacteria	Oscillatoriaceae	*Phormidium*
				Phormidium autumnale CYN79	5.00E-176	99%	Cyanobacteria	Oscillatoriaceae	*Phormidium*
				Phormidium autumnale SAG 35.90	2.00E-174	99%	Cyanobacteria	Oscillatoriaceae	*Phormidium*
				Phormidium cf. uncinatum CAWBG523	5.00E-176	99%	Cyanobacteria	Oscillatoriaceae	*Phormidium*
				Phormidium sp. LEGE 06363	2.00E-174	99%	Cyanobacteria	Oscillatoriaceae	*Phormidium*
				Phormidium sp. LEGE 07215	2.00E-174	99%	Cyanobacteria	Oscillatoriaceae	*Phormidium*
Oligotype 16	CTGATAC CATC	GAATCTACCTTCAGGACGG AGACAACAGTTGGAAACG	19	Phormidium cf. uncinatum	9.00E-174	99%	Cyanobacteria	Oscillatoriaceae	*Phormidium*

		ACTGCTAACCCCCGATGTA CCGAAAGGGCAAATATTTA TAGCCTGAAGAAGAGCTCG CGTCCGATTAGCTAGTTGG AGAGGTAAAAGCTCACCAA GGCGACGATCGGTAGCTGG TCTGAGAGGACGATCAGCC ACACTGGGACTGAGACACG GCCCAGACTCCTACGGGAG GCAGCAGTGGGGAATTTTC CGCAATGGGCGAAAGCCTG ACGGAGCAAGACCGCGTG GGGGAAGAAGGCTCTTGGG TTGTAAACCCCTTTTCTCTG GGAAGAAAGTTGTGAAAG CAGCCTGACGGTACCAGAG GATCAGCATC		CYN108					
Oligotype 17	GGAACGG AGAA	TGGGGAATTTTTCCGCAAT GGGCGAAAAGCCTGACGG AGCAAGACCGCGTGGGGG AAGAAGGCTCTTGGGTTGT AAACCCCTTTTCTCTGGGA AGAAAGTTGTGAAAGCAGC CTGACGGTACCAGAGGAAT CAGCATCGGCTAACTCCGT GCCAGCAGCCGCGGTAAGA CGGAGGATGCAAGCGTTAT CCGGAATGATTGGGCGTAA AGCGTCCGCAGGTGGCAGT TCAAGTCTGCTGTCAAAGA CCGGGGCTCAACCTCGGAA AGGCAGTGGAAACTGAAC AGCTAGAGTATGGTAGGGG CAAAGGGAATTCCTGGTGT AGCGGTGAAATGCGTAGAG ATCAGGAAGAA	15	Phormidium sp. Ant-Orange	3.00E-178	99%	Cyanoba cteria	Oscillatoriac eae	*Phormidium*
				Phormidium sp. Ant-Lunch	3.00E-178	99%	Cyanoba cteria	Oscillatoriac eae	*Phormidium*
				Phormidium cf. uncinatum CYN108	1.00E-176	99%	Cyanoba cteria	Oscillatoriac eae	*Phormidium*
				Phormidium uncinatum PACC 8693	7.00E-175	99%	Cyanoba cteria	Oscillatoriac eae	*Phormidium*
				Phormidium cf. autumnale JR8	1.00E-176	99%	Cyanoba cteria	Oscillatoriac eae	*Phormidium*
				Phormidium cf. autumnale CCALA 853	1.00E-176	99%	Cyanoba cteria	Oscillatoriac eae	*Phormidium*
				Phormidium sp. KU003	7.00E-175	99%	Cyanoba cteria	Oscillatoriac eae	*Phormidium*
Oligotype 18	GATGAGA ACAT	TGGGGAATTTTCCGCAATG GGCGAAAGCCTGACGGAG CAAGACCGCGTGGGGGAA GAAGGCTCTTGGGTTGTAA ACCCCTTTTCTCTGGGAAG AAAGTTGTGAAAGCAGCCT GACGGTACCAGAGGAATCA GCATCGGCTAACTCCGTGC	12	Phormidium sp. Ant-Orange	2.00E-180	99%	Cyanoba cteria	Oscillatoriac eae	*Phormidium*
				Phormidium sp. Ant-Lunch	2.00E-180	99%	Cyanoba cteria	Oscillatoriac eae	*Phormidium*
				Phormidium cf. uncinatum CYN108	9.00E-179	99%	Cyanoba cteria	Oscillatoriac eae	*Phormidium*
				Phormidium	4.00E-177	99%	Cyanoba	Oscillatoriac	*Phormidium*

		CAGCAGCCGCGGTAAGACG GAGGATGCAAGCGTTATCC GGAATGATTGGGCGTAAAG CGTCCGCAGGTGGCAGTTC AAGTCTGCTGTCAAAGACC GGGCTCAACCTCGGAAAGG CAGTGGAAACTGAACAGCT AGAGTATGGTAGGGGCAA AGGGAATTCCTGGTGTAGC GGTGAAATGCGTAGAGATC AGGAAGAACAT		uncinatum PACC 8693			cteria	eae	
				Phormidium cf. autumnale JR8	9.00E-179	99%	Cyanobacteria	Oscillatoriaceae	*Phormidium*
				Phormidium cf. autumnale CCALA 853	9.00E-179	99%	Cyanobacteria	Oscillatoriaceae	*Phormidium*
				Phormidium sp. KU003	4.00E-177	99%	Cyanobacteria	Oscillatoriaceae	*Phormidium*
Oligotype 19	TATCAATCGAA	TGGGGAATTTTCCGCAATG GGCGAAAGCCTGACGGAG CAAGACCGCGTGGGGGAA GAAGGCTCTTGGGTTGTAA ACCCCTTTTCTCTGGGAAG AACGCAATGACGGTACCAG AGGAATCAGCATCGGCTAA CTCCGTGCCAGCAGCCGCG GTAAGACGGAGGATGCAA GCGTTATCCGGAATGATTG GGCGTAAAGCGTCCGCAGG TGGCAGTTCAAGTCTGCTG TCAAAGACCGGGGCTCAAC CTCGGAAAGGCAGTGGAA ACTGAACAGCTAGAGTATG GTAGGGGCAAAGGGAATTC CTGGTGTAGCGGTGAAATG CGTAGAGATCAGGAAGAA CATCGGTGGCGAA	9	Phormidium cf. uncinatum CAWBG523	2.00E-180	99%	Cyanobacteria	Oscillatoriaceae	*Phormidium*
				Phormidium autumnale KNUA026	2.00E-180	99%	Cyanobacteria	Oscillatoriaceae	*Phormidium*
				Phormidium cf. autumnale CCALA 844	2.00E-180	99%	Cyanobacteria	Oscillatoriaceae	*Phormidium*
				Phormidium cf. autumnale CCALA 852	4.00E-177	99%	Cyanobacteria	Oscillatoriaceae	*Phormidium*
				Phormidium autumnale VUW20	2.00E-180	99%	Cyanobacteria	Oscillatoriaceae	*Phormidium*
				Phormidium autumnale VUW9	2.00E-180	99%	Cyanobacteria	Oscillatoriaceae	*Phormidium*
				Phormidium autumnale VUW11	2.00E-180	99%	Cyanobacteria	Oscillatoriaceae	*Phormidium*
				Phormidium autumnale VUW21	9.00E-179	99%	Cyanobacteria	Oscillatoriaceae	*Phormidium*
				Phormidium autumnale VUW24	9.00E-179	99%	Cyanobacteria	Oscillatoriaceae	*Phormidium*
				Phormidium autumnale VUW19	9.00E-179	99%	Cyanobacteria	Oscillatoriaceae	*Phormidium*
				Phormidium autumnale VUW16	9.00E-179	99%	Cyanobacteria	Oscillatoriaceae	*Phormidium*

				Phormidium autumnale VUW2	9.00E-179	99%	Cyanobacteria	Oscillatoriaceae	*Phormidium*
				Phormidium autumnale CYN79	9.00E-179	99%	Cyanobacteria	Oscillatoriaceae	*Phormidium*
				Phormidium autumnale CYN119	9.00E-179	99%	Cyanobacteria	Oscillatoriaceae	*Phormidium*
				Phormidium autumnale CYN55	9.00E-179	99%	Cyanobacteria	Oscillatoriaceae	*Phormidium*
				Phormidium autumnale Ant-Ph68	1.00E-177	99%	Cyanobacteria	Oscillatoriaceae	*Phormidium*
				Phormidium cf. uncinatum CAWBG527	9.00E-179	99%	Cyanobacteria	Oscillatoriaceae	*Phormidium*
				Phormidium sp. LEGE 06363	4.00E-177	99%	Cyanobacteria	Oscillatoriaceae	*Phormidium*
				Phormidium sp. CCALA 726	4.00E-177	99%	Cyanobacteria	Oscillatoriaceae	*Phormidium*
				Phormidium cf. setchellianum CCALA 149	4.00E-177	99%	Cyanobacteria	Oscillatoriaceae	*Phormidium*
				Phormidium cf. irriguum CCALA 815	4.00E-177	99%	Cyanobacteria	Oscillatoriaceae	*Phormidium*
Oligotype 20	GGATGAGGCGA	TGGGGAATTTTTCCGCAATGGGCGAAAGCCTGACGGAGCAAGACCGCGTGGGGGAAGAAGGCTCTTGGGTTGTAAACCCCTTTTCTCTGGGAAGAACGCAATGACGGTACCAGAGGAATCAGCATCGGCTAACTCCGTGCCAGCAGCCGCGGTAAGACGGAGGATGCAAGCGTTATCCGGAATGATTGGGCGTAAAGCGTCCGCAGGTGGCAGTTCAAGTCTGCTGTCAAAGACCGGGGCTCAACCTCGGAAAGGCAGTGGAAACTGAACAGCTAGAGTATG	6	Phormidium cf. autumnale CCALA 854	7.00E-180	99%	Cyanobacteria	Oscillatoriaceae	*Phormidium*
				Phormidium cf. autumnale CCALA 852	7.00E-175	99%	Cyanobacteria	Oscillatoriaceae	*Phormidium*
				Phormidium cf. autumnale CCALA 844	3.00E-178	99%	Cyanobacteria	Oscillatoriaceae	*Phormidium*
				Phormidium autumnale CCALA 697	7.00E-180	99%	Cyanobacteria	Oscillatoriaceae	*Phormidium*
				Phormidium autumnale CCAP 1462/10	7.00E-180	99%	Cyanobacteria	Oscillatoriaceae	*Phormidium*

		GTAGGGGCAAAGGGAATTC CTGGTGTAGCGGTGAAATG CGTAGAGATCAGGAAGAA CATCGGTGGCGA		Phormidium autumnale Ant-Ph68	2.00E-175	99%	Cyanobacteria	Oscillatoriaceae	*Phormidium*
				Phormidium autumnale Arct-Ph5	7.00E-180	99%	Cyanobacteria	Oscillatoriaceae	*Phormidium*
				Phormidium autumnale KNUA026	3.00E-178	99%	Cyanobacteria	Oscillatoriaceae	*Phormidium*
				Phormidium autumnale VUW12	7.00E-180	99%	Cyanobacteria	Oscillatoriaceae	*Phormidium*
				Phormidium autumnale VUW20	3.00E-178	99%	Cyanobacteria	Oscillatoriaceae	*Phormidium*
				Phormidium autumnale VUW9	3.00E-178	99%	Cyanobacteria	Oscillatoriaceae	*Phormidium*
				Phormidium autumnale VUW11	3.00E-178	99%	Cyanobacteria	Oscillatoriaceae	*Phormidium*
				Phormidium autumnale VUW19	1.00E-176	99%	Cyanobacteria	Oscillatoriaceae	*Phormidium*
				Phormidium autumnale VUW24	1.00E-176	99%	Cyanobacteria	Oscillatoriaceae	*Phormidium*
				Phormidium autumnale VUW21	1.00E-176	99%	Cyanobacteria	Oscillatoriaceae	*Phormidium*
				Phormidium autumnale VUW2	1.00E-176	99%	Cyanobacteria	Oscillatoriaceae	*Phormidium*
				Phormidium autumnale VUW16	1.00E-176	99%	Cyanobacteria	Oscillatoriaceae	*Phormidium*
				Phormidium autumnale CYN79	1.00E-176	99%	Cyanobacteria	Oscillatoriaceae	*Phormidium*
				Phormidium autumnale CYN119	1.00E-176	99%	Cyanobacteria	Oscillatoriaceae	*Phormidium*
				Phormidium autumnale	1.00E-176	99%	Cyanobacteria	Oscillatoriaceae	*Phormidium*

				CYN55					
				Phormidium cf. uncinatum CCALA 849	7.00E-180	99%	Cyanobacteria	Oscillatoriaceae	*Phormidium*
				Phormidium cf. uncinatum CAWBG523	3.00E-178	99%	Cyanobacteria	Oscillatoriaceae	*Phormidium*
				Phormidium cf. uncinatum CAWBG527	1.00E-176	99%	Cyanobacteria	Oscillatoriaceae	*Phormidium*
				Phormidium sp. CCALA 845	7.00E-180	99%	Cyanobacteria	Oscillatoriaceae	*Phormidium*
				Phormidium sp. CCALA 846	7.00E-180	99%	Cyanobacteria	Oscillatoriaceae	*Phormidium*
				Phormidium sp. CCALA 726	7.00E-175	99%	Cyanobacteria	Oscillatoriaceae	*Phormidium*
				Phormidium sp. LEGE 06363	7.00E-175	99%	Cyanobacteria	Oscillatoriaceae	*Phormidium*
				Phormidium cf. setchellianum CCALA 149	7.00E-175	99%	Cyanobacteria	Oscillatoriaceae	*Phormidium*
				Phormidium cf. irriguum CCALA 815	7.00E-175	99%	Cyanobacteria	Oscillatoriaceae	*Phormidium*

Supplementary Table 2(b)

**Oligotype ID**	**Oligotype Sequence**	**Representative Sequence**	**Abundance Oligotype**	**NCBI MEGABLAST Results**	**Average E value**	**Average Identity (%)**	**Phylum**	**Family**	**Genus**
Oligotype 1	ACCC	GGATCTGCCCTTAGGACGGGGACAACAGCTGGAACGGCTGCTAATACCCGATGTGCCGAGAGGTGAAACTATTTGGCCTGAGGATGAGCTCGCGTCTGATTAGCTAGTTGGAGTGGTAACGGCACACCAAGGCGACGATCAGTAGCTGGTCTGAGAGGATGATCAGCCACACTGGGACTGAGACACGGCCCAGACTCCTACGGGAGGC	116	Leptolyngbya antarctica ANT.L18.1	2.00E-175	99%	Cyanobacteria	Oscillatoriaceae	*Leptolyngbya*
				Leptolyngbya antarctica ANT.L18.2	2.00E-175	99%	Cyanobacteria	Oscillatoriaceae	*Leptolyngbya*
				Leptolyngbya antarctica ANT.LWA.1	2.00E-175	99%	Cyanobacteria	Oscillatoriaceae	*Leptolyngbya*
				Leptolyngbya antarctica	2.00E-175	99%	Cyanobacteria	Oscillatoriaceae	*Leptolyngbya*

		AGCAGTGGGGAATTTTCCGCAATGGGCGAAAGCCTGACGGAGCAAGACCGCGTGCGGGAGGAAGGTCTATTGATTGTAAACCGCTTTTGATAGGGAAGAAGATCTGACGGTACCTATCGAATCAGCATCGGCTAACTCCGTG		ANT.LG2.5					
				Leptolyngbya antarctica ANT.L67.1	2.00E-175	99%	Cyanobacteria	Oscillatoriaceae	*Leptolyngbya*
				Leptolyngbya antarctica ANT.LG2.3	1.00E-172	99%	Cyanobacteria	Oscillatoriaceae	*Leptolyngbya*
				Leptolyngbya sp. CENA538	7.00E-160	96%	Cyanobacteria	Oscillatoriaceae	*Leptolyngbya*
				Uncultured Leptolyngbya sp. clone	2.00E-156	96%	Cyanobacteria	Oscillatoriaceae	*Leptolyngbya*
Oligotype 2	TTTT	GGATCTGCCCTTAGGACGGGGACAACAGCTGGAAACGGCTGCTAATACCCGATGTGCCGAGAGGTGAAACTATTTGGCCTGAGGATGAGCTCGCGTCTGATTAGCTAGTTGGAGTGGTAACGGCACACCAAGGCGACGATCAGTAGCTGGTCTGAGAGGATGATCAGCCACACTGGGACTGAGACACGGCCCAGACTCCTACGGGAGGCAGCAGTGGGGAATTTTCCGCAATGGGCGAAAGCCTGACGGAGCAAGACCGCGTGCGGGAGGAAGGTCTATTGATTGTAAACCGCTTTTGATAGGGAAGAAGATCTGACGGTACCTATCGAATCAGCATCGGCTAACTCCGT	109	Leptolyngbya antarctica ANT.L18.1	4.00E-177	99%	Cyanobacteria	Oscillatoriaceae	*Leptolyngbya*
				Leptolyngbya antarctica ANT.L18.2	4.00E-177	99%	Cyanobacteria	Oscillatoriaceae	*Leptolyngbya*
				Leptolyngbya antarctica ANT.LWA.1	4.00E-177	99%	Cyanobacteria	Oscillatoriaceae	*Leptolyngbya*
				Leptolyngbya antarctica ANT.LG2.5	4.00E-177	99%	Cyanobacteria	Oscillatoriaceae	*Leptolyngbya*
				Leptolyngbya antarctica ANT.L67.1	4.00E-177	99%	Cyanobacteria	Oscillatoriaceae	*Leptolyngbya*
				Leptolyngbya antarctica ANT.LG2.3	2.00E-174	99%	Cyanobacteria	Oscillatoriaceae	*Leptolyngbya*
				Leptolyngbya sp. CENA538	5.00E-161	97%	Cyanobacteria	Oscillatoriaceae	*Leptolyngbya*
				Uncultured Leptolyngbya sp. clone	3.00E-158	96%	Cyanobacteria	Oscillatoriaceae	*Leptolyngbya*
Oligotype 3	GTGG	GGATCTGCCCTTAGGACGGGGACAACAGCTGGAAACGGCTGCTAATACCCGATGTGCCGAGAGGTGAAACTATTTGGCCTGAGGATGAGCTCGCGTCTGATTAGCTAGTTGGAGTGGTAACGGCACACCAAGGCGACGATCAGTAGCTGGTCTGAGAGGATGATCAGCCA	74	Uncultured cyanobacterium clone	6.00E-141	98%	Cyanobacteria	-	-
				Oscillatoria amoena CCAP	1.00E-142	93%	Cyanobacteria	Oscillatoriaceae	*Oscillatoria*

		CACTGGGACTGAGACACGG<br>CCCAGACTCCTACGGGAGG<br>CAGCAGTGGGGAATTTTCC<br>GCAATGGGCGAAAGCCTGA<br>CGGAGCAAGACCGCGTGG<br>GGGAAGAAGGCTCTTGGGT<br>TGTAAACCCCTTTTCTCTGG<br>GAAGAAAGTTGTGAAAGC<br>AGCCTGACGGTACCAGAGG<br>AATCAGCATC							
Oligotype 4	CGTA	GGATCTGCCCTTAGGACGG<br>GGACAACAGCTGGAACGG<br>CTGCTAATACCCGATGTGC<br>CGAGAGGTGAAACTATTTG<br>GCCTGAGGATGAGCTCGCG<br>TCTGATTAGCTAGTTGGAG<br>TGGTAACGGCACACCAAGG<br>CGACGATCAGTAGCTGGTC<br>TGAGAGGATGATCAGCCAC<br>ACTGGGACTGAGACACGGC<br>CCAGACTCCTACGGGAGGC<br>AGCAGTGGGGAATTTTCCG<br>CAATGGGCGAAAGCCTGAC<br>GGAGCAAGACCGCGTGGG<br>GGAAGAAGGCTCTTGGGTT<br>GTAAACCCCTTTTCTCTGG<br>GAAGAAAGTTGTGAAAGC<br>AGCCTGACGGTACCAGAGG<br>AATCAGCATCG	69	Uncultured cyanobacterium clone	2.00E-140	96%	Cyanobacteria	-	-
				Oscillatoria amoena CCAP	2.00E-141	93%	Cyanobacteria	Oscillatoriaceae	*Oscillatoria*
				Microcoleus vaginatus	7.00E-140	93%	Cyanobacteria	Microcoleaceae	*Microcoleus*
Oligotype 5	CGAG	GGATCTGCCCTTAGGACGG<br>GGACAACAGCTGGAAACG<br>GCTGCTAATACCCGATGTG<br>CCGAGAGGTGAAACTATTT<br>GGCCTGAGGATGAGCTCGC<br>GTCTGATTAGCTAGTTGGA<br>GTGGTAACGGCACACCAAG<br>GCGACGATCAGTAGCTGGT<br>CTGAGAGGATGATCAGCCA<br>CACTGGGACTGAGACACGG<br>CCCAGACTCCTACGGGAGG<br>CAGCAGTGGGAATTTCCGC<br>AATGGGCGAAAGCCTGACG<br>GAGCAAGACCGCGTGCGG<br>GAGGAAGGTCTATTGATTG	57	Uncultured cyanobacterium clone	2.00E-179	99%	Cyanobacteria	-	-
				Leptolyngbya antarctica ANT.L18.1	2.00E-174	99%	Cyanobacteria	Oscillatoriaceae	*Leptolyngbya*
				Leptolyngbya antarctica ANT.L18.2	2.00E-174	99%	Cyanobacteria	Oscillatoriaceae	*Leptolyngbya*
				Leptolyngbya antarctica ANT.LWA.1	2.00E-174	99%	Cyanobacteria	Oscillatoriaceae	*Leptolyngbya*
				Leptolyngbya antarctica ANT.LG2.5	2.00E-174	99%	Cyanobacteria	Oscillatoriaceae	*Leptolyngbya*

		TAAACCGCTTTTGATAGGGAAGAAGATCTGACGGTACCTATCGAATCAGCATCGGCTAACTCCGTGC		Leptolyngbya antarctica ANT.L67.1	2.00E-174	99%	Cyanobacteria	Oscillatoriaceae	*Leptolyngbya*
				Leptolyngbya antarctica ANT.LG2.3	2.00E-171	99%	Cyanobacteria	Oscillatoriaceae	*Leptolyngbya*
				Leptolyngbya sp. CENA538	3.00E-158	96%	Cyanobacteria	Oscillatoriaceae	*Leptolyngbya*
				Uncultured cyanobacterium clone	2.00E-155	95%	Cyanobacteria	-	-
Oligotype 6	GAAA	GGATCTGCCCTTAGGACGGGGACAACAGCTGGAAACGGCTGCTAATACCCGATGTGCCGAGAGGTGAAACTATTTGGCCTGAGGATGAGCTCGCGTCTGATTAGCTAGTTGGAGTGGTAACGGCACACCAAGGCGACGATCAGTAGCTGGTCTGAGAGGATGATCAGCCACACTGGGACTGAGACACGGCCCAGACTCCTACGGGAGGCAGCAGTGGGGAATTTTCCGCAATGGGCGAAAGCCTGACGGAGCAAGACCGCGTGCGGGAGGAAGGTCTATTGATTGTAAACCGCTTTTTGATAGGGAAGAAGATCTGACGGTACCTATCGAATCAGCATCGGCTAACTCCG	38	Uncultured cyanobacterium clone	7.00E-180	99%	Cyanobacteria	-	-
				Leptolyngbya antarctica ANT.L18.1	7.00E-175	99%	Cyanobacteria	Oscillatoriaceae	*Leptolyngbya*
				Leptolyngbya antarctica ANT.L18.2	7.00E-175	99%	Cyanobacteria	Oscillatoriaceae	*Leptolyngbya*
				Leptolyngbya antarctica ANT.LWA.1	7.00E-175	99%	Cyanobacteria	Oscillatoriaceae	*Leptolyngbya*
				Leptolyngbya antarctica ANT.LG2.5	7.00E-175	99%	Cyanobacteria	Oscillatoriaceae	*Leptolyngbya*
				Leptolyngbya antarctica ANT.L67.1	7.00E-175	99%	Cyanobacteria	Oscillatoriaceae	*Leptolyngbya*
				Leptolyngbya antarctica ANT.LG2.3	4.00E-172	99%	Cyanobacteria	Oscillatoriaceae	*Leptolyngbya*
				Leptolyngbya sp. CENA538	3.00E-159	96%	Cyanobacteria	Oscillatoriaceae	*Leptolyngbya*
Oligotype 7	AAAG	GGATCTGCCCTTAGGACGGGGACAACAGCTGGAACGGCTGCTAATACCCGATGTGCCGAGAGGTGAAACTATTTGGCCTGAGGATGAGCTCGCGTCTGATTAGCTAGTTGGAGTGGTAACGGCACACCAAGGCGACGATCAGTAGCTGGTCTGAGAGGATGATCAGCCACACTGGGACTGAGACACGGC	30	Uncultured cyanobacterium clone	9.00E-139	96%	Cyanobacteria	-	-
				Oscillatoria amoena CCAP	7.00E-140	93%	Cyanobacteria	Oscillatoriaceae	*Oscillatoria*

		CCAGACTCCTACGGGAGGC AGCAGTGGGGAATTTTCCG CAATGGGCGAAAGCCTGAC GGAGCAAGACCGCGTGGG GGAAGAAGGCTCTTGGGTT GTAAACCCCTTTCTCTGGG AAGAAAGTTGTGAAAGCA GCCTGACGGTACCAGAGGA ATCAGCATCGG							
Oligotype 8	CAGG	GGATCTGCCCTTAGGACGG GGACAACAGCTGGAAACG GCTGCTAATACCCGATGTG CCGAGAGGTGAAACTATTT GGCCTGAGGATGAGCTCGC GTCTGATTAGCTAGTTGGA GTGGTAACGGCACACCAAG GCGACGATCAGTAGCTGGT CTGAGAGGATGATCAGCCA CACTGGGACTGAGACACGG CCCAGACTCCTACGGGAGC AGCAGTGGGAATTTCCGCA ATGGGCGAAAGCCTGACGG AGCAAGACCGCGTGCGGG AGGAAGGTCTATTGATTGT AAACCGCTTTTGATAGGGA AGAAGATCTGACGGTACCT ATCGAATCAGCATCGGCTA ACTCCGTGCC	24	Uncultured cyanobacterium clone	1.00E-172	98%	Cyanobacteria		-
				Leptolyngbya antarctica ANT.L18.1	1.00E-172	98%	Cyanobacteria	Oscillatoriaceae	*Leptolyngbya*
				Leptolyngbya antarctica ANT.L18.2	1.00E-172	98%	Cyanobacteria	Oscillatoriaceae	*Leptolyngbya*
				Leptolyngbya antarctica ANT.LWA.1	1.00E-172	98%	Cyanobacteria	Oscillatoriaceae	*Leptolyngbya*
				Leptolyngbya antarctica ANT.LG2.5	1.00E-172	98%	Cyanobacteria	Oscillatoriaceae	*Leptolyngbya*
				Leptolyngbya antarctica ANT.L67.1	1.00E-172	98%	Cyanobacteria	Oscillatoriaceae	*Leptolyngbya*
				Leptolyngbya antarctica ANT.LG2.3	7.00E-170	98%	Cyanobacteria	Oscillatoriaceae	*Leptolyngbya*
				Leptolyngbya sp. CENA538	4.00E-157	96%	Cyanobacteria	Oscillatoriaceae	*Leptolyngbya*
Oligotype 9	TACC	GGATCTGCCCTTAGGACGG GGACAACAGCTGGAAACG GCTGCTAATACCCGATGTG CCGAGAGGTGAAACTATTT GGCCTGAGGATGAGCTCGC GTCTGATTAGCTAGTTGGA GTGGTAACGGCACACCAAG GCGACGATCAGTAGCTGGT CTGAGAGGATGATCAGCCA CACTGGGACTGAGACACGG CCCAGACTCCTACGGGAGC AGCAGTGGGAATTTCCGCA	17	Uncultured cyanobacterium clone	1.00E-176	99%	Cyanobacteria	-	-
				Leptolyngbya antarctica ANT.L18.1	2.00E-171	98%	Cyanobacteria	Oscillatoriaceae	*Leptolyngbya*
				Leptolyngbya antarctica ANT.L18.2	2.00E-171	98%	Cyanobacteria	Oscillatoriaceae	*Leptolyngbya*
				Leptolyngbya antarctica ANT.LWA.1	2.00E-171	98%	Cyanobacteria	Oscillatoriaceae	*Leptolyngbya*

		ATGGCGAAAGCCTGACGGAGCAAGACCGCGTGCGGGAGGAAGGTCTATTGATTGTAAACCGCTTTTGATAGGGAAGAAGATCTGACGGTACCTATCGAATCAGCATCGGCTAACTCCGTGCCA		Leptolyngbya antarctica ANT.LG2.5	2.00E-171	98%	Cyanobacteria	Oscillatoriaceae	*Leptolyngbya*
				Leptolyngbya antarctica ANT.L67.1	2.00E-171	98%	Cyanobacteria	Oscillatoriaceae	*Leptolyngbya*
				Leptolyngbya antarctica ANT.LG2.3	9.00E-169	98%	Cyanobacteria	Oscillatoriaceae	*Leptolyngbya*
				Leptolyngbya sp. CENA538	2.00E-155	95%	Cyanobacteria	Oscillatoriaceae	*Leptolyngbya*
Oligotype 10	GCCG	GGATCTGCCCTTAGGACGGGGACAACAGCTGGAACGGCTGCTAATACCCGATGTGCCGAGAGGTGAAACTATTTGGCCTGAGGATGAGCTCGCGTCTGATTAGCTAGTTGGAGTGGTAACGGCACACCAAGGCGACGATCAGTAGCTGGTCTGAGAGGATGATCAGCCACACTGGGACTGAGACACGGCCCAGACTCCTACGGGAGGCAGCAGTGGGAATTTCCGCAATGGGCGAAAGCCTGACGGAGCAAGACCGCGTGGGGGAAGAAGGCTCTTGGGTTGTAAACCCCTTTTCTCTGGGAAGAAAGTTGTGAAAGCAGCCTGACGGTACCAGAGGAATCAGCATCGGC	16	Uncultured Antarctic cyanobacterium	2.00E-145	94%	Cyanobacteria	-	-
				Oscillatoria amoena CCAP	9.00E-139	93%	Cyanobacteria	Oscillatoriaceae	*Oscillatoria*
				Microcoleus vaginatus	4.00E-137	92%	Cyanobacteria	Microcoleaceae	*Microcoleus*
Oligotype 11	CGCA	GGATCTGCCCTTAGGACGGGGACAACAGCTGGAACGGCTGCTAATACCCGATGTGCCGAGAGGTGAAACTATTTGGCCTGAGGATGAGCTCGCGTCTGATTAGCTAGTTGGAGTGGTAACGGCACACCAAGGCGACGATCAGTAGCTGGTCTGAGAGGATGATCAGCCACACTGGGACTGAGACACGGCCCAGACTCCTACGGGAGCAGCAGTGGGAATTTCCGCAATGGGCGAAAGCCTGACGGAGCAAGACCGCGTGGGGGA	11	Uncultured Antarctic cyanobacterium	9.00E-144	93%	Cyanobacteria		-
				Oscillatoria amoena CCAP	4.00E-137	92%	Cyanobacteria	Oscillatoriaceae	*Oscillatoria*
				Microcoleus vaginatus	2.00E-135	92%	Cyanobacteria	Microcoleaceae	*Microcoleus*

		AGAAGGCTCTTGGGTTGTAAACCCCTTTTCTCTGGGAAGAAAGTTGTGAAAGCAGCCTGACGGTACCAGAGGAATCAGCATCGGCT							
Oligotype 12	GAAC	TGGGGAATTTTCCGCAATGGGCGAAAGCCTGACGGAGCAAGACCGCGTGCGGGAGGAAGGTCTATTGATTGTAAACCGCTTTTGATAGGGAAGAAGATCTGACGGTACCTATCGAATCAGCATCGGCTAACTCCGTGCCAGCAGCCGCGGTAATACGGGGGATGCAAGCGTTATCCGGAATTATTGGGCGTAAAGCGTCCGTAGGTGGTTTTAAAAGTCTGTTGTCAAATCGCGAGGCTTAACCTCGTACAGGCAGTGGAAACTATAAGACTAGAGTGCGGTAGGGGTAGAGGGAATTTCCAGTGTAGCGGTGAAATGCGTAGATATTGGAAAGAACACCGGTGGCGAAG	10	Uncultured cyanobacterium clone	2.00E-170	100%	Cyanobacteria	-	-
				Uncultured Antarctic cyanobacterium	4.00E-157	100%	Cyanobacteria	-	-
				Leptolyngbya antarctica ANT.L18.1	9.00E-179	99%	Cyanobacteria	Oscillatoriaceae	*Leptolyngbya*
				Leptolyngbya antarctica ANT.L18.2	9.00E-179	99%	Cyanobacteria	Oscillatoriaceae	*Leptolyngbya*
				Leptolyngbya antarctica ANT.LWA.1	9.00E-179	99%	Cyanobacteria	Oscillatoriaceae	*Leptolyngbya*
				Leptolyngbya antarctica ANT.LG2.5	9.00E-179	99%	Cyanobacteria	Oscillatoriaceae	*Leptolyngbya*
				Leptolyngbya antarctica ANT.L67.1	9.00E-179	99%	Cyanobacteria	Oscillatoriaceae	*Leptolyngbya*
				Leptolyngbya antarctica ANT.LG2.3	9.00E-179	99%	Cyanobacteria	Oscillatoriaceae	*Leptolyngbya*
				Leptolyngbya antarctica ANT.LWAV6.1	6.00E-156	99%	Cyanobacteria	Oscillatoriaceae	*Leptolyngbya*
Oligotype 13	GGAA	GGATCTGCCCTTAGGACGGGGACAACAGCTGGAAACGGCTGCTAATACCCGATGTGCCGAGAGGTGAAACTATTTGGCCTGAGGATGAGCTCGCGTCTGATTAGCTAGTTGGAGTGGTAACGGCACACCAAGGCGACGATCAGTAGCTGGTCTGAGAGGATGATCAGCCACACTGGGACTGAGACACGGCCCAGACTCCTACGGGAGGCAGCAGTGGGGAATTTTCC	8	Uncultured cyanobacterium clone	7.00E-155	95%	Cyanobacteria	-	-
				Leptolyngbya antarctica ANT.L18.1	7.00E-145	94%	Cyanobacteria	Oscillatoriaceae	*Leptolyngbya*
				Leptolyngbya antarctica ANT.L18.2	7.00E-145	94%	Cyanobacteria	Oscillatoriaceae	*Leptolyngbya*
				Leptolyngbya antarctica ANT.LWA.1	7.00E-145	94%	Cyanobacteria	Oscillatoriaceae	*Leptolyngbya*

		GCAATGGGCGAAAGCCTGA CGGAGCAAGACCGCGTGG GGGAAGAAGGCTCTTGGGT TGTAAACCCCTTTTCTCTGG GAAGAACGCAATGACGGT ACCAGAGGAATCAGCATCG GCTAACTCCG		Leptolyngbya antarctica ANT.LG2.5	7.00E-145	94%	Cyanobacteria	Oscillatoriaceae	*Leptolyngbya*
				Leptolyngbya antarctica ANT.L67.1	7.00E-145	94%	Cyanobacteria	Oscillatoriaceae	*Leptolyngbya*
				Synechococcus sp. NgrSCC7	9.00E-144	94%	Cyanobacteria	Synechococcaceae	*Synechococcus*
Oligotype 14	GATC	GGATCTGCCCTTAGGACGG GGACGAACAGCTGGAAAC GGACTGCTAATAACCCGAC TGTGCCGAGAGGTGAAACT AATTTGGCCTGAGGATGAG CTCGCGTCTGATTAGCTAG TTGGAGTGGTAACGGCACA CCAAGGCGACGATCAGTAG CTGGTCTGAGAGGATGATC AGCCACACTGGGACTGAGA CACGGCCCAGACTCCTACG GGAGGCAGCAGTGGGGAA TTTTCCGCAATGGGCGAAA GCCTGACGGAGCAAGACCG CGTGCGGGAGGAAGGTCTA TTGATTGTAAACCGCTTTTG ATAGGGAAGAAGATCTGAC GGTACCTATCGAATCAGCA TCGGCTAAC	6	Uncultured cyanobacterium clone	4.00E-172	99%	Cyanobacteria	-	-
				Leptolyngbya antarctica ANT.L18.1	9.00E-169	98%	Cyanobacteria	Oscillatoriaceae	*Leptolyngbya*
				Leptolyngbya antarctica ANT.L18.2	9.00E-169	98%	Cyanobacteria	Oscillatoriaceae	*Leptolyngbya*
				Leptolyngbya antarctica ANT.LWA.1	9.00E-169	98%	Cyanobacteria	Oscillatoriaceae	*Leptolyngbya*
				Leptolyngbya antarctica ANT.LG2.5	9.00E-169	98%	Cyanobacteria	Oscillatoriaceae	*Leptolyngbya*
				Leptolyngbya antarctica ANT.L67.1	9.00E-169	98%	Cyanobacteria	Oscillatoriaceae	*Leptolyngbya*
				Leptolyngbya antarctica ANT.LG2.3	5.00E-166	98%	Cyanobacteria	Oscillatoriaceae	*Leptolyngbya*
				Leptolyngbya sp. CENA538	3.00E-153	95%	Cyanobacteria	Oscillatoriaceae	*Leptolyngbya*
				Leptolyngbya sp. O-77	7.00E-150	95%	Cyanobacteria	Oscillatoriaceae	*Leptolyngbya*
				Leptolyngbya frigida ANT.L52.2	9.00E-149	95%	Cyanobacteria	Oscillatoriaceae	*Leptolyngbya*
Oligotype 15	ACCA	GGATCTGCCCTTAGGACGG GGACGAACAGCTGGAAAC GGACTGCTAATAACCCGAC TGTGCCGAGAGGTGAAACT AATTTGGCCTGAGGATGAG CTCGCGTCTGATTAGCTAG TTGGAGTGGTAACGGCACA CCAAGGCGACGATCAGTAG	4	Uncultured cyanobacterium clone	5.00E-171	98%	Cyanobacteria	-	-
				Leptolyngbya antarctica ANT.L18.1	1.00E-167	98%	Cyanobacteria	Oscillatoriaceae	*Leptolyngbya*
				Leptolyngbya antarctica	1.00E-167	98%	Cyanobacteria	Oscillatoriaceae	*Leptolyngbya*

		CTGGTCTGAGAGGATGATC AGCCACACTGGGACTGAGA CACGGCCCAGACTCCTACG GGAGGCAGCAGTGGGAATT TTCCGCAATGGGCGAAAGC CTGACGGAGCAAGACCGCG TGCGGGAGGAAGGTCTATT GATTGTAAACCGCTTTTGA TAGGGAAGAAGATCTGACG GTACCTATCGAATCAGCAT CGGCTAACT		ANT.L18.2					
				Leptolyngbya antarctica ANT.LWA.1	1.00E-167	98%	Cyanobacteria	Oscillatoriaceae	*Leptolyngbya*
				Leptolyngbya antarctica ANT.LG2.5	1.00E-167	98%	Cyanobacteria	Oscillatoriaceae	*Leptolyngbya*
				Leptolyngbya antarctica ANT.L67.1	1.00E-167	98%	Cyanobacteria	Oscillatoriaceae	*Leptolyngbya*
				Leptolyngbya antarctica ANT.LG2.3	7.00E-165	98%	Cyanobacteria	Oscillatoriaceae	*Leptolyngbya*
				Leptolyngbya sp. CENA538	2.00E-151	95%	Cyanobacteria	Oscillatoriaceae	*Leptolyngbya*
				Leptolyngbya sp. O-77	3.00E-148	95%	Cyanobacteria	Oscillatoriaceae	*Leptolyngbya*
				Leptolyngbya frigida ANT.L52.2	1.00E-147	94%	Cyanobacteria	Oscillatoriaceae	*Leptolyngbya*

Supplementary Table 2(c)

**Oligotype ID**	**Oligotype Sequence**	**Representative Sequence**	**Abundance Oligotype**	**NCBI MEGABLAST Results**	**Average E value**	**Average Identity (%)**	**Phylum**	**Family**	**Genus**
Oligotype 1	TTC	TAGGGAATATTGGACAATG GGTGCGAGCCTGATCCAGC CATGCCGCGTGCAGGAAGA CGGCCTTCTGGGTTGTAAA CTGCTTTTACCAGGGGATA AAAAGACCATGCGTGGTCA ATTGAAGGTACCTGGTGAA TAAGCCACGGCTAACTACG TGCCAGCAGCCGCGGTAAT ACGTAGGTGGCAAGCGTTG TCCGGATTTATTGGGTTTA AAGGGTGCGTAGGCGGTTT TGTAAGTCAGTGGTGAAAT ACTCCAGCTCAACTGGAGA GGTGCCATTGATACTGCAG	99	Uncultured bacterium clone	2.00E-165	100%	-	-	-
				Uncultured Bacteroidetes bacterium clone	4.00E-142	96%	Bacteroidetes	-	-

		AACTTGAGTACAGACGAGGTAGGCGGAATTGACGG							
Oligotype 2	CCA	TAGGGAATATTGGACAATGGGTGCGAGCCTGATCCAGCCATGCCGCGTGCAGGAAGACGGCCTTCTGGGTTGTAAACTGCTTTTACCAGGGGATAAAAGACCATGCGTGGTCAATTGAAGGTACCTGGTGAATAAGCCACGGCTAACTACGTGCCAGCAGCCGCGGTAATACGTAGGTGGCAAGCGTTGTCCGGATTTATTGGGTTTAAAGGGTGCGTAGGCGGTTTTGTAAGTCAGTGGTGAAATACTCCAGCTCAACTGGAGAGGTGCCATTGATACTGCAGAACTTGAGTACAGACGAGGTAGGCGGAATTGACGGT	49	Uncultured bacterium clone	8.00E-164	99%	-	-	-
				Uncultured Bacteroidetes bacterium clone	4.00E-142	96%	Bacteroi detes	-	-
Oligotype 3	ATC	TAGGGAATATTGGACAATGGGTGAGAGCCTGATCCAGCCATGCCGCGTGCAGGAAGAAGGCCTTCTGGGTTGTAAACTGCTTTTGCCGGGGGATAAAAGATCCCTGCGGGGAGAATTGAAGGTACCCGGTGAATAAGCCACGGCTAACTACGTGCCAGCAGCCGCGGTAATACGTAGGTGGCAAGCGTTGTCCGGATTTATTGGGTTTAAAGGGTGCGTAGGCGGCCCTGTAAGTCAGTGGTGAAATACGGCAGCTCAACTGTCGAGGTGCCATTGATACTGCGGGGCTTGAGTACAGTCGAGGTAGGCGGAATTGACGG	40	Uncultured bacterium clone	8.00E-159	99%	-	-	-
Oligotype 4	TGT	TAGGGAATATTGGACAATGGGTGCGAGCCTGATCCAGCCATGCCGCGTGCAGGAAGACGGCCTTCTGGGTTGTAAACTGCTTTTACCAGGGGATAAAAAGACCATGCGTGGTCAATTGAAGGTACCTGGTGAATAAGCCACGGCTAACTACG	27	Uncultured bacterium clone	3.00E-163	99%	-	-	-
				Uncultured Bacteroidetes bacterium clone	7.00E-140	95%	Bacteroi detes	-	-

		TGCCAGCAGCCGCGGTAAT<br>ACGTAGGTGGCAAGCGTTG<br>TCCGGATTTTATTGGGTTTA<br>AAGGGTGCGTAGGCGGTTT<br>TGTAAGTCAGTGGTGAAAT<br>ACTCCAGCTCAACTGGAGA<br>GGTGCCATTGATACTGCAG<br>AACTTGAGTACAGACGAGG<br>TAGGCGGAATTGACG							
Oligotype 5	AAG	TAGGGAATATTGGACAATG<br>GGTGCGAGCCTGATCCAGC<br>CATGCCGCGTGCAGGAAGA<br>CGGCCTTCTGGGTTGTAAC<br>TGCTTTTACCAGGGGATAA<br>AAGACCATGCGTGGTCAAT<br>TGAAGGTACCTGGTGAATA<br>AGCCACGGCTAACTACGTG<br>CCAGCAGCCGCGGTAATAC<br>GTAGGTGGCAAGCGTTGTC<br>CGGATTTATTGGGTTTAAA<br>GGGTGCGTAGGCGGTTTTG<br>TAAGTCAGTGGTGAAATAC<br>TCCAGCTCAACTGGAGAGG<br>TGCCATTGATACTGCAGAA<br>CTTGAGTACAGACGAGGTA<br>GGCGGAATTGACGGTG	23	Uncultured bacterium clone	1.00E-162	99%	-	-	-
				Uncultured Bacteroidetes bacterium clone	5.00E-141	95%	Bacteroi<br>detes	-	-
Oligotype 6	GGT	TAGGGAATATTGGACAATG<br>GGTGCGAGCCTGATCCAGC<br>CATGCCGCGTGCAGGAAGA<br>CGGCCTTCTGGGTTGTAAA<br>CTGCTTTTACCAGGGGATA<br>AAAAGAACCATGCGTGGTC<br>AATTGAAGGTACCTGGTGA<br>ATAAGCCACGGCTAACTAC<br>GTGCCAGCAGCCGCGGTAA<br>TACGTAGGTGGCAAGCGTT<br>GTCCGGATTTATTGGGTTT<br>AAAGGGTGCGTAGGCGGTT<br>TTGTAAGTCAGTGGTGAAA<br>TACTCCAGCTCAACTGGAG<br>AGGTGCCATTGATACTGCA<br>GAACTTGAGTACAGACGAG<br>GTAGGCGGAATTGACG	21	Uncultured bacterium clone	3.00E-163	99%	-	-	-
				Uncultured Bacteroidetes bacterium clone	7.00E-140	95%	Bacteroi<br>detes	-	-
Oligotype 7	GAG	TAGGGAATATTGGACAATG	16	Uncultured	3.00E-158	99%	-	-	-

		GGTGCGAGCCTGATCCAGCCATGCCGCGTGCAGGAAGACGGCCTTCTGGGTTGTAAACTGCTTTTACCTAGGGGAGTAAAAGAACCATGCGTGGTCAATTGAAGGTACCTGGTGAATAAGCCACGGCTAACTACGTGCCAGCAGCCGCGGTAATACGTAGGTGGCAAGCGTTGTCCGGATTTATTGGGTTTAAAGGGTGCGTAGGCGGTTTTGTAAGTCAGTGGTGAAATACTCCAGCTCAACTGGAGAGGTGCCATTGATACTGCAGAACTTGAGTACAGACGAGGTAGGCGGAATTGAC		bacterium clone					
				Uncultured Bacteroidetes bacterium clone	1.00E-136	95%	Bacteroi detes	-	-
Oligotype 8	CAG	TAGGGAATATTGGACAATGGGTGCGAGCCTGATCCAGCCATGCCGCGTGCAGGAAGACGGCCTTCTGGGTTGTAAACTGCTTTTACCAGGGGATAAAAGACCATGCGTGGTCAATTGAAGGTACCTGGTGAATAAGCCACGGCTAACTACGTGCCAGCAGCCGCGGTAATACGTAGGTGGCAAGCGTTGTCCGGATTTATTGGGTTAAAGGGTGCGTAGGCGGTTTTGTAAGTCAGTGGTGAAATACTCCAGCTCAACTGGAGAGGTGCCATTGATACTGCAGAACTTGAGTACAGACGAGGTAGGCGGAATTGACGGTG	15	Uncultured bacterium clone	1.00E-162	99%	-	-	-
				Uncultured Bacteroidetes bacterium clone	5.00E-141	95%	Bacteroi detes	-	-
Oligotype 9	TCA	TAGGGAATATTGGACAATGGGTGCGAGCCTGATCCAGCCATGCCGCGTGCAGGAAGACGGCCTTCTGGGTTGTAAACTGCTTTTACCAGGGGATAAAAAGACCATGCGTGGTCAATTGAAGGTACCTGGTGAATAAGCCACGGCTAACTACGTGCCAGCAGCCGCGGTAATACGTAGGTGGCAAGCGTTGTCCGGATTTATTGGGTTTAAAGGTGCGTAGGCGGTTTT	11	Uncultured bacterium clone	8.00E-164	99%	-	-	-
				Uncultured Bacteroidetes bacterium clone	2.00E-140	95%	Bacteroi detes	-	-

		GTAAGTCAGTGGTGAAATACTCCAGCTCAACTGGAGAGGTGCCATTGATACTGCAGAACTTGAGTACAGACGAGGTAGGCGGAATTGACGGT							
Oligotype 10	ACA	TAGGGAATATTGGACAATGGGTGAGAGCCTGATCCAGCCATGCCGCGTGCAGGAAGAAGGCCTTCTGGGTTGTAAACTGCTTTTGCCGGGGGATAAAAGATCCCTGCGGGGAGAATTGAAGGTACCCGGTGAATAAGCCACGGCTAACTACGTGCCAGCAGCCGCGGTAATACGTAGGTGGCAAGCGTTGTCCGGATTTATTGGGTTTAAAGGTGCGTAGGCGGCCCTGTAAGTCAGTGGTGAAATACGGCAGCTCAACTGTCGAGGTGCCATTGATACTGCGGGGCTTGAGTACAGTCGAGGTAGGCGGAATTGACGGT	9	Uncultured bacterium clone	4.00E-157	98%	-	-	-
Oligotype 11	CTC	TAGGGAATATTGGTCAATGGACGAGAGTCTGAACCAGCCATGCCGCGTGCAGGAAGAAGGCCTTCTGGGTTGTAAACTGCTTTTGACAGGGAGCAAAATGGCTATGCGTAGCAAATTGAGAGTACCTGTAGAATAAGCACCGGCTAACTCCGTGCCAGCAGCCGCGGTAATACGGAGGGTGCAAGCGTTGTCCGGATTTATTGGGTTTAAAGGGTGCGTAGGCGGCTATATAAGTCAGTGGTGAAATACGACAGCTCAACTGTCGAACTGCCATTGATACTGTATGGCTTGAGTACAGACGAGGTAGGCGGAATTGATAG	8	Uncultured bacterium clone	1.00E-142	96%	-	-	-
				Uncultured Cytophaga sp.	2.00E-130	93%	Bacteroidetes	Cytophagaceae	Cytophaga
				Flexibacter aggregans	1.00E-127	93%	Bacteroidetes	Flexibacteraceae	Flexibacter
Oligotype 12	ATG	TAGGGAATATTGGACAATGGGTGCGAGCCTGATCCAGCCATGCCGCGTGCAGGAAGACGGCCTTCTGGGTTGTAACTGCTTTTACCAGGGATAAA	2	Uncultured bacterium clone	6.00E-160	99%	-	-	-
				Uncultured Bacteroidetes bacterium clone	3.00E-138	95%	Bacteroidetes	-	-

		AGACCATGCGTGGTCAATT GAGGTACCTGGTGAATAAG CCACGGCTAACTACGTGCC AGCAGCCGCGGTAATACGT AGGTGGCAAGCGTTGTCCG GATTTATTGGGTTTAAAGG GTGCGTAGGCGGTTTTGTA AGTCAGTGGTGAAATACTC CAGCTCAACTGGAGAGGTG CCATTGATACTGCAGAACT TGAGTACAGACGAGGTAGG CGGAATTGACGGTGTA							

Supplementary Table 2(d)

Oligotype ID	Oligotype Sequence	Representative Sequence	Abundance	NCBI MEGABLAST Results	Average E value	Average Identity (%)	Phylum	Family	Genus
Oligotype 1	CGT	ACAATTTACCTTCAAATGG GGAATAGCTCGGCGAAAGC CGGATTAATACCGCATGTG GTTGGCAGCTGCATGGTTG CCATACTAAAGTCGGGGAC CGCAAGGCCTGACGTTTGA AGAGGAGTTCGCGGCCTAT CAGCTAGTTGGCGGGGTAA CGGCCCACCAAGGCTAAGA CGGGTAGCTGGTCTGAGAG GATGATCAGCCACACTGGA ACTGAGACACGGTCCAGAC ACCTACGGGTGGCAGCAGT TTCGAATCATTCACAATGG GCGAAAGCCTGATGGTGCG ACGCCGCGTGAGGGATGAA GGTCTTCGGATTGTAA	275	Uncultured bacterium clone	2.00E-165	100%	-	-	-
				Uncultured Opitutus sp.	8.00E-164	99%	Verruco microbia	Opitutaceae	Opitutus
				Opitutus sp. VeGlc2	2.00E-140	95%	Verruco microbia	Opitutaceae	Opitutus
				Opitutus sp. VeCb1	9.00E-139	95%	Verruco microbia	Opitutaceae	Opitutus
				Opitutus terrae	4.00E-137	95%	Verruco microbia	Opitutaceae	Opitutus
				Uncultured Verrucomicrobia bacterium clone	2.00E-135	94%	Verruco microbia	-	-
Oligotype 2	TAT	ACAATTTACCTTCAAATGG GGAATAGCTCGGCGAAAGC CGGATTAATACCGCATGTG GTTGGCAATCACATGATTG CCATACTAAAGTCGGGGAC CGCAAGGCCTGACGTTTGA AGAGGAGTTCGCGGCCTAT	215	Uncultured bacterium clone	4.00E-157	98%	-	-	-
				Uncultured Opitutus sp.	2.00E-155	98%	Verruco microbia	Opitutaceae	Opitutus
				Uncultured Verrucomicrobia bacterium clone	9.00E-139	95%	Verruco microbia	-	-

		CAGCTAGTTGGCGGGGTAA<br>CGGCCCACCAAGGCTAAGA<br>CGGGTAGCTGGTCTGAGAG<br>GATGATCAGCCACACTGGA<br>ACTGAGACACGGTCCAGAC<br>ACCTACGGGTGGCAGCAGT<br>TTCGAATCATTCACAATGG<br>GCGAAAGCCTGATGGTGCG<br>ACGCCGCGTGAGGGATGAA<br>GGTCTTCGGATTGTAA		Opitutus terrae	4.00E-137	95%	Verruco microbia	Opitutaceae	Opitutus
				Verrucomicrobia bacterium pACH90	2.00E-135	94%	Verruco microbia	-	-
				Opitutus sp. VeCb1	2.00E-135	94%	Verruco microbia	Opitutaceae	Opitutus
Oligotype 3	ACG	ACAATTTACCTTCAAATAG<br>GGGAAGTAGCTCGGCGAA<br>AGCCGGATTAATACCGCAT<br>GTGGTTGGCAGCTGCATGG<br>TTGCCATACTAAAGTCGGG<br>GACCGCAAGGCCTGACGTT<br>TGAAGAGGAGTTCGCGGCC<br>TATCAGCTAGTTGGCGGGG<br>TAACGGCCCACCAAGGCTA<br>AGACGGGTAGCTGGTCTGA<br>GAGGATGATCAGCCACACT<br>GGAACTGAGACACGGTCCA<br>GACACCTACGGGTGGCAGC<br>AGTTTCGAATCATTCACAA<br>TGGGCGAAAGCCTGATGGT<br>GCGACGCCGCGTGAGGGAT<br>GAAGGTCTTCGGATTGT	97	Uncultured bacterium clone	1.00E-161	99%	-	-	-
				Uncultured Opitutus sp.	6.00E-160	99%	Verruco microbia	Opitutaceae	Opitutus
				Opitutus sp. VeGlc2	1.00E-136	95%	Verruco microbia	Opitutaceae	Opitutus
				Opitutus sp. VeCb1	7.00E-135	94%	Verruco microbia	Opitutaceae	Opitutus
				Opitutus terrae	3.00E-133	94%	Verruco microbia	Opitutaceae	Opitutus
Oligotype 4	GTC	ACAATTTACCTTCAAATGG<br>GGAAGTAGCTCGGCGAAA<br>GCCGGATTAATACCGCATG<br>TGGTTGGCAGCTGCATGGT<br>TGCCATACTAAAGTCGGGG<br>ACCGCAAGGCCTGACGTTT<br>GAAGAGGAGTTCGCGGCCT<br>ATCAGCTAGTTGGCGGGGT<br>AACGGCCCACCAAGGCTAA<br>GACGGGTAGCTGGTCTGAG<br>AGGATGATCAGCCACACTG<br>GAACTGAGACACGGTCCAG<br>ACACCTACGGGTGGCAGCA<br>GTTTCGAATCATTCACAAT<br>GGGCGAAAGCCTGATGGTG<br>CGACGCCGCGTGAGGGATG<br>AAGGTCTTCGGATTGTA	94	Uncultured bacterium clone	3.00E-163	99%	-	-	-
				Uncultured Opitutus sp.	1.00E-161	99%	Verruco microbia	Opitutaceae	Opitutus
				Opitutus sp. VeGlc2	3.00E-138	95%	Verruco microbia	Opitutaceae	Opitutus
				Opitutus sp. VeCb1	1.00E-136	95%	Verruco microbia	Opitutaceae	Opitutus
				Opitutus terrae	7.00E-135	94%	Verruco microbia	Opitutaceae	Opitutus

Oligotype 5	TCA	ACAATTTACCTTCAAATGGGGAATAGCTCGGCGAAGCCGGATTAATACCGCATGTGGTTGGCAGCTGCATGGTTGCCATACTAAAGTCGGGGACCGCAAGGCCTGACGTTTGAAGAGGAGTTCGCGGCCTATCAGCTAGTTGGCGGGGTAACGGCCCACCAAGGCTAAGACGGGTAGCTGGTCTGAGAGGATGATCAGCCACACTGGAACTGAGACACGGTCCAGACACCTACGGGTGGCAGCAGTTTCGAATCATTCACAATGGGCGAAAGCCTGATGGTGCGACGCCGCGTGAGGGATGAAGGTCTTCGGATTGTAAA	76	Uncultured bacterium clone	8.00E-164	99%	-	-	-
				Uncultured Opitutus sp.	4.00E-162	99%	Verruco microbia	Opitutaceae	Opitutus
				Opitutus sp. VeGlc2	9.00E-139	95%	Verruco microbia	Opitutaceae	Opitutus
				Opitutus sp. VeCb1	4.00E-137	95%	Verruco microbia	Opitutaceae	Opitutus
				Opitutus terrae	2.00E-135	94%	Verruco microbia	Opitutaceae	Opitutus
Oligotype 6	ACC	ACAATTTACCTTCAAATGGGGAAGTAGCTCGGCGAAAGCCGGATTAATACCGCATGTGGTTGGCAATCACATGATTGCCATACTAAAGTCGGGGACCGCAAGGCCTGACGTTTGAAGAGGAGTTCGCGGCCTATCAGCTAGTTGGCGGGGTAACGGCCCACCAAGGCTAAGACGGGTAGCTGGTCTGAGAGGATGATCAGCCACACTGGAACTGAGACACGGTCCAGACACCTACGGGTGGCAGCAGTTTCGAATCATTCACAATGGGCGAAAGCCTGATGGTGCGACGCCGCGTGAGGGATGAAGGTCTTCGGATTGTA	71	Uncultured bacterium clone	6.00E-155	98%	-	-	-
				Uncultured Opitutus sp.	3.00E-153	98%	Verruco microbia	Opitutaceae	Opitutus
				Opitutus terrae	7.00E-135	94%	Verruco microbia	Opitutaceae	Opitutus
				Verrucomicrobiae bacterium pACH90	3.00E-133	94%	Verruco microbia	-	-
				Opitutus sp. VeCb1	3.00E-133	94%	Verruco microbia	Opitutaceae	Opitutus
Oligotype 7	GAA	ACAATTTTACCTTCAAATAGGGGAAGTAGCTCGGCGAAAGACCGGATTAATACCGCATGTGGTTGGCAGCTGCATGGTTGCCATACTAAAGTCGGGGACCGCAAGGCCTGACGTTTGAAGAGGAGTTCGCGGCCTATCAGCTAGTTGGCGGGGTAACGGCCCACCAAGGCTAAGACGGGTAGCTGGTCT	61	Uncultured bacterium clone	1.00E-157	99%	-	-	-
				Uncultured Opitutus sp.	6.00E-155	99%	Verruco microbia	Opitutaceae	Opitutus
				Uncultured Verrucomicrobia bacterium clone	2.00E-129	95%	Verruco microbia	-	-
				Uncultured Opitutaceae bacterium clone	1.00E-127	95%	Verruco microbia	Opitutaceae	-

		GAGAGGATGATCAGCCACA CTGGAACTGAGACACGGTC CAGACACCTACGGGTGGCA GCAGTTTCGAATCATTCAC AATGGGCGAAAGCCTGATG GTGCGACGCCGCGTGAGGG ATGAAGGTCTTCGGATT		Opitutus sp. VeGlc2	1.00E-132	94%	Verruco microbia	Opitutaceae	Opitutus
				Opitutus sp. VeCb1	5.00E-131	94%	Verruco microbia	Opitutaceae	Opitutus
				Opitutus terrae	2.00E-129	93%	Verruco microbia	Opitutaceae	Opitutus
Oligotype 8	ATG	ACAATTTACCTTCAAATAG GGGAAGTAGCTCGGCGAA AGCCGGATTAATACCGCAT GTGGTTGGCAATCACATGA TTGCCATACTAAAGTCGGG GACCGCAAGGCCTGACGTT TGAAGAGGAGTTCGCGGCC TATCAGCTAGTTGGCGGGG TAACGGCCCACCAAGGCTA AGACGGGTAGCTGGTCTGA GAGGATGATCAGCCACACT GGAACTGAGACACGGTCCA GACACCTACGGGTGGCAGC AGTTTCGAATCATTCACAA TGGGCGAAAGCCTGATGGT GCGACGCCGCGTGAGGGAT GAAGGTCTTCGGATTGT	55	Uncultured bacterium clone	3.00E-153	98%	-	-	-
				Uncultured Opitutus sp.	1.00E-151	98%	Verruco microbia	Opitutaceae	Opitutus
				Uncultured Verrucomicrobia bacterium clone	7.00E-135	94%	Verruco microbia	-	-
				Opitutus terrae	3.00E-133	94%	Verruco microbia	Opitutaceae	Opitutus
				Verrucomicrobiae bacterium pACH90	1.00E-131	94%	Verruco microbia	Opitutaceae	Opitutus
Oligotype 9	GAC	TTTCGAATCATTCACAATG GGCGAAAGCCTGATGGTGC GACGCCGCGTGAGGGATGA AGGTCTTCGGATTGTAAAC CTCTGTCACCGGGGAAGAA ACGCTTTGAATTAACAGTC CAAAGCCTGACTTAACCCG GAGAGGAAGCAGTGGCTA ACTCTGTGCCAGCAGCCGC GGTAATACAGAGACTGCAA GCGTTATTCGGATTCACTG GGCGTAAAGGGTGCGCAG GCGGCCGGATGTGTCAGGT GTGAAAGCCCGGGGCTTAA CCCCGGAATTGCGCCTGAA ACTGTCCGGCTAGAGTACT GGAGAGGGTAGCGGAATT	47	Uncultured bacterium clone	4.00E-152	98%	-	-	-
				Uncultured Opitutus sp.	4.00E-152	98%	Verruco microbia	Opitutaceae	Opitutus
				Opitutus sp. VeSm13	9.00E-139	95%	Verruco microbia	Opitutaceae	Opitutus
				Verrucomicrobiae bacterium pACH90	4.00E-137	95%	Verruco microbia	Opitutaceae	Opitutus
				Opitutus sp. NVT1	9.00E-134	94%	Verruco microbia	Opitutaceae	Opitutus
Oligotype 10	CGG	ACAATTTACCTTCAAATAG GGGAAGTAGCTCGGCGAA AGACCGGATTAATACCGCA	38	Uncultured bacterium clone	2.00E-159	99%	-	-	-
				Uncultured	1.00E-157	99%	Verruco	Opitutaceae	Opitutus

		TGTGGTTGGCAGCTGCATG<br>GTTGCCATACTAAAGTCGG<br>GGACCGCAAGGCCTGACGT<br>TTGAAGAGGAGTTCGCGGC<br>CTATCAGCTAGTTGGCGGG<br>GTAACGGCCCACCAAGGCT<br>AAGACGGGTAGCTGGTCTG<br>AGAGGATGATCAGCCACAC<br>TGGAACTGAGACACGGTCC<br>AGACACCTACGGGTGGCAG<br>CAGTTTCGAATCATTCACA<br>ATGGGCGAAAGCCTGATGG<br>TGCGACGCCGCGTGAGGGA<br>TGAAGGTCTTCGGATTG		Opitutus sp.			microbia		
				Opitutus sp. VeGlc2	2.00E-134	94%	Verruco microbia	Opitutaceae	Opitutus
				Opitutus sp. VeCb1	1.00E-132	94%	Verruco microbia	Opitutaceae	Opitutus
				Opitutus terrae	5.00E-131	94%	Verruco microbia	Opitutaceae	Opitutus
Oligotype 11	CCA	ACAATTTACCTTCAAATGG<br>GGAATAGCTCGGCGAAGCC<br>GGATTAATACCGCATGTGG<br>TTGGCAATCACATGATTGC<br>CATACTAAAGTCGGGGACC<br>GCAAGGCCTGACGTTTGAA<br>GAGGAGTTCGCGGCCTATC<br>AGCTAGTTGGCGGGGTAAC<br>GGCCCACCAAGGCTAAGAC<br>GGGTAGCTGGTCTGAGAGG<br>ATGATCAGCCACACTGGAA<br>CTGAGACACGGTCCAGACA<br>CCTACGGGTGGCAGCAGTT<br>TCGAATCATTCACAATGGG<br>CGAAAGCCTGATGGTGCGA<br>CGCCGCGTGAGGGATGAAG<br>GTCTTCGGATTGTAAA	34	Uncultured bacterium clone	2.00E-155	98%	-	-	-
				Uncultured Opitutus sp.	8.00E-154	98%	Verruco microbia	Opitutaceae	Opitutus
				Uncultured Verrucomicrobia bacterium clone	4.00E-137	95%	Verruco microbia	-	-
				Opitutus terrae	2.00E-135	94%	Verruco microbia	Opitutaceae	Opitutus
				Verrucomicrobiae bacterium pACH90	9.00E-134	94%	Verruco microbia	Opitutaceae	Opitutus
				Opitutus sp. VeCb1	9.00E-134	94%	Verruco microbia	Opitutaceae	Opitutus
Oligotype 12	CGC	ACAATTTACCTTCAAATGG<br>GGAATAGCTCGGCGAAAGC<br>CGGATTAATACCGCATGTG<br>GTTGGCAGCTGCATGGTTG<br>CCATACTAAAAGTCGGGGA<br>CCGCAAGGCCTGACGTTTG<br>AAGAGGAGTTCGCGGCCTA<br>TCAGCTAGTTGGCGGGGTA<br>ACGGCCCACCAAGGCTAAG<br>ACGGGTAGCTGGTCTGAGA<br>GGATGATCAGCCACACTGG<br>AACTGAGACACGGTCCAGA<br>CACCTACGGGTGGCAGCAG<br>TTTCGAATCATTCACAATG	33	Uncultured bacterium clone	3.00E-163	99%	-	-	-
				Uncultured Opitutus sp.	1.00E-161	99%	Verruco microbia	Opitutaceae	Opitutus
				Opitutus sp. VeGlc2	3.00E-138	95%	Verruco microbia	Opitutaceae	Opitutus
				Opitutus sp. VeCb1	1.00E-136	95%	Verruco microbia	Opitutaceae	Opitutus
				Opitutus terrae	7.00E-135	94%	Verruco microbia	Opitutaceae	Opitutus

		GGCGAAAGCCTGATGGTGC GACGCCGCGTGAGGGATGA AGGTCTTCGGATTGTA							
Oligotype 13	TAC	ACAATTTACCTTCAAATGG GGAATAGCTCGGCGAAAGC CGGATTAATACCGCATGTG GTTGGCAATCACATGATTG CCATACTAAAAGTCGGGGA CCGCAAGGCCTGACGTTTG AAGAGGAGTTCGCGGCCTA TCAGCTAGTTGGCGGGGTA ACGGCCCACCAAGGCTAAG ACGGGTAGCTGGTCTGAGA GGATGATCAGCCACACTGG AACTGAGACACGGTCCAGA CACCTACGGGTGGCAGCAG TTTCGAATCATTCACAATG GGCGAAAGCCTGATGGTGC GACGCCGCGTGAGGGATGA AGGTCTTCGGATTGTA	24	Uncultured bacterium clone	6.00E-155	98%	-	-	-
				Uncultured Opitutus sp.	3.00E-153	98%	Verruco microbia	Opitutaceae	Opitutus
				Uncultured Verrucomicrobia bacterium clone	1.00E-136	95%	Verruco microbia	-	-
				Opitutus terrae	7.00E-135	94%	Verruco microbia	Opitutaceae	Opitutus
				Verrucomicrobiae bacterium pACH90	3.00E-133	94%	Verruco microbia	Opitutaceae	Opitutus
				Opitutus sp. VeCb1	3.00E-133	94%	Verruco microbia	Opitutaceae	Opitutus
Oligotype 14	TGT	ACAATTCACCGACAAGTGT GGAATAGCTCGTCGAAAGA CGAGATAATACCGCATATG GTCGTTCATCGCATGATGG ACGAACTAAAGTTAGGGAC CGTAAGGCCTGACGCTAGT CGAGAAGTTCGCGGCCTAT CAGCTAGTTGGCGAGGTAA CGGCTCACCAAGGCTAAGA CGGGTAGCTGATCTGAGAG GATGATCAGCCACACTGGA ACTGAGACACGGTCCAGAC ACCTACGGGTGGCAGCAGT TTCGAATCATTCACAATGG GCGAAAGCCTGATGGTGCG ACGCCGCGTGAGGGATGAA GGTCTTCGGATTGTAA	12	Uncultured bacterium clone	2.00E-129	94%	-	-	-
				Uncultured Opitutus sp.	1.00E-127	94%	Verruco microbia	Opitutaceae	Opitutus
				Uncultured Verrucomicrobia bacterium clone	5.00E-126	94%	Verruco microbia	-	-

Supplementary Table 2(e)

Oligotype ID	Oligotype Sequence	Representative Sequence	Abundance Ol	NCBI MEGABLAST Results	Average E value	Average Identity (%)	Phylum	Family	Genus
Oligotype 1	TCAAACACTCCTGCAGACTCACTAG	TGGGGAATATTGGACAATGGGCGCAAGCCTGATCCAGCCATACCGCGTGGGTGAAGAAGGCCTTCGGGTTGTAAAGCCCTTTTGTTGGGAAAGAAATCCTGCAGGTTAATAACCAGTAGGGATGACGGTACCCAAAGAATAAGCACCGGCTAACTTCGTGCCAGCAGCCGCGGTAATACGAAGGGTGCAAGCGTTACTCGGAATTACTGGGCGTAAAGCGTGCGTAGGTGGTTGTTTAAGTCTGCTGTGAAAGCCCCGGGCTCAACCTGGGAATTGCAGTGGATACTGGGCGACTAGAGT	31	Uncultured bacterium clone	5.00E-151	99%	-	-	-
				Pseudoxanthomonas sp.	5.00E-151	99%	Proteobacteria	Xanthomonadaceae	Pseudoxanthomonas
				Pseudomonas sp.	5.00E-151	99%	Proteobacteria	Pseudomonadaceae	Pseudomonas
				Xanthomonas sp.	5.00E-151	99%	Proteobacteria	Xanthomonadaceae	Xanthomonas
				Stenotrophomonas sp.	1.00E-147	99%	Proteobacteria	Xanthomonadaceae	Stenotrophomonas
Oligotype 2	TCAAACACTCTCTGCATACGGACTA	GGGGGAATATTGGACAATGGGCGCAAGCCTGATCCAGCCATACCGCGTGGGTGAAGAAGGCCTTCGGGTTGTAAAGCCCTTTTGTTGGGAAAGAAATCCTGCAGGTTAATAACCAGTAGGGATGACGGTACCCAAAGAATAAGCACCGGCTAACTTCGTGCCAGCAGCCGCGGTAATACGAAGGGTGCAAGCGTTACTCGGAATTACTGGGCGTAAAGCGTGCGTAGGTGGTTGTTTAAGTCTGCTGTGAAAGCCCCCGGGCTCAACCTGGGAATTGCAGTGGATACTGGGCGACTAGAG	15	Uncultured bacterium clone	8.00E-149	99%	-	-	-
				Pseudoxanthomonas sp.	3.00E-148	99%	Proteobacteria	Xanthomonadaceae	Pseudoxanthomonas
				Pseudomonas sp.	3.00E-148	99%	Proteobacteria	Pseudomonadaceae	Pseudomonas
				Xanthomonas sp.	3.00E-148	99%	Proteobacteria	Xanthomonadaceae	Xanthomonas
				Pseudoxanthomonas ginsengisoli	3.00E-148	99%	Proteobacteria	Xanthomonadaceae	Pseudoxanthomonas
				Stenotrophomonas sp.	6.00E-145	98%	Proteobacteria	Xanthomonadaceae	Stenotrophomonas
Oligotype 3	GAGGTTCTCTAGCAGTCTGGCTAGT	TGGGGGAATATTGGACAATGGGCGCAAGCCTGATCCAGCCATACCGCGTGGGTGAAGAAGGCCTCGGGTTGTAAGCCCTTTTGTTGGGTAAGAAATCCTGCAGGTTAATAACCA	11	Uncultured bacterium clone	1.00E-146	98%	-	-	-
				Pseudoxanthomonas sp.	2.00E-145	98%	Proteobacteria	Xanthomonadaceae	Pseudoxanthomonas
				Pseudomonas sp.	2.00E-145	98%	Proteobacteria	Pseudomonadaceae	Pseudomonas

		GTAGGGATGACGGTACCCAAAGAATAAGCACCGGCTAACTTCGTGCCAGCAGCCGCGGTAATACGAAGGGTGCAAGCGTTACTCGGAATTACTGGGCGTAAAGCGTGCGTAGGTGGTTGTTTAAGTCTGCTGTGAAAGCCCCGGGCTCAACCTGGGAATTGCAGTGGATACTGGGCGACTAGAGTG		Xanthomonas sp.	2.00E-145	98%	Proteobacteria	Xanthomonadaceae	Xanthomonas
				Pseudoxanthomonas ginsengisoli	2.00E-145	98%	Proteobacteria	Xanthomonadaceae	Pseudoxanthomonas
				Stenotrophomonas sp.	4.00E-142	98%	Proteobacteria	Xanthomonadaceae	Stenotrophomonas
Oligotype 4	GTTCCAACTTCTGCAAACTTGCTAG	TGGGGAATATTGGACAATGGGGGCAACCCTGATCCAGCAATGCCGCGTGTGTGAAGAAGGCCTTCGGGTTGTAAAGCACTTTTATTGGGAACGAAAAGCGATGGGTTAATACCTTATCGTGCTGACGGTACCCAAGGAATAAGCACCGGCTAACTTCGTGCCAGCAGCCGCGGTAATACGAAGGGTGCAAGCGTTAATCGGAATTACTGGGCGTAAAGGGTGCGTAGGCGGTTATTTAAGTCTGTTGTGAAAGCCCTGGGCTCAACCTGGGAATGGCAATGGATACTGGGTAGCTAGAGT	10	Uncultured bacterium clone	4.00E-132	100%	-	-	-
				Uncultured Rhodanobacter sp.	5.00E-126	94%	Proteobacteria	Xanthomonadaceae	Rhodanobacter
				Rhodanobacter sp.	2.00E-124	94%	Proteobacteria	Xanthomonadaceae	Rhodanobacter
				Rhodanobacter ginsengisoli	2.00E-124	94%	Proteobacteria	Xanthomonadaceae	Rhodanobacter
				Dyella sp.	1.00E-122	94%	Proteobacteria	Xanthomonadaceae	Dyella
				Xanthomonadaceae bacterium	1.00E-122	94%	Proteobacteria	Xanthomonadaceae	-
Oligotype 5	CGCTGATAGGTCTGCATACGGACTA	GTGGGGAATATTGGACAATGGGCGCAAGCCTGATCCAGCCATACCGCGTGGGTGAAGAAGGCCTTCGGGTTGTAAAGCCCTTTTGTTGGGAAAGAAATCCTGCAGGTTAATAACCAGTAGGGATGACGGTACCCAAAGAATAAGCACCGGCTAACTTCGTGCCAGCAGCCGCGGTAATACGAAGGGTGCAAGCGTTACTCGGAATTACTGGGCGTAAAGCGTGCGTAGGTGGTTGTTTAAGTCTGCTGTGAAAGCCCCGGGCTCAACCTGGGAATTGCAGTGGATACTGGGCGACTAGAG	5	Uncultured bacterium clone	5.00E-151	99%	-	-	-
				Pseudoxanthomonas sp.	5.00E-151	99%	Proteobacteria	Xanthomonadaceae	Pseudoxanthomonas
				Pseudomonas sp.	5.00E-151	99%	Proteobacteria	Pseudomonadaceae	Pseudomonas
				Xanthomonadaceae bacterium	5.00E-151	99%	Proteobacteria	Xanthomonadaceae	-
				Xanthomonas sp.	5.00E-151	99%	Proteobacteria	Xanthomonadaceae	Xanthomonas
				Stenotrophomonas sp.	1.00E-147	99%	Proteobacteria	Xanthomonadaceae	Stenotrophomonas
Oligotype 6	GAGGTTCTCTCTGCA	TGGGGAATATTGGACAATGGGCGCAAGCCTGATCCAGC	4	Uncultured bacterium clone	1.00E-147	99%	-	-	-

	GACTCACTAG	CATACCGCGTGGGTGAAGAAGGCCTTCGGGTTGTAAAGCCCTTTTGTTGGGAAGAAATCCTGCAGGTTAATAACCAGTAGGGATGACGGTACCCAAAGAATAAGCACCGGCTAACTTCGTGCCAGCAGCCGCGGTAATACGAAGGGTGCAAGCGTTACTCGGAATTACTGGGCGTAAAGCGTGCGTAGGTGGTTGTTTAAGTCTGCTGTGAAAGCCCCCGGGCTCAACCTGGGAATTGCAGTGGATACTGGGCGACTAGAGT		Pseudoxanthomonas sp.	1.00E-147	99%	Proteobacteria	Xanthomonadaceae	Pseudoxanthomonas
				Pseudomonas sp.	1.00E-147	99%	Proteobacteria	Pseudomonadaceae	Pseudomonas
				Xanthomonadaceae bacterium	1.00E-147	99%	Proteobacteria	Xanthomonadaceae	-
				Xanthomonas sp.	1.00E-147	99%	Proteobacteria	Xanthomonadaceae	Xanthomonas
				Stenotrophomonas sp.	2.00E-144	98%	Proteobacteria	Xanthomonadaceae	Stenotrophomonas
Oligotype 7	CGTGGCTGTGAGAGTGTGGATAGAG	TGGGGAATATTGGACAATGGGCGCAAGCCTGATCCAGCCATACCGCGTGGGTGAAGAAGGCCTTCGGGTTGTAAAGCCCTTTGTTGGGAAGAAATCCTGCAGGTTAATAACCAGTAGGGATGACGGTACCCAAAGAATAAGCACCGGCTAACTTCGTGCCAGCAGCCGCGGTAATACGAAGGGTGCAAGCGTTACTCGGAATTACTGGGCGTAAAGCGTGCGTAGGTGGTTGTTTAAGTCTGCTGTGAAAGCCCCGGGCTCAACCTGGGAATTGCAGTGGATACTGGGCGACTAGAGTGT	4	Uncultured bacterium clone	3.00E-148	99%	-	-	-
				Pseudoxanthomonas sp.	3.00E-148	99%	Proteobacteria	Xanthomonadaceae	Pseudoxanthomonas
				Pseudomonas sp.	3.00E-148	99%	Proteobacteria	Pseudomonadaceae	Pseudomonas
				Xanthomonadaceae bacterium	3.00E-148	99%	Proteobacteria	Xanthomonadaceae	-
				Xanthomonas sp.	3.00E-148	99%	Proteobacteria	Xanthomonadaceae	Xanthomonas
				Xanthomonadaceae bacterium	3.00E-148	99%	Proteobacteria	Xanthomonadaceae	-
				Stenotrophomonas sp.	6.00E-145	98%	Proteobacteria	Xanthomonadaceae	Stenotrophomonas
Oligotype 8	GTTTCCACTACTGCACACTGGCTAG	TAGGGAATATTGGACAATGGGCGCAAGCCTGATCCAGCTATGCCGCGTGTGTGAAGAAGGCCTTCGGGTTGTAAAGCACTTTTGTCCGGAAAGAAAAGCGCTGGGTTAACACCCTGGTGTCCTGACGGTACCGGAAGAATAAGCACCGGCTAACTTCGTGCCAGCAGCCGCGGTAATACGAAGGGTGCAAGCGTTACTCGGAATTACTGGGCGTAAAGCGTGCGTAGGTGGTTTGTTAAGTCTGATGTGAAAGCCCTGGGCTCAACCTGGGAATGGCACTGGATAC	3	Uncultured Thermomonas sp.	2.00E-140	99%	Proteobacteria	Xanthomonadaceae	Thermomonas
				Uncultured bacterium clone	2.00E-149	99%	-	-	-
				Thermomonas sp.	1.00E-142	98%	Proteobacteria	Xanthomonadaceae	Thermomonas
				Lysobacter sp.	6.00E-140	97%	Proteobacteria	Xanthomonadaceae	Lysobacter

		TGGCGAGCTAGAGT							
Oligotype 9	AGGGCGG TCGGAGT GGGACGA ATTC	TGGGGAATATTGGACAACG GGCGCAAGCCTGATCCAGC AATGCCGCGTGGATGAAGA AGGTCTTCGGATTGTAAAG TCCTTTCGACGGGGACGAT GATGACGGTACCCGTAGAA GAAGCCCCGGCTAACTTCG TGCCAGCAGCCGCGGTAAT ACGAAGGGTGCAAGCGTTA CTCGGAATTACTGGGCGTA AAGCGTGCGTAGGTGGTTG TTTAAGTCTGCTGTGAAAG CCCCGGGCTCAACCTGGGA ATTGCAGTGGATACTGGGC GACTAGAGTGTGGTAGAGG ACAGTGGAATTTTCC	3	Uncultured bacterium clone	2.00E-125	95%	-	-	-
				Uncultured Roseomonas sp.	5.00E-101	94%	Proteobacteria	Acetobacteraceae	Roseomonas
				Uncultured Acetobacteraceae bacterium	1.00E-101	94%	Proteobacteria	Acetobacteraceae	-
				Uncultured Gluconobacter sp.	2.00E-100	91%	Proteobacteria	Acetobacteraceae	Gluconobacter
Oligotype 10	TCTTTCAC TCCTGCA GACTCAC TAG	TAGGGAATCTTCCACAATG GACGAAGGTCTGATGGAGC AACGCCGCGTGAGTGAAGA AGGTTTTCGGATCGTAAAA CTCTGTTGTAAGGGAAGAA CACGTACGAGAGTAACTGC TCGTACCTTGACGGTACCT TATTAGAAAGCCACGGCTA ACTACGTGCCGGCAGCCGC GGTAATACGAAGGGTGCAA GCGTTACTCGGAATTACTG GGCGTAAAGCGTGCGTAGG TGGTTGTTTAAGTCTGCTGT GAAAGCCCCGGGCTCAACC TGGGAATTGCAGTGGATAC TGGGCGACTAGAGT	2	Uncultured delta proteobacterium clone	5.00E-111	91%	Proteobacteria	-	-
Oligotype 11	TCGAACA CTCCTGC AGACTCA CTAG	TGGGGAATATTGGACAATG GGCGCAAGCCTGATCCAGC CATACCGCGTGGGTGAAGA AGGCCTTCGGGTTGTAAAG CCCTTTTGTTGGGAAAGAA ATCCTGCGGATTAATACCC GGCAGGGATGACGGTACCC AAAGAATAAGCACCGGCTA ACTTCGTGCCAGCAGCCGC GGTAATACGAAGGGTGCAA	2	Uncultured proteobacterium clone	1.00E-152	99%	Proteobacteria	-	-
				Pseudomonas sp.	2.00E-144	98%	Proteobacteria	Pseudomonadaceae	Pseudomonas
				Stenotrophomonas sp.	2.00E-144	98%	Proteobacteria	Xanthomonadaceae	Stenotrophomonas
				Uncultured Pseudoxanthomonas sp.	2.00E-144	98%	Proteobacteria	Xanthomonadaceae	Pseudoxanthomonas

		GCGTTACTCGGAATTACTGGGCGTAAAGCGTGCGTAGGTGGTCGTTTAAGTCTGCTGTGAAAGCCCCGGGCTCAACCTGGGAATTGCAGTGGATACTGGGCGACTAGAGT		Xanthomonas sp.	1.00E-142	98%	Proteobacteria	Xanthomonadaceae	Xanthomonas
Oligotype 12	TCAAACCTCTAGCAGTCTGGCTAGT	TGGGGAATATTGGACAATGGGCGCAAGCCTGATCCAGCCATACCGCGTGGGTGAAGAAGGCCTTCGGGTTGTAAAGCCCTTTTGTTGGGAAAGAAATCCTGCAGGTTAATAACCAGTAGGGATGACGGTACCCAAAGAATAAGCACCGGCTAACTTCGTGCCAGCAGCCGCGGTAATACGAAGGGTGCAAGCGTTACTCGGATTACTGGGCGTAAAGCGTGCGTAGGTGGTTGTTTAAGTCTGCTGTGAAAGCCCCGGGCTCAACCTGGGAATTGCAGTGGATACTGGGCGACTAGAGTG	2	Uncultured bacterium clone	2.00E-149	99%	-	-	-
				Pseudoxanthomonas sp.	2.00E-149	99%	Proteobacteria	Xanthomonadaceae	Pseudoxanthomonas
				Pseudomonas sp.	2.00E-149	99%	Proteobacteria	Pseudomonadaceae	Pseudomonas
				Xanthomonas sp.	2.00E-149	99%	Proteobacteria	Xanthomonadaceae	Xanthomonas
				Stenotrophomonas sp.	5.00E-146	98%	Proteobacteria	Xanthomonadaceae	Stenotrophomonas
				Pseudoxanthomonas ginsengisoli	5.00E-146	98%	Proteobacteria	Xanthomonadaceae	Pseudoxanthomonas
Oligotype 13	TCAAACACTTCTGCAAACTTGCTAG	TGGGGAATATTGGACAATGGGCGCAAGCCTGATCCAGCCATACCGCGTGGGTGAAGAAGGCCTTCGGGTTGTAAAGCCCTTTTGTTGGGAAAGAAATCCTGCAGGTTAATAACCAGTAGGGATGACGGTACCCAAAGAATAAGCACCGGCTAACTTCGTGCCAGCAGCCGCGGTAATACGAAGGGTGCAAGCGTTACTCGGAATTACTGGGCGTAAAGGGTGCGTAGGCGGTTATTTAAGTCTGTTGTGAAAGCCCTGGGCTCAACCTGGGAATGGCAATGGATACTGGGTAGCTAGAGT	2	Uncultured bacterium clone	1.00E-136	98%	-	-	-
				Pseudoxanthomonas yeongjuensis	2.00E-139	97%	Proteobacteria	Xanthomonadaceae	Pseudoxanthomonas
				Stenotrophomonas sp.	2.00E-139	97%	Proteobacteria	Xanthomonadaceae	Stenotrophomonas
				Pseudoxanthomonas sp.	1.00E-137	97%	Proteobacteria	Xanthomonadaceae	Pseudoxanthomonas
				Pseudomonas sp.	1.00E-137	97%	Proteobacteria	Pseudomonadaceae	Pseudomonas
				Xanthomonas sp.	1.00E-137	97%	Proteobacteria	Xanthomonadaceae	Xanthomonas
Oligotype 14	TCAAACACGGTCTGCATACGGACTA	TGGGGAATATTGGACAATGGGCGCAAGCCTGATCCAGCCATACCGCGTGGGTGAAGAAGGCCTTCGGGTTGTAAAGCCCTTTTGTTGGGAAAGAAATCCTGCAGGTTAATAACC	2	Uncultured bacterium clone	8.00E-149	99%	-	-	-
				Pseudoxanthomonas sp.	8.00E-149	99%	Proteobacteria	Xanthomonadaceae	Pseudoxanthomonas
				Pseudomonas sp.	8.00E-149	99%	Proteobacteria	Pseudomonadaceae	Pseudomonas

		AGTAGGGATGACGGTACCCAAAGAATAAGCACCGGCTAACTTCGTGCCAGCAGCCGCGGTAATACGAAGGGTGCAAGCGTTACTCGGAATTACTGGGGCGTAAAGCGTGCGTAGGTGGTTGTTTAAGTCTGCTGTGAAAGCCCCGGGCTCAACCTGGGAATTGCAGTGGATACTGGGCGACTAGAG		Xanthomonadaceae bacterium	8.00E-149	99%	Proteobacteria	Xanthomonadaceae	-
				Xanthomonas sp.	8.00E-149	99%	Proteobacteria	Xanthomonadaceae	Xanthomonas
				Stenotrophomonas sp.	2.00E-145	98%	Proteobacteria	Xanthomonadaceae	Stenotrophomonas
Oligotype 15	GAGGTTCTTCCTGCAGACTCACTAG	TGGGGGAATATTGGACAATGGGCGCAAGCCTGATCCAGCCATACCGCGTGGGTGAAGAAGGCCTTCGGGTTGTAAGCCCTTTTGTTGGGAAGAAATCCTGCAGGTTAATAACCAGTAGGGATGACGGTACCCAAAGAATAAGCACCGGCTAACTTCGTGCCAGCAGCCGCGGTAATACGAAGGGTGCAAGCGTTACTCGGAATTACTGGGCGTAAAGCGTGCGGTAGGTGGTTGTTTAAGTCTGCTGTGAAAGCCCCGGGCTCAACCTGGGAATTGCAGTGGATACTGGGCGACTAGAGT	2	Uncultured bacterium clone	5.00E-146	98%	-	-	-
				Pseudoxanthomonas sp.	6.00E-145	98%	Proteobacteria	Xanthomonadaceae	Pseudoxanthomonas
				Pseudomonas sp.	6.00E-145	98%	Proteobacteria	Pseudomonadaceae	Pseudomonas
				Xanthomonadaceae bacterium	6.00E-145	98%	Proteobacteria	Xanthomonadaceae	-
				Xanthomonas sp.	6.00E-145	98%	Proteobacteria	Xanthomonadaceae	Xanthomonas
				Stenotrophomonas sp.	1.00E-141	98%	Proteobacteria	Xanthomonadaceae	Stenotrophomonas
Oligotype 16	CGGGTTCTCTAGCACTCTGACTAGT	TAGGGAATATTGGACAATGGGCGCAAGCCTGATCCAGCCATGCCGCGTGTGTGAAGAAGGCCTTCGGTTGTAAAGCACTTTGTCCGGAAAGAAAAGACGCTGGGTTAACACCCTGGTGTCCTGACGGTACCGGAAGAATAAGCACCGGCTAACTTCGTGCCAGCAGCCGCGGTAATACGAAGGGTGCAAGCGTTACTCGGAATTACTGGGCGTAAAGCGTGCGTAGGTGGTTTGTTAAGTCTGATGTGAAAGCCCTGGGCTCAACCTGGGAATGGCACTGGATACTGGCGAGCTAGAGTG	2	Uncultured gamma proteobacterium	2.00E-149	99%	Proteobacteria	-	-
				Uncultured Thermomonas sp.	1.00E-136	99%	Proteobacteria	Xanthomonadaceae	Thermomonas
				Thermomonas sp.	2.00E-139	97%	Proteobacteria	Xanthomonadaceae	Thermomonas
				Lysobacter sp.	1.00E-136	97%	Proteobacteria	Xanthomonadaceae	Lysobacter
Oligotype 17	CCCGGTTTATCCTTGC	TGGGGAATTTTTGGACAATGGGCGCAAGCCTGATCCAG	2	Uncultured beta proteobacterium	8.00E-114	94%	Proteobacteria	-	-

	ATAGCGACG	CCATGCCGCGTGTGTGAAAGAAGGCCTTCGGGTTGTAAAGCACTTTCGGCCGGAACGAAATCGCGCGGGCTATTACCCCGCGTGGATGACGGTACCGGAAGAAGAAGCACCGGCTAACTACGTGCCAGCAGCCGCGGTAATACGAAGGGTGCAAGCGTTACTCGGAATTACTGGGCGTAAAGCGTGCGTAGGTGGTTGTTTAAGTCTGCTGTGAAAGCCCCGGGCTCAACCTGGGAATTGCAGTGGATACTGGGCGACTAGA		clone					
				Lysobacter sp.	1.00E-136	97%	Proteobacteria	Xanthomonadaceae	Lysobacter
				Xanthomonadaceae bacterium	4.00E-112	92%	Proteobacteria	Xanthomonadaceae	-
				Rehaibacterium terrae	2.00E-110	92%	Proteobacteria	Xanthomonadaceae	Rehaibacterium
				Arenimonas sp.	4.00E-112	92%	Proteobacteria	Xanthomonadaceae	Arenimonas
				Stenotrophomonas sp.	4.00E-112	92%	Proteobacteria	Xanthomonadaceae	Stenotrophomonas
				Pseudoxanthomonas sp.	4.00E-112	98%	Proteobacteria	Xanthomonadaceae	Pseudoxanthomonas
Oligotype 18	CCCGCATAGGTCGGCATACCAGCTA	TAGGGAATATTGGACAATGGGCGCAAGCCTGATCCAGCCATGCCGCGTGTGTGAAGAAGGCCTTCGGGTTGTAAAGCACTTTTGTTCCGGAAAGAAAAGCGCTGGGTTAACACCCTGGTGTCCTGACGGTACCGGAAGAATAAGCACCGGCTAACTTCGTGCCAGCAGCCGCGGTAATACGAAGGGTGCAAGCGTTACTCGGAATTACTGGGCGTAAAGCGTGCGTAGGTGGTTTGTTAAGTCTGATGTGAAAGCCCTGGGCTCAACCTGGGAATGGCACTGGATACTGGCGAGCTAGAG	2	Uncultured gamma proteobacterium	4.00E-152	99%	Proteobacteria	-	-
				Uncultured Thermomonas sp.	2.00E-140	99%	Proteobacteria	Xanthomonadaceae	Thermomonas
				Thermomonas sp.	4.00E-142	98%	Proteobacteria	Xanthomonadaceae	Thermomonas
				Lysobacter sp.	2.00E-139	97%	Proteobacteria	Xanthomonadaceae	Lysobacter
Oligotype 19	TTAAACACTCTCTGCATACGGACTA	TGGGGAATATTGGACAATGGGCGCAAGCCTGATCCAGCCATACCGCGTGGGTGAAGAAGGCCTTCGGGTTGTAAAGCCCTTTTGTTGGGAAAGAAATCCTGTAGGTTAATAACCAGTAGGGATGACGGTACCCAAAGAATAAGCACCGGCTAACTTCGTGCCAGCAGCCGCGGTAATACGAAGGGTGCAAGCGTTACTCGGAATTACTGGGCGTAAAGCGTGCGTAGGTGGTTGTTTAAGTCTGCTGTGAAAGCCCCCGGGCTCAACCTGGGAATTGCAGTGGATA	1	Pseudoxanthomonas sp.	8.00E-149	99%	Proteobacteria	Xanthomonadaceae	Pseudoxanthomonas
				Pseudomonas sp.	4.00E-147	99%	Proteobacteria	Pseudomonadaceae	Pseudomonas
				Xanthomonas sp.	4.00E-147	99%	Proteobacteria	Xanthomonadaceae	Xanthomonas
				Stenotrophomonas sp.	4.00E-147	99%	Proteobacteria	Xanthomonadaceae	Stenotrophomonas

		CTGGGCGACTAGAG							
Oligotype 20	TCTTTTATTCCTGCAGACTCACTAG	TAGGGAATCTTCCACAATGGACGAAGGTCTGATGGAGCAACGCCGCGTGAGTGAAGAAGGTTTTCGGATCGTAAAACTCTGTTGTAAGGGAAGAACACGTACGAGAGTAACTGCTCGTACCTTGACGGTACCTTATTAGAAAGCCACGGCTAACTACGTGCCAGCAGCCGCGGTAATACGTAGGCGGCAAGCGTTGTCCGGAATCATTGGGCGTAAAGCGTGCGTAGGTGGTTGTTTAAGTCTGCTGTGAAAGCCCCGGGCTCAACCTGGGAATTGCAGTGGATACTGGGCGACTAGAGT	1	Uncultured bacterium clone	2.00E-119	93%	-	-	-
Oligotype 21	TCCAACACTCCTGCGGACTGGCTAG	TGGGGGAATATTGGACAATGGGCGCAAGCCTGATCCAGCCATACCGCGTGGGTGAAGAAGGCCTTCGGGTTGTAAGCCCTTTTGTTGGGAAAGAAATCCTGCAGGTTAATAACCCGTAGGGATGACGGTACCCAAAGAATAAGCACCGGCTAACTTCGTGCCAGCAGCCGCGGTAATACGAAGGGTGCAAGCGTTACTCGGAATTACTGGGCGTAAAGCGTGCGTAGGTGGTTCGTTAAGTCTGCCGTGAAAGCCCCGGGCTCAACCTGGGAATTGCGGTGGATACTGGCGGGCTAGAGT	1	Uncultured gamma proteobacterium	3.00E-143	98%	Proteobacteria	-	-
				Uncultured bacterium clone	6.00E-140	97%	-	-	-
				Luteimonas sp.	1.00E-136	97%		Xanthomonadaceae	Luteimonas
				Pseudoxanthomonas sp.	6.00E-135	96%	Proteobacteria	Xanthomonadaceae	Pseudoxanthomonas
				Pseudomonas sp.	6.00E-135	96%	Proteobacteria	Pseudomonadaceae	Pseudomonas
Oligotype 22	TCAATTTGTGAGCAGTCTGGCTAGT	TGGGGAATATTGGACAATGGGCGCAAGCCTGATCCAGCCATACCGCGTGGGTGAAGAAGGCCTTCGGGTTGTAAAGCCCTTTTGTTGGGAAAGAAATCCTGCAGGTTAATAACCAGTAGGATGACGGTACCCAAAGAATAAGCACCGGCTAACTTCGTGCCAGCAGCCGCGGTAATACGAAGGGTGCAAGCGTTACTCGGATTACTGGG	1	Uncultured bacterium clone	5.00E-146	98%	-	-	-
				Pseudoxanthomonas sp.	5.00E-146	98%	Proteobacteria	Xanthomonadaceae	Pseudoxanthomonas
				Pseudomonas sp.	5.00E-146	98%	Proteobacteria	Pseudomonadaceae	Pseudomonas
				Xanthomonas sp.	5.00E-146	98%	Proteobacteria	Xanthomonadaceae	Xanthomonas
				Stenotrophomonas sp.	1.00E-142	98%	Proteobacteria	Xanthomonadaceae	Stenotrophomonas

		CGTAAAGCGTGCGTAGGTGGTTGTTTAAGTCTGCTGTGAAAGCCCCCGGGCTCAACCTGGGAATTGCAGTGGATACTGGGCGACTAGAGTG							
Oligotype 23	TCAAACATTCCTGCAGACTCACTAG	TGGGGAATATTGGACAATGGGCGCAAGCCTGATCCAGCCATACCGCGTGGGTGAAGAAGGCCTTCGGGTTGTAAAGCCCTTTTGTTGGGAAAGAAATCCTGCAGGTTAATAACCAGTAGGGATGACGGTACCCAAAGAATAAGCACCGGCTAACTTCGTGCCAGCAGCCGCGGTAATACGAAGGGTGCAAGCGTTACTCGGAATTATTGGGCGTAAAGCGTGCGTAGGTGGTTGTTTAAGTCTGCTGTGAAAGCCCCGGGCTCAACCTGGGAATTGCAGTGGATACTGGGCGACTAGAGT	1	Uncultured bacterium clone	2.00E-149	99%	-	-	-
				Pseudoxanthomonas sp.	2.00E-149	99%	Proteobacteria	Xanthomonadaceae	Pseudoxanthomonas
				Pseudomonas sp.	2.00E-149	99%	Proteobacteria	Pseudomonadaceae	Pseudomonas
				Xanthomonas sp.	2.00E-149	99%	Proteobacteria	Xanthomonadaceae	Xanthomonas
Oligotype 24	TCAAACACTCCTTGCATACGGACTA	TGGGGAATATTGGACAATGGGCGCAAGCCTGATCCAGCCATACCGCGTGGGTGAAGAAGGCCTTCGGGTTGTAAAGCCCTTTTGTTGGGAAAGAAATCCTGCAGGTTAATAACCAGTAGGGATGACGGTACCCAAAGAATAAGCACCGGCTAACTTCGTGCCAGCAGCCGCGGTAATACGAAGGGTGCAAGCGTTACTCGGAATTACTGGGCGTAAAGCGTGCGTAGGTGGTTGTTTAAGTCTGCTGTGAAAGCCCCGGGCTCAACCTGGGGAATTGCAGTGGATACTGGGCGACTAGAG	1	Uncultured bacterium clone	8.00E-149	99%	-	-	-
				Pseudoxanthomonas sp.	8.00E-149	99%	Proteobacteria	Xanthomonadaceae	Pseudoxanthomonas
				Pseudomonas sp.	8.00E-149	99%	Proteobacteria	Pseudomonadaceae	Pseudomonas
				Xanthomonas sp.	8.00E-149	99%	Proteobacteria	Xanthomonadaceae	Xanthomonas
				Stenotrophomonas sp.	2.00E-145	98%	Proteobacteria	Xanthomonadaceae	Stenotrophomonas
Oligotype 25	TCAAACACTCCTGCAGACTAGCTAG	TGGGGAATATTGGACAATGGGCGCAAGCCTGATCCAGCCATACCGCGTGGGTGAAGAAGGCCTTCGGGTTGTAAAGCCCTTTTGTTGGGAAAGAAATCCTGCAGGTTAATAACCAGTAGGGATGACGGTACCC	1	Uncultured bacterium clone	2.00E-139	97%	-	-	-
				Pseudoxanthomonas sp.	2.00E-139	97%	Proteobacteria	Xanthomonadaceae	Pseudoxanthomonas
				Pseudomonas sp.	2.00E-139	97%	Proteobacteria	Pseudomonadaceae	Pseudomonas

		AAAGAATAAGCACCGGCTA<br>ACTTCGTGCCAGCAGCCGC<br>GGTAATACGAAGGGTGCAA<br>GCGTTACTCGGAATTACTG<br>GGCGTAAAGCGTGCGTAGA<br>CGGCTTGTTAAGTCTGCTG<br>TGAAAGCCCTGGGCTCAAC<br>CTGGGAACTGCAGTGGATA<br>CTGGCAGGCTAGAGT							
Oligotype 26	TCAAACA<br>CTCCTAG<br>CAATAGC<br>GACG	TGGGGAATATTGGACAATG<br>GGCGCAAGCCTGATCCAGC<br>CATACCGCGTGGGTGAAGA<br>AGGCCTTCGGGTTGTAAAG<br>CCCTTTTGTTGGGAAAGAA<br>ATCCTGCAGGTTAATAACC<br>AGTAGGGATGACGGTACCC<br>AAAGAATAAGCACCGGCTA<br>ACTTCGTGCCAGCAGCCGC<br>GGTAATACGAAGGGTGCAA<br>GCGTTACTCGGAATTACTG<br>GGCGTAAAGCGTGCGTAGG<br>TGGTTGTTTAAGTCTGCTGT<br>GAAAGCCCCGGGCTCAACC<br>TGGGAATTAGCAGTAGGAT<br>ACTGGGCGACTAGA	1	Uncultured bacterium clone	4.00E-147	99%	-	-	-
				Pseudoxanthomonas sp.	4.00E-147	99%	Proteobacteria	Xanthomonadaceae	Pseudoxanthomonas
				Pseudomonas sp.	4.00E-147	99%	Proteobacteria	Pseudomonadaceae	Pseudomonas
				Stenotrophomonas sp.	8.00E-144	98%	Proteobacteria	Xanthomonadaceae	Stenotrophomonas
Oligotype 27	TCAAACA<br>CTCCCTGC<br>ATACGGA<br>CTA	TGGGGAATATTGGACAATG<br>GGCGCAAGCCTGATCCAGC<br>CATACCGCGTGGGTGAAGA<br>AGGCCTTCGGGTTGTAAAG<br>CCCTTTTGTTGGGAAAGAA<br>ATCCTGCAGGTTAATAACC<br>AGTAGGGATGACGGTACCC<br>AAAGAATAAGCACCGGCTA<br>ACTTCGTGCCAGCAGCCGC<br>GGTAATACGAAGGGTGCAA<br>GCGTTACTCGGAATTACTG<br>GGCGTAAAGCGTGCGTAGG<br>TGGTTGTTTAAGTCTGCTGT<br>GAAAGCCCCGGGCTCAACC<br>CTGGGAATTGCAGTGGATA<br>CTGGGCGACTAGAG	1	Uncultured bacterium clone	8.00E-149	99%	-	-	-
				Pseudoxanthomonas sp.	8.00E-149	99%	Proteobacteria	Xanthomonadaceae	Pseudoxanthomonas
				Pseudomonas sp.	8.00E-149	99%	Proteobacteria	Pseudomonadaceae	Pseudomonas
				Stenotrophomonas sp.	2.00E-145	98%	Proteobacteria	Xanthomonadaceae	Stenotrophomonas
Oligotype 28	TCAAACA<br>CGGGAAT<br>TGGATGG	TGGGGAATATTGGACAATG<br>GGCGCAAGCCTGATCCAGC<br>CATACCGCGTGGGTGAAGA	1	Uncultured bacterium clone	6.00E-145	98%	-	-	-
				Pseudoxanthomo	6.00E-145	98%	Proteoba	Xanthomona	Pseudoxantho

	CGAA	AGGCCTTCGGGTTGTAAAG CCCTTTTGTTGGGAAAGAA ATCCTGCAGGTTAATAACC AGTAGGGATGACGGTACCC AAAGAATAAGCACCGGCTA ACTTCGTGCCAGCAGCCGC GGTAATACGAAGGGTGCAA GCGTTACTCGGAATTACTG GGCGTAAAAGCGTGCGTAG GTGGTTGTTTAAGTCTGCT GTGAAAAGCCCCCGGGCTC AACCTGGGAATTGCAGTGG ATACTGGGCGACTAG		nas sp.			cteria	daceae	monas
				Pseudomonas sp.	6.00E-145	98%	Proteobacteria	Pseudomonadaceae	Pseudomonas
				Stenotrophomonas sp.	1.00E-141	98%	Proteobacteria	Xanthomonadaceae	Stenotrophomonas
Oligotype 29	TCAAACACGGCCTTGCATAGCGACG	TGGGGAATATTGGACAATG GGCGCAAGCCTGATCCAGC CATACCGCGTGGGTGAAGA AGGCCTTCGGGTTGTAAAG CCCTTTTGTTGGGAAAGAA ATCCTGCAGGTTAATAACC AGTAGGGATGACGGTACCC AAAGAATAAGCACCGGCTA ACTTCGTGCCAGCAGCCGC GGTAATACGAAGGGTGCAA GCGTTACTCGGAATTACTG GGCGTAAAAGCGTGCGTAG GTGGTTGTTTAAGTCTGCT GTGAAAGCCCCCGGGCTCA ACCTGGGAATTGCAGTGGA TACTGGGCGACTAGA	1	Uncultured bacterium clone	4.00E-147	99%	-	-	-
				Pseudoxanthomonas sp.	4.00E-147	99%	Proteobacteria	Xanthomonadaceae	Pseudoxanthomonas
				Pseudomonas sp.	4.00E-147	99%	Proteobacteria	Pseudomonadaceae	Pseudomonas
				Stenotrophomonas sp.	8.00E-144	98%	Proteobacteria	Xanthomonadaceae	Stenotrophomonas
Oligotype 30	TCAAACACGGCAATTGGATGGCGAA	TAGGGAATATTGGACAATG GGCGCAAGCCTGATCCAGC CATACCGCGTGGGTGAAGA AGGCCTTCGGGTTGTAAAG CCCTTTTGTTGGGAAAGAA ATCCTGCAGGTTAATAACC AGTAGGGATGACGGTACCC AAAGAATAAGCACCGGCTA ACTTCGTGCCAGCAGCCGC GGTAATACGAAGGGTGCAA GCGTTACTCGGAATTACTG GGCGTAAAGCGTGCGTAGG TGGTTGTTTAAAGTCTGCT GTGAAAGCCCCCGGGCTCA AACCTGGGAATTGCAGTGG ATACTGGGCGACTAG	1	Uncultured bacterium clone	8.00E-144	98%	-	-	-
				Pseudoxanthomonas sp.	8.00E-144	98%	Proteobacteria	Xanthomonadaceae	Pseudoxanthomonas
				Pseudomonas sp.	8.00E-144	98%	Proteobacteria	Pseudomonadaceae	Pseudomonas
				Stenotrophomonas sp.	2.00E-140	98%	Proteobacteria	Xanthomonadaceae	Stenotrophomonas

Oligotype 31	TCAAACACCTAGCAGTCTGGCTAGT	TGGGGAATATTGGACAATGGGCGCAAGCCTGATCCAGCCATACCGCGTGGGTGAAGAAGGCCTTCGGGTTGTAAAGCCCTTTTGTTGGGAAAGAAATCCTGCAGGTTAATAACCAGTAGGGATGACGGTACCCAAAGAATAAGCACCGGCTAACTTCGTGCCAGCAGCCGCGGTAATACGAAGGGTGCAAGCGTTACTCGGAATTACTGGGCGTAAAGCGTGCGTAGGTGGTTGTTAAGTCTGCTGTGAAAGCCCCGGGCTCAACCTGGGAATTGCAGTGGATACTGGGCGACTAGAGTG	1	Uncultured bacterium clone	2.00E-149	99%	-	-	-
				Pseudoxanthomonas sp.	2.00E-149	99%	Proteobacteria	Xanthomonadaceae	Pseudoxanthomonas
				Pseudomonas sp.	2.00E-149	99%	Proteobacteria	Pseudomonadaceae	Pseudomonas
				Stenotrophomonas sp.	5.00E-146	98%	Proteobacteria	Xanthomonadaceae	Stenotrophomonas
Oligotype 32	TCAAAATTATCCTTGCATAGCGACG	GTGGGGAATATTGGACAATGGGCGCAAGCCTGATCCAGCCATACCGCGTGGGTGAAGAAGGCCTTCGGGTTGTAAAGCCCTTTTGTTGGGAAGAAATCCTGCAGGTTAATAACCAGTAGGGATGACGGTACCCAAAGAATAAGCACCGGCTAACTTCGTGCCAGCAGCCGCGGTAATACGAAGGGTGCGAAGCGTTACTCGGAAATTACTGGGCGTAAAGCGTGCGTAGGTGGTTGTTTAAGTCTGCTGTGAAAGCCCCGGGCTCAACCTGGGAATTGCAGTGGATACTGGGCGACTAGA	1	Uncultured bacterium clone	2.00E-145	98%	-	-	-
				Pseudoxanthomonas sp.	2.00E-145	98%	Proteobacteria	Xanthomonadaceae	Pseudoxanthomonas
				Pseudomonas sp.	2.00E-145	98%	Proteobacteria	Pseudomonadaceae	Pseudomonas
				Stenotrophomonas sp.	4.00E-142	98%	Proteobacteria	Xanthomonadaceae	Stenotrophomonas
Oligotype 33	TCAAAATATGCCTTGCATAGCGACG	TGGGGAATATTGGACAATGGGCGCAAGCCTGATCCAGCCATACCGCGTGGGTGAAGAAGGCCTTCGGGTTGTAAAGCCCTTTTGTTGGGAAAGAAATCCTGCAGGTTAATAACCAGTAGGGATGACGGTACCCAAAGAATAAGCACCGGCTAACTTCGTGCCAGCAGCCGCGGTAATACGAAGGGTGCAAGCGTTAACTCGGAATTACTGGGCGTAAAGCGTGCGTAGGTGGTTGTTAAGTCCTGCT	1	Uncultured bacterium clone	8.00E-144	98%	-	-	-
				Pseudoxanthomonas sp.	8.00E-144	98%	Proteobacteria	Xanthomonadaceae	Pseudoxanthomonas
				Pseudomonas sp.	8.00E-144	98%	Proteobacteria	Pseudomonadaceae	Pseudomonas
				Stenotrophomonas sp.	2.00E-140	97%	Proteobacteria	Xanthomonadaceae	Stenotrophomonas

		GTGAAAGCCCCCGGGCTCAACCTGGGAATTGCAGTGGATACTGGGCGACTAGA							
Oligotype 34	TCAAAATAGGTCTGCATACGGACTA	GGGGAATATTGGACAATGGGCGCAAGCCTGATCCAGCCCATACCGCGTGGGTGAAGAAGGCCTTCGGGTTGTAAAGCCCTTTTGTTGGGAAAGAAATCCTGCAGGTTAATAACCAGTAGGGATGACGGTACCCAAAAGAATAAGCACCGGCTAACTTCGTGCCAGCAGCCGCGGTAATACGAAGGGTGCAAGCGTTACTCGGAATTACTGGGCGTAAAGCGTGCGTAGGTGGTTGTTTAAGTCTGCTGTGAAAGCCCCGGGCTCAACCTGGGAATTGCAGTGGATACTGGGCGACTAGAG	1	Uncultured bacterium clone	4.00E-147	99%	-	-	-
				Pseudoxanthomonas sp.	4.00E-147	99%	Proteobacteria	Xanthomonadaceae	Pseudoxanthomonas
				Pseudomonas sp.	4.00E-147	99%	Proteobacteria	Pseudomonadaceae	Pseudomonas
				Stenotrophomonas sp.	8.00E-144	98%	Proteobacteria	Xanthomonadaceae	Stenotrophomonas
Oligotype 35	TCAAAATAGGCCTTGCATAGCGACG	TGGGGAATATTGGACAATGGGCGCAGGCCTGATCCAGCCATACCGCGTGGGTGAAGAAGGCCTTCGGGTTGTAAAGCCCTTTTGTTGGGAAAGAAATCCTGCAGGTTAATAACCAGTAGGGATGACGGTACCCAAAGAATAAAGCACCGGCTAACTTCGTGCCAGCAGCCGCGGTAATACGAAGGGTGCAAGCGTTACTCGGAATTACTGGGCGTAAAGCGTGCGTAGGTGGTTGTTTAAGTCTGCTGTGAAAGCCCCCGGGCTCAACCTGGGAATTGCAGTGGATACTGGGCGACTAGA	1	Uncultured bacterium clone	2.00E-145	98%	-	-	-
				Pseudoxanthomonas sp.	2.00E-145	98%	Proteobacteria	Xanthomonadaceae	Pseudoxanthomonas
				Pseudomonas sp.	2.00E-145	98%	Proteobacteria	Pseudomonadaceae	Pseudomonas
				Stenotrophomonas sp.	4.00E-142	98%	Proteobacteria	Xanthomonadaceae	Stenotrophomonas
Oligotype 36	TCAAAATAATGAAATTGGATGGCGT	GGGGGAATATTGGACAATGGGCGCAAGCCTGATCCAGCCATACCGCGTGGGTGAAGAAGGCCTTCGGGTTGTAAAGCCCTTTTGTTGGGAAAGAAATCCTGCAGGTTAATAACCAGTAGGGATGACGGTACCCAAAGAATAAGCACCGGCTAACTTCGTGCCAGCAGCCGC	1	Uncultured bacterium clone	3.00E-143	98%	-	-	-
				Pseudoxanthomonas sp.	1.00E-142	98%	Proteobacteria	Xanthomonadaceae	Pseudoxanthomonas
				Pseudomonas sp.	1.00E-142	98%	Proteobacteria	Pseudomonadaceae	Pseudomonas
				Stenotrophomonas sp.	2.00E-139	97%	Proteobacteria	Xanthomonadaceae	Stenotrophomonas

		GGTAATACGAAGGGTGCAA<br>GCGTTAACTCGGAATTACT<br>GGGCGTAAAAGCGTGCGTA<br>GGTGGTTGTTTAAGTCTGC<br>TGTGAAAGCCCCCGGGCTC<br>AACCTGGGGAATTGCAGTG<br>GATACTGGGCGACTA							
Oligotype 37	TAACAGT<br>GATCCGT<br>GTAGGGG<br>GAAT	TGGGGAATTTTCCGCAATG<br>GGCGAAAGCCTGACGGAG<br>CAAGACCGCGTGCGGGAG<br>GAAGGTCTATTGATTGTAA<br>ACCGCTTTTGATAGGGAAG<br>AAGATCTGACGGTACCTAT<br>CGAATCAGCATCGGCTAAC<br>TCCGTGCCAGCAGCCGCGG<br>TAATACGAAGGGTGCAAGC<br>GTTACTCGGAATTACTGGG<br>CGTAAAGCGTGCGTAGGTG<br>GTTGTTTAAGTCTGCTGTG<br>AAAGCCCCGGGCTCAACCT<br>GGGAATTGCAGTGGATACT<br>GGGCGACTAGAGTGTGGTA<br>GAGGACAGTGGAATTTC	1	Uncultured bacterium clone	1.00E-101	90%	-	-	-
Oligotype 38	GTTTCCAC<br>TCCTGCA<br>GACTCAC<br>TAG	TAGGGAATATTGGACAATG<br>GGCGCAAGCCTGATCCAGC<br>CATGCCGCGTGTGTGAAGA<br>AGGCCTTCGGGTTGTAAAG<br>CACTTTTGTCCGGAAAGAA<br>AAGCGCTGGGTTAACACCC<br>TGGTGTCCTGACGGTACCG<br>GAAGAATAAGCACCGGCTA<br>ACTTCGTGCCAGCAGCCGC<br>GGTAATACGAAGGGTGCAA<br>GCGTTACTCGGAATTACTG<br>GGCGTAAAGCGTGCGTAGG<br>TGGTTGTTTAAGTCTGCTGT<br>GAAAGCCCCGGGCTCAACC<br>TGGGAATTGCAGTGGATAC<br>TGGGCGACTAGAGT	1	Uncultured bacterium clone	1.00E-136	98%	-	-	-
				Uncultured Thermomonas sp.	1.00E-137	97%	Proteobacteria	Xanthomonadaceae	Thermomonas
Oligotype 39	GTTTCCAC<br>CTAGCAC<br>TCTGACT<br>AGT	TAGGGAATATTGGACAATG<br>GGCGCAAGCCTGATCCAGC<br>CATGCCGCGTGTGTGAAGA<br>AGGCCCTTGGGTTGTAAGC<br>ACTTTTGTCCGGAAAGAAA	1	Uncultured bacterium clone	1.00E-142	98%	-	-	-
				Uncultured Thermomonas sp.	5.00E-141	97%	Proteobacteria	Xanthomonadaceae	Thermomonas

		AGACGCTGGGTTAACACCC<br>TGGTGTCCTGACGGTACCG<br>GAAGAATAAGCACCGGCTA<br>ACTTCGTGCCAGCAGCCGC<br>GGTAATACGAAGGGTGCAA<br>GCGTTACTCGGAATTACTG<br>GGCGTAAAGCGTGCGTAGG<br>TGGTTTGTTAAGCTGATGT<br>GAAAGCCCTGGGCTCAACC<br>TGGGAATGGCACTGGATAC<br>TGGCGAGCTAGAGTG							
Oligotype 40	GTTTCATA<br>GGTCGGC<br>ATACCAG<br>CTA	TAGGGAATATTGGACAATG<br>GGCGCAAGCCTGATCCAGC<br>TATGCCGCGTGTGTGAAGA<br>AGGCCTTCGGGTTGTAAAG<br>CACTTTTGTCCGGAAAGAA<br>AAGCGCTGGGTTAACACCC<br>TGGTGTCCTGACGGTACCG<br>GAAGAATAAGCACCGGCTA<br>ACTTCGTGCCAGCAGCCGC<br>GGTAATACGAAGGGTGCAA<br>GCGTTTACTCGGAATTACT<br>GGGCGTAAAGCGTGCGTAG<br>GTGGTTTGTTAAGTCTGAT<br>GTGAAAGCCCTGGGCTCAA<br>CCTGGGAATGGCACTGGAT<br>ACTGGCGAGCTAGAG	1	Uncultured bacterium clone	4.00E-147	99%	-	-	-
				Uncultured beta proteobacterium clone	8.00E-149	99%	Proteobacteria	-	-
				Thermomonas sp.	2.00E-140	97%	Proteobacteria	Xanthomonadaceae	Thermomonas
				Lysobacter sp.	1.00E-137	97%	Proteobacteria	Xanthomonadaceae	Lysobacter
Oligotype 41	GTTTCAA<br>CTTCTGCA<br>AACTTGC<br>TAG	TAGGGAATATTGGACAATG<br>GGCGCAAGCCTGATCCAGC<br>CATGCCGCGTGTGTGAAGA<br>AGGCCTTCGGGTTGTAAAG<br>CACTTTTGTCCGGAAAGAA<br>AAGCGCTGGGTTAACACCC<br>TGGTGTCCTGACGGTACCG<br>GAAGAATAAGCACCGGCTA<br>ACTTCGTGCCAGCAGCCGC<br>GGTAATACGAAGGGTGCAA<br>GCGTTAATCGGAATTACTG<br>GGCGTAAAGGGTGCGTAGG<br>CGGTTACTTAAGTCTGTTGT<br>GAAAGCCCTGGGCTCAACC<br>TGGGAATGGCAATGGATAC<br>TGGGTAGCTAGAGT	1	Uncultured Thermomonas sp.	4.00E-132	98%	Proteobacteria	Xanthomonadaceae	Thermomonas
				Uncultured proteobacterium clone	2.00E-139	97%	Proteobacteria	-	-
				Lysobacter sp.	4.00E-132	97%	Proteobacteria	Xanthomonadaceae	Lysobacter
Oligotype	GTTCCAT	TGGGGAATATTGGACAATG	1	Uncultured	6.00E-130	99%	-	-	-

42	AGGTCGG CATACGA GCTA	GGGGCAACCCTGATCCAGC AATGCCGCGTGTGTGAAGA AGGCCTTCGGGTTGTAAAG CACTTTTATTGGGAACGAA AAGCGATGGGTTAATACCT TATCGTGCTGACGGTACCC AAGGAATAAGCACCGGCTA ACTTCGTGCCAGCAGCCGC GGTAATACGAAGGGTGCAA AGCGTTAATCGGAATTACT GGGCGTAAAGGGTGCGTAG GCGGTTATTTAAGTCTGTT GTGAAAGCCCTGGGCTCAA CCTGGGAATGGCAATGGAT ACTGGGTAGCTAGAG		bacterium clone					
				Uncultured Rhodanobacter sp.	8.00E-124	94%	Proteoba cteria	Xanthomona daceae	Rhodanobacte r
				Dyella sp.	2.00E-120	93%	Proteoba cteria	Xanthomona daceae	Dyella
Oligotype 43	GTTCCAA CTTCAAT GGGATGG TAGA	TGGGGAATATTGGACAATG GGGGCAACCCTGATCCAGC AATGCCGCGTGTGTGAAGA AGGCCTTCGGGTTGTAAAG CACTTTTATTGGGAACGAA AAGCGATGGGTTAATACCT TATCGTGCTGACGGTACCC AAGGAATAAGCACCGGCTA ACTTCGTGCCAGCAGCCGC GGTAATACGAAGGGTGCAA GCGTTAATCGGAATTACTG GGCGTAAAGGGTGCGTAGG CGGTTATTTAAGTCTGTTGT CGAAAGCCCCTGGGCGTCA ACCTGGGAATGGCAATGGA TACTGGGTAGCTAG	1	Uncultured bacterium clone	5.00E-126	99%	-	-	-
				Uncultured Rhodanobacter sp.	2.00E-120	94%	Proteoba cteria	Xanthomona daceae	Rhodanobacte r
				Dyella sp.	3.00E-118	93%	Proteoba cteria	Xanthomona daceae	Dyella
Oligotype 44	GTTCCAA CTCCTGC AGACTCA CTAG	TGGGGAATATTGGACAATG GGGGCAACCCTGATCCAGC AATGCCGCGTGTGTGAAGA AGGCCTTCGGGTTGTAAAG CACTTTTATTGGGAACGAG AAGCGATGGGTTAATACCT TATCGTGCTGACGGTACCC AAGGAATAAGCACCGGCTA ACTTCGTGCCAGCAGCCGC GGTAATACGAAGGGTGCAA GCGTTAATCGGAATTACTG GGCGTAAAGCGTGCGTAGG TGGTTGTTTAAGTCTGCTGT GAAAGCCCCGGGCTCAACC	1	Uncultured bacterium clone	2.00E-120	94%	-	-	-
				Acetobacter sp.	2.00E-119	93%	Proteoba cteria	Acetobactera ceae	Acetobacter
				Stenotrophomona s sp.	2.00E-119	93%	Proteoba cteria	Xanthomona daceae	Stenotrophom onas

		TGGGAATTGCAGTGGATAC TGGGCGACTAGAGT							
Oligotype 45	GTGACGG GCGCATG GAGGCTG AGTG	TGGGGAATATTGGACAATG GGCGCAAGCCTGATCCAGC CATACCGCGTGGGTGAAGA AGGCCTTCGGTTGTAAGCC TTTTGTTGGGAAGAAATCC TGCAGGTTAATAACCAGTA GGGATGACGGTACCCAAAG AATAAGCACCGGCTAACTT CGTGCCAGCAGCCGCGGTA ATACGAAGGGTGCAAGCGT TACTCGGAATTACTGGGCG TAAAGCGTGCGTAGGTGGT TGTTTAAGTCTGCTGTGAA AGCCCCGGGCTCAACCTGG GAATTGCAGTGGATACTGG GCGACTAGAGTGTGG	1	Uncultured bacterium clone	2.00E-145	98%	-	-	-
				Pseudoxanthomonas sp.	2.00E-145	98%	Proteobacteria	Xanthomonadaceae	Pseudoxanthomonas
				Pseudomonas sp.	2.00E-145	98%	Proteobacteria	Pseudomonadaceae	Pseudomonas
Oligotype 46	GGCTATA GACTTTG GCAGATG TAGG	TGGGGAATATTGGACAATG GGCGCAAGCCTGATCCAGC CATACCGCGTGGGGGAAGA AGGCTCTTGGGTTGTAAAC CCCTTTTCTCTGGGAAGAA AGTTGTGAAAGCAGCCTGA CGGTACCAGAGGAATCAGC ATCGGCTAACTTCGTGCCA GCAGCCGCGGTAATACGAA GGGTGCAAGCGTTACTCGG AATTACTGGGCGTAAAGCG TGCGTAGGCGGTTTGTTAA GTCTGATGTGAAAGCCCTG GGCTCAACCTGGGAATGGC ATTGGATACTGGCGATCTA GAATGTGGTAGAGGG	1	Lysobacter sp.	2.00E-105	95%	Proteobacteria	Xanthomonadaceae	Lysobacter
				Uncultured bacterium clone	8.00E-104	90%	-	-	-
Oligotype 47	GCAGGAC GGAGTGA TATAGGG TACT	TAGGGAATATTGGACAATG GGTGCGAGCCTGATCCAGC CATGCCGCGTGCAGGAAGA CGGCCTTCTGGGTTGTAAA CTGCTTTTACCAGGGGATA AAAAGACCATGCGTGGTCA ATTGAAGGTACCTGGTGAA TAAGCCACGGCTAACTACG TGCCAGCAGCCGCGGTAAT ACGAAGGGTGCAAGCGTTA	1	Uncultured bacterium clone	2.00E-99	94%	-	-	-

		ATCGGAATTACTGGGCGTA AAGGGTGCGTAGGCGGTTA TTTAAGCCTGTTGTGAAAG CCCTGGGCTCAACCTGGGA ATGGCAATGGATACTGGGT AGCTAGAGTACGGTA							
Oligotype 48	GCAACTG GATCCGT GTAGGGG GAAT	TGGGGAATCTTGCGCAATG GGCGAAAGCCTGACGCAGC AACGCCGCGTGGGGGATGA AGGCTCTCGGGTTGTAAAC CCCTTTCAGCAGGGACGAA TCAAGACGGTACCTGCAGA ATAAGCGCCGGCTAACTTC GTGCCAGCAGCCGCGGTAA TACGAAGGGTGCAAGCGTT ACTCGGAATTACTGGGCGT AAAGCGTGCGTAGGTGGTT GTTTAAGTCTGCTGTGAAA GCCCCGGGCTCAAACCTGG GAATTGCAGTGGATACTGG GCGACTAGAGTGTGGTAGA GGACAGTGGAATTTC	1	Uncultured bacterium clone	3.00E-113	92%	-	-	-
Oligotype 49	GCAACTG GAGTGTG TGGGATG AATC	TGGGGGAATCTTGCACAAT GGGCGAAAGCCTGATGCAG CGACGCCGCGTGCGGGATG AAGGCCCTCGGGTCGTAAA CCGCTTTCAGCAGGGACGA TAGTGACGGTACCTGCAGA AGAAGCCCCGGCCAACTAC GTGCCAGCAGCCGCGGTAA TACGAGGGGTGCAAGCGTT ATTCGGATTTACTGGGCGT AAAGCGTGCGTAGGTGGTT GTTTAAGTCTGCTGTGAAA GCCCCGGGCTCAACCTGGG AATTGCAGTGGATACTGGG CGACTAGAGTGTGGTAGAG GACAGTGGAATTTCC	1	Uncultured bacterium clone	2.00E-114	92%	-	-	-
Oligotype 50	GCAACTG GAGTATG CGGGGCG AATC	TGGGGAATATTGGACAATG GGCGCAAGCCTGATCCAGC AATGCCGCGTGGATGAAGA AGGTCTTCGGATTGTAAAA GTCCTTTCGACGGGGACGA TGATGACGGTACCCGTAGA	1	Uncultured bacterium clone	2.00E-114	92%	-	-	-
				Uncultured Gluconobacter sp.	8.00E-109	91%	Proteoba cteria	Acetobactera ceae	Gluconobacte r
				Roseomonas sp.	8.00E-109	91%	Proteoba cteria	Acetobactera ceae	Roseomonas

		AGAAGCCCCGGCTAACTTCGTGCCAGCAGCCGCGGTAATACGAAGGGGGCTAGCGTTACTCGGAATTACTGGGCGTAAAGCGTGCGTAGGCGGTTTGTTAAGTCCGATGTGAAAGCCCTGGGCTCAACCTGGGAATGGCATGGGATACTGGCGAGCTAGAGTGCGGTAGAGGGGTGCGGAATTCCC							
Oligotype 51	GCAACTGGAGTATACGGGGTGAATC	TGGGGAATATTGGACAATGGGCGAAAGCCTGATCCAGCAATGCCGCGTGAGTGATGAAGGCCTTAGGGTCGTAAAGCTCTTTTTACCCGGGATGATAATGACAGTACCGGGAGAATAAGCTCCGGCTAACTTCGTGCCAGCAGCCGCGGTAATACGAAGGGTGCAAGCGTTACTCGGAATTACTGGGCGTAAAGCGTGCGTAGGCGGTTTGTTAAGTCTGATGTGAAATCCCCGGGCTCAACCTGGGACGTGCATTGGAAACTGGCGATCTAGAGTACGGTAGAGGGTGGTGGAATTCCC	1	Uncultured bacterium clone	8.00E-124	94%	-	-	-
				Uncultured Sphingobium sp.	2.00E-119	93%	Proteobacteria	Sphingomonadaceae	Sphingobium
				Uncultured Burkholderia sp.	1.00E-111	92%	Proteobacteria	Burkholderiaceae	Burkholderia
Oligotype 52	GAGGTTCTCTAGCACTCTGACTAGT	TGGGGAATATTGGACAATGGGCGCAAGCCTGATCCAGCCATACCGCGTGGGTGAAGAAGGCCTTCGGGTTGTAAAGCCCTTTTGTTGGGAAGAAATCCTGCAGGTTAATAACCAGTAGGGATGACGGTACCCAAAGAATAAGCACCGGCTAACTTCGTGCCAGCAGCCGCGGTAATACGAAGGGTGCAAGCGTTACTCGGAATTACTGGGCGTAAAGCGTGCGTAGGTGGTTTGTTAAGTCTGATGTGAAAGCCCTGGGCTCAACCTGGGAATGGCACTGGATACTGGCGAGCTAGAGTG	1	Uncultured bacterium clone	1.00E-142	98%	-	-	-
				Uncultured Luteimonas sp.	1.00E-141	97%	Proteobacteria	Xanthomonadaceae	Luteimonas
				Luteimonas sp.	2.00E-139	97%	Proteobacteria	Xanthomonadaceae	Luteimonas
Oligotype 53	GAGGTCACTCCTGC	GGGGAATATTGGACAATGGGCGCAAGCCTGATCCAGCC	1	Uncultured bacterium clone	8.00E-149	99%	-	-	-

	AGACTCACTAG	ATACCGCGTGGGTGAAGAAGGCCTTCGGGTTGTAAAGCCCTTTTGTTGGGAAAGAAATCCTGCAGGTTAATAACCAGTAGGGATGACGGTACCCAAAGAAATAAGCACCGGCTAACTTCGTGCCAGCAGCCGCGGTAATACGAAGGGTGCAAGCGTTACTCGGAATTACTGGGCGTAAAGCGTGCGTAGGTGGTTGTTTAAGTCTGCTGTGAAAGCCCCGGGCTCAACCTGGGAATTGCAGTGGATACTGGGCGACTAGAGT		Pseudoxanthomonas sp.	8.00E-149	99%	Proteobacteria	Xanthomonadaceae	Pseudoxanthomonas
Oligotype 54	GAGGTATTATCCTTGCATAGCGACG	TGGGGAATATTGGACAATGGGCGCAAGCCTGATCCAGCCATACCGCGTGGGTGAAGAAGGCCTTCGGGTTGTAAAGCCCTTTTGTTGGGAAGAAATCCTGCAGGTTAATAACCAGTAGGGATGACGGTACCCAAAGAATAAGCACCGGCTAACTTCGTGCCAGCAGCCGCGGTAATACGAAGGGGTGCAAGTCGTTACTCGGAAATTACTGGGCGTAAAGCGTGCGTAGGTGGTTGTTTAAGTCTGCTGTGAAAGCCCCGGGCTCAACCTGGGAATTGCAGTGGATACTGGGCGACTAGA	1	Uncultured bacterium clone	8.00E-144	98%	-	-	-
				Pseudoxanthomonas sp.	8.00E-144	98%	Proteobacteria	Xanthomonadaceae	Pseudoxanthomonas
				Xanthomonas sp.	8.00E-144	98%	Proteobacteria	Xanthomonadaceae	Xanthomonas
Oligotype 55	GAGGGCACTCCTGCAAACTTGCTAG	TGGGGAATATTGGACAATGGGCGCAAGCCTGATCCAGCCATACCGCGTGGGTGAAGAGGGCCTTCGGGTTGTAAAGCCCTTTTGTTGGGAAGAAATCCTGCAGGTTAATAACCAGTAGGGAGTGACGGTACCCAAAGAATAAGCACCGGCTAACTTCGTGCCAGCAGCCGCGGTAATACGAAGGGTGCAAGCGTTACTCGGAATTACTGGGCGTAAAGCGTGCGTAGGTGGTTGTTTAAGTCTGCTGTGAAAGCCCTGGGCTCAACCTGGGAATGGCAATGGATAC	1	Uncultured bacterium clone	3.00E-138	98%	-	-	-
				Uncultured Xanthomonas sp.	2.00E-140	97%			

		TGGGTAGCTAGAGT							
Oligotype 56	GAGGACACTCCTTGCAATAGCGACG	TGGGGAATATTGGACAATGGGCGCAAGCCTGATCCAGCCATACCGCGTGGGTGAAGAAGGCCTTCGGGTTGTAAAGCCCTTTTGTTGGGAAGAAATCCTGCAGGTTAATAACCAGTAGGGGATGACGGTACCCAAAGAATAAGCACCGGCTAACTTCGTGCCAGCAGCCGCGGTAATACGAAGGGTGCAAGCGTTACTCGGAATTACTGGGCGTAAAGCGTGCGTAGGTGGTTGTTTAAGTCTGCTGTGAAAGCCCCGGGCTCAACCTGGGAAATTGCAGTAGGATACTGGGCGACTAGA	1	Uncultured bacterium clone	8.00E-144	98%	-	-	-
				Pseudoxanthomonas sp.	8.00E-144	98%	Proteobacteria	Xanthomonadaceae	Pseudoxanthomonas
Oligotype 57	CTCGGTTTGGTCTGCATACGGACTA	TGGGGAATATTGGACAATGGGCGCAAGCCTGATCCAGCCATACCGCGTGGGTGAAGAAGGCCTTCGGGTTGTAAAGCCCTTTTGTTGGGTAAAGAAATACCTGCAGGTTAATAACCAGTAGGGATGACGGTACCCAAAGAATAAGCACCGGCTAACTTCGTGCCAGCAGCCGCGGTAATACGAAGGGTGCAAGCGTTACTCGGAATTACTGGGCGTAAAGCGTGCGTAGGTGGTTTTTAAGTCTGCTGTGAAAGCCCCGGGCTCAACCTGGGAATTGCAGTGGATACTGGGCGACTAGAG	1	Uncultured bacterium clone	2.00E-145	98%	-	-	-
				Pseudoxanthomonas sp.	2.00E-145	98%	Proteobacteria	Xanthomonadaceae	Pseudoxanthomonas
Oligotype 58	CTCGGTATACGAATTGGATGGCGAA	TGGGGAATATTGGACAATGGGCGCAAGCCTGATCCAGCCATACCGCGTGGGTGAAGAAGGCCTTCGGGTTGTAAAGCCCTTTTCGTTGGGTAAGAAATACCTGCAGGTTAATAACCAGTAGGGAGTGACGGTACCCAAAGAATAAGCACCGGCTAACTTCGTGCCAGCAGCCGCGGTAATACGAAGGGTGCAAGCGTTACTCGGAATTA	1	Uncultured bacterium clone	3.00E-143	98%	-	-	-
				Pseudoxanthomonas sp.	3.00E-143	98%	Proteobacteria	Xanthomonadaceae	Pseudoxanthomonas

		CTGGGCGTAAAGCGTGCGT AGGTGGTTGTTTAAGTCTG CTGTGAAAGCCCCGGGCTC AACCTGGGAATTGCAGTGG ATACTGGGCGACTAG							
Oligotype 59	CTCGGTA TACGAAG GCGATGT ATGT	TGGGGAATATTGGACAATG GGCGCAAGCCTGATCCAGC CATACCGCGTGGGTGAAGA AGGCCTTCGGGTTGTAAAG CCCGTTTTGTTGGGAAAGA AATACCTGCAGGTTAATAA CCAGTAGGGAGTGACGGTA CCCAAAGAATAAGCACCGG CTAACTTCGTGCCAGCAGC CGCGGTAATACGAAGGGTG CAAGCGTTACTCGGAATTA CTGGGCGTAAAGCGTGCGT AGGTGGTTGTTTAAGTCTG CTGTGAAAGCCCCGGGCTC AACCCAGAGAGGCCACTCG ATACTGCTATGACTA	1	Uncultured bacterium clone	3.00E-128	98%	-	-	-
				Pseudoxanthomo nas sp.	3.00E-128	98%	Proteoba cteria	Xanthomona daceae	Pseudoxantho monas
Oligotype 60	CGTGGTC TCTCTTGC ATACGGA CTA	TGGGGAATATTGGACAATG GGCGCAAGCCTGATCCAGC CATACCGCGTGGGTGAAGA AGGCCTTCGGTTGTAAGCC CTTTTGTTGGGAAAGAAAT CCTGCAGGTTAATAACCAG TAGGGATGACGGTACCCAA AGAATAAGCACCGGCTAAC TTCGTGCCAGCAGCCGCGG TAATACGAAGGGTGCAAGC GTTAACTCGGAATTACTGG GCGTAAAGCGTGCGTAGGT GGTTGTTTAAGTCTGCTGT GAAAGCCCCCGGGCTCAAC CTGGGGAATTGCAGTGGAT ACTGGGCGACTAGAG	1	Uncultured bacterium clone	4.00E-142	98%	-	-	-
				Pseudoxanthomo nas sp.	4.00E-142	98%	Proteoba cteria	Xanthomona daceae	Pseudoxantho monas
Oligotype 61	CGTGGGG GGTCGGT GGGGGCA GAGT	GGGGGAATATTGGACAATG GGCGCAAGCCTGATCCAGC CATACCGCGTGGGTGAAGA AGGCCTTCGGGTTGTAAGC CCTTTTGTTGGGAAGAAAT CCTGCAGGTTAATAACCAG TAGGGATGACGGTACCCAA	1	Uncultured bacterium clone	1.00E-146	98%	-	-	-
				Pseudoxanthomo nas sp.	5.00E-146	98%	Proteoba cteria	Xanthomona daceae	Pseudoxantho monas
				Pseudomonas sp.	5.00E-146	98%	Proteoba cteria	Pseudomona daceae	Pseudomonas
				Xanthomonas sp.	5.00E-146	98%	Proteoba	Xanthomona	Xanthomonas

		GAATAAGCACCGGCTAACT TCGTGCCAGCAGCCGCGGT AATACGAAGGGTGCAAGC GTTACTCGGAATTACTGGG CGTAAAGCGTGCGTAGGTG GTTGTTTAAGTCTGCTGTG AAAGCCCCGGGCTCAACCT GGGAATTGCAGTGGATACT GGGCGACTAGAGTGTG					cteria	daceae	
Oligotype 62	CGTGGCT GTTATCA CGCTGGC TCGG	TGGGGAATATTGGACAATG GGCGCAAGCCTGATCCAGC CATACCGCGTGGGTGAAGA AGGCCTTCGGGTTGTAAAG CCCTTTTGTTGGGAAGAAT CCTGCAGGTTAATAACCAG TAGGGATGACGGTACCCAA AGAATAAGCACCGGCTAAC TTCGTGCCAGCAGCCGCGG TAATACGAAGGGTGCAAGC GTTACTCGGAATTACTGGG CTTAAAGGGTGCGTAGGCT GGCCCATAAGTGTCCGTGT GAAAGCCCACTGCTCAACG GTGGAACAGCACGGCAAA CTGTGGGTCTCGAGGA	1	Uncultured Xanthomonas sp.	4.00E-107	98%	Proteoba cteria	Xanthomona daceae	Xanthomonas
				Pseudoxanthomo nas sp.	2.00E-105	97%	Proteoba cteria	Xanthomona daceae	Pseudoxantho monas
				Stenotrophomona s sp.	2.00E-105	97%	Proteoba cteria	Xanthomona daceae	Stenotrophom onas
Oligotype 63	CGGGTTC TCTAGCA TTCTGACT AGT	TGGGGAATATTGGACAATG GGCGCAAGCCTGATCCAGC CATGCCGCGTGTGTGAAGA AGGCCTTCGGTTGTAAAGC ACTTTTGTCCGGGAAGAAA GACACCGGGTCAATACCCT GGTGTCATGACGGTACCGG AAGAATAAGCACCGGCTAA CTTCGTGCCAGCAGCCGCG GTAATACGAAGGGTGCAAG CGTTACTCGGAATTACTGG GCGTAAAGCGTGCGTAGGC GGTTTGTTAAGTCTGATGT GAAAGCCCTGGGCTCAACC TGGGAATGGCATTGGATAC TGGCGATCTAGAATG	1	Uncultured bacterium clone	6.00E-140	98%	-	-	-
				Lysobacter sp.	1.00E-142	98%	Proteoba cteria	Xanthomona daceae	Lysobacter
Oligotype 64	CGCTGAT AGGCCTT GCATAGG	TGGGGAATTTTTGGACAAT GGGCGCAAGCCTGATCCAG CCATGCCGCGTGGGTGAAG	1	Uncultured bacterium clone	4.00E-137	98%	-	-	-
				Pseudoxanthomo	4.00E-137	98%	Proteoba	Xanthomona	Pseudoxantho

	GGCG	AAGGCCTTCGGGTTGTAAA GCCCTTTTGTTGGGAAAGA AATCCTGCAGGTTAATAAC CAGTAGGGATGACGGTACC CAAAGAATAAGCACCGGCT AACTTCGTGCCAGCAGCCG CGGTAATACGAAGGGTGCA AGCGTTACTCGGAATTACT GGGCGTAAAGCGTGCGTAG GTGGTTGTTTAAGTCTGCT GTGAAAGCCCCCGGGCTCA ACCTGGGAATTGCGGTGGA TACTGGCGGGCTAGA		nas sp.			cteria	daceae	monas
				Pseudomonas sp.	4.00E-137	98%	Proteobacteria	Pseudomonadaceae	Pseudomonas
Oligotype 65	CGAGGCT TTAGTGG TACAGAT TTCG	GGGGAATCTTGCACAATGG GCGAAACGCCTGATGCAGC AACGCCGCGTGCGGAAGA AGGCCCTAGGGTTGTAAAC CGCTTTCAGTAGGGAAGAA AATGACGGTACCTACAGAA GAAGGTGCGGCCAACTACG TGCCAGCAGCCGCGGTGAC ACGTAGGCACCAAGCGTTA TCCGGATTTATTGGGCGTA AAGCGTGCGTAGGTGGTTG TTTAAGTCTGCTGTGAAAG CCCCGGCTCAACCTGGGAA TTGCAGTGGATACTGGGCG ACTAGAGTGTGGTAGAGGA CAGTGGAATTTCCGGT	1	Uncultured bacterium clone	1.00E-97	94%	-	-	-
Oligotype 66	CGACGGG TCGGAGT GGGACGA ATTC	TGGGGAATCTTGGACAATG GGGGCAACCCTGATCCAGC CATGCCGCGTGGTGATGAA GGTCTTCGGATTGTAAAAC CCTTTTGGCGGGGACGATA ATGACGGTACCCGCAGAAT AAGCACCGGCTAACTTCGT GCCAGCAGCCGCGGTAATA CGAAGGGTGCAAGCGTTAC TCGGAATTACGTGGGCGTA AAGCGTGCGTAGGTGGTTG TTTAAGTCTGCTGTGAAAG CCCCGGGCTCAACCTGGGA ATTGCAGTGGATACTGGGC GACTAGAGTGTGGTAGAGG ACAGTGGAATTTCCG	1	Uncultured bacterium clone	2.00E-124	94%	-	-	-

Oligotype 67	CCGTTTCTCTAGCAGTCTGGCTAGT	TGGGGAATTTTGGACAATGGGCGCAAGCCTGATCCAGCCATGCCGCGTGTGTGAAGAAGGCCTTCGGGTTGTAAAGCACTTTCGGCCGGAACGAAATCGCGCGGGCTATTACCCGCGTGGATGACGGTACCGGAAGAAGAAGCACCGGCTAACTTCGTGCCAGCAGCCGCGGTAATACGAAGGGTGCAAGCGTTACTCGGAATTACTGGGCGTAAAGCGTGCGTAGGTGGTTGTTTAAGTCTGCTGTGAAAGCCCCGGGCTCAACCTGGGAATTGCAGTGGATACTGGGCGACTAGAGTG	1	Lysobacter sp.	1.00E-117	94%	Proteobacteria	Xanthomonadaceae	Lysobacter
				Uncultured bacterium clone	1.00E-122	94%	-	-	-
Oligotype 68	CCCGAATAGGTCTGCATACGGCGAA	TGGGGAATTTTGGACAATGGGCGCAAGCCTGATCCAGCCATGCCGCGTGTGTGAAGAAGGCCTTCGGGTTGTAAAGCACTTTCGGCCGGAACGAAATCGCGCGGGCTATTACCCCGCGTGGATGACGGTACCGGAAGAAGAAGCACCGGCTAACTTCGTGCCAGCAGCCGCGGTAATACGAAGGGTGCAAGCGTTTACTCGGAATTACTGGGCGTAAAGCGTGCGTAGGTGGTTGTTTAAGTCTGCTGTGAAAGCCCCGGGCTCAACCTGGGAATTGCAGTGGATACCTGGGGCGACTAG	1	Uncultured beta proteobacterium clone	3.00E-113	94%	Proteobacteria	-	-
				Lysobacter sp.	2.00E-115	93%	Proteobacteria	Xanthomonadaceae	Lysobacter
				Arenimonas sp.	3.00E-113	92%	Proteobacteria	Xanthomonadaceae	Arenimonas
Oligotype 69	CATTGATAGGCTGCAAACTTGCTAG	TGGGGAATATTGGACAATGGGGGCAACCCTGATCCAGCAATGCCGCGTGTGTGAAAGAAGGCCTTCGGGTTGTAAAGCACTTTTATTGGGAACGAAAAGCGATGGGTTAATACCTTATCGTGCTGACGGTACCCAAGGAATAAGCACCGGCTAACTTCGTGCCAGCAGCCGCGGTAATACGAAGGGTGCAAGCGTTAATCGGAATTACTGGGCGTAAAGGGTGCGTAGGCGGTTACTTAAGTCTGTT	1	Uncultured bacterium clone	4.00E-127	99%	-	-	-
				Uncultured Rhodanobacter sp.	1.00E-122	94%	Proteobacteria	Xanthomonadaceae	Rhodanobacter

		GTGAAGCCCTGGGCTCAAC CTGGGAATGGCAATGGATA CTGGGTAGCTAGAGT							
Oligotype 70	ATTTCAAT CTAGCAG GCTGGCT AGT	TGGGGAATATTGGACAATG GGGGCAACCCTGATCCAGC AATGCCGCGTGTGTGAAGA AGGCCTGCGGGTTGTAAAG CACTTTTATTCGGAACGAA AAGCAATGGATTAACACTC TGTTGTGCTGACGGTACCG GAAGAATAAGCACCGGCTA ACTTCGTGCCAGCAGCCGC GGTAATACGAAGGGTGCAA GCGTTAATCGGAATTATTG GGCGTAAAGCGTGCGTAGG TGGTTTTTAAGTCTTTTGTG AAAGCCCTGGGCTCAACCT GGGAACTGCAGGAGAAAC TGGAAGGCTAGAGTC	1	Uncultured bacterium clone	2.00E-130	95%	-	-	-
				Uncultured Xanthomonas sp.	2.00E-110	94%	Proteoba cteria	Xanthomona daceae	Xanthomonas
				Lysobacter sp.	4.00E-112	92%	Proteoba cteria	Xanthomona daceae	Lysobacter
Oligotype 71	AGTGCGG TCGTGTGT GGGATGA ATC	TGGGGAATCTTGCACAATG GGCGAAAGCCTGATGCAGC AACGCCGCGTGCGGGAAG AAGGCCCTAGGGTTGTAAA CCGCTTTCAGTAGGGAAGA AAATGACGGTACCCAAAGA ATAAGCACCGGCTAACTTC GTGCCAGCAGCCGCGGTAA TACGAAGGGTGCAAGCGTT ACTCGGAATTACTGGGCGT AAAGCGTGCGTAGGTGGTT GTTTAAGTCTGCTGTGAAA GCCCCGGGCTCAACCTGGG GAATTGCAGTGGATACTGG GCGACTAGAGTGTGGTAGA GGACAGTGGAATTTCC	1	Uncultured bacterium clone	4.00E-97	99%	-	-	-
				Pseudoxanthomo nas sp.	4.00E-97	99%	Proteoba cteria	Xanthomona daceae	Pseudoxantho monas
				Pseudomonas sp.	2.00E-95	99%	Proteoba cteria	Pseudomona daceae	Pseudomonas
Oligotype 72	AGTGCGG TCGGAGT GGGACGA ATTC	TGGGGAATTTTCCGCAATG GGCGAAAGCCTGACGGAG CAATACCGCGTGAGGGACG AAGGTCTGTGGATTGTAAA CCTCTTTTGTTGGGGAAGA TAATGACGGTACCCAACGA ATAAGCATCGGCTAACTCC GTGCCAGCAGCCGCGGTAA GACGGAGGATGCAAGCGTT	1	Uncultured bacterium clone	4.00E-107	91%	-	-	-

		ATCCGGAATTACTGGGCGT AAAGCGTGCGTAGGTGGTT GTTTAAGTCTGCTGTGAAA GCCCCGGGCTCAACCTGGG AATTGCAGTGGATACTGGG CGACTAGAGTGTGGTAGAG GACAGTGGAATTTCCG							
Oligotype 73	AGGGCGG TCGTGTGT GGGATGA ATC	TGGGGAATATTGGACAATG GGCGCAAGCCTGATCCAGC AATTCCGCGTGGGTGAAGA AGGTCTTCGGATTGTAAAG CCCTTTCGTTGGGGACGAT GATGACGGTACCCAAAGAA GAAGCCCCGGCTAACTTCG TGCCAGCAGCCGCGGTAAT ACGAAGGGTGCAAGCGTTA CTCGGAATTACTGGGCGTA AAGCGTGCGTAGGTGGTTG TTTAAGTCTGCTGTGAAAG CCCCGGGCTCAACCTGGGG AATTGCAGTGGATACTGGG CGACTAGAGTGTGGTAGAG GACAGTGGAATTTCC	1	Uncultured bacterium clone	8.00E-124	94%	-	-	-
				Uncultured Thiothrix sp.	1.00E-102	90%	Proteobacteria	Thiotrichaceae	Thiothrix
Oligotype 74	AGGGCGG TCGGGGC GGGGGGA ATTC	TGGGGAATATTGGACAATG GGCGCAAGCCTGATCCAGC AATGCCGCGTGGATGAAGA AGGTCTTCGGATTGTAAAG TCCTTTCGACGGGGACGAT GATGACGGTACCCGTAGAA GAAGCCCCGGCTAACTTCG TGCCAGCAGCCGCGGTAAT ACGAAGGGGGCTAGCGTTA CTCGGAATTACTGGGCGTA AAGCGTGCGTAGGCGGTTT GTTAAGTCCGATGTGAAAG CCCTGGGCTCAACCTGGGA ATGGCATGGGATACTGGCG AGCTAGAGTGCGGTAGAGG GGTGCGGAATTCCCG	1	Uncultured bacterium clone	2.00E-124	94%	-	-	-
				Uncultured Gluconobacter sp.	2.00E-110	91%	Proteobacteria	Acetobacteraceae	Gluconobacter
				Roseomonas sp.	2.00E-110	91%	Proteobacteria	Acetobacteraceae	Roseomonas
Oligotype 75	AGGACGG TCGGAGT GGGACGA ATTC	TGGGGAATCTTAGACAATG GGCGCAAGCCTGATCTAGC CATGCCGCGTGAGTGATGA AGGCCTTAGGGTCGTAAAG CTCTTTCGCTGGGGAAGAT	1	Uncultured bacterium clone	1.00E-117	93%	-	-	-

		AATGACGGTACCCAGTAAA GAAACCCCGGCTAACTTCG TGCCAGCAGCCGCGGTAAT ACGAAGGGTGCAAGCGTTA CTCGGAATTACTGGGCGTA AAGCGTGCGTAGGTGGTTG TTTAAGTCTGCTGTGAAAG CCCCGGGCTCAACCTGGGA ATTGCAGTGGATACTGGGC GACTAGAGTGTGGTAGAGG ACAGTGGAATTTCCG							
Oligotype 76	AGAGTTC TCTAGCA ATCTGAC TAGT	TGGGGAATATTGGACAATG GGGGCAACCCTGATCCAGC AATGCCGCGTGTGTGAAGA AGGCCTTCGGGTTGTAAAG CACTTTTATTGGGAACGAA AGCGATGGGTTAATACCTT ATCGTGCTGACGGTACCCA AGGAATAAGCACCGGCTAA CTTCGTGCCAGCAGCCGCG GTAATACGAAGGGTGCAAG CGTTAATCGGAATTACTGG GCGTAAAGGGTGCGTAGGC GGTTACTTAAGTCTGTTGT GAAAGCCCTGGGCTCAACC TGGGAATGGCAATGGATAC TGGGTAGCTAGAGTA	1	Uncultured bacterium clone	8.00E-129	99%	-	-	-
				Uncultured Rhodanobacter sp.	2.00E-125	94%	Proteoba cteria	Xanthomona daceae	Rhodanobacte r
				Dyella sp.	4.00E-122	94%	Proteoba cteria	Xanthomona daceae	Dyella
Oligotype 77	AGAGTTC TCTAGAA TGTGGGT AGAA	GGGGGAATATTGGACAATG GGGGCAACCCTGATCCAGC AATGCCGCGTGTGTGAAGA AGGCCTTCTGGTTGTAAGC ACTTTTATTGGGAACGAAA AGCGATGGGTTAATATCTT ATCGTGCTGACGGTACCCA AGGAATAAGCACCGGCTAA CTTCGTGCCAGCAGCCGCG GTAATACGAAGGGTGCAAG CGTTAATCGGAATTACTGG GCGTAAAGGGTGCGTAGGC GGTTACTTAAGTCTGTTGT GAAAGCCCTGGCTCAACCT GGGAATGGCAATGGATACT GGGTAGCTAGAGTAC	1	Uncultured bacterium clone	2.00E-124	98%	-	-	-
				Uncultured Rhodanobacter sp.	3.00E-118	93%	Proteoba cteria	Xanthomona daceae	Rhodanobacte r
Oligotype	AGAGGGG	TGGGGAATATTGGACAATG	1	Uncultured	6.00E-140	97%	-	-	-

78	GGTCGGT GGGGGCA GAGT	GGCGCAAGCCTGATCCAGC CATACCGCGTGGGTGAAGA AGGCCTTCGGGTTGTAAGC CCTTTGTTGGGAAGAATAC CTGCAGGTTAATAACCAGT AGGGAGTGACGGTACCCAA GAATAAGCACCGGCTAACT TCGTGCCAGCAGCCGCGGT AATACGAAGGGTGCAAGC GTTACTCGGAATTACTGGG CGTAAAGCGTGCGTAGGTG GTTGTTTAAGTCTGCTGTG AAAGCCCCGGGCTCAACCT GGGAATTGCAGTGGATACT GGGCGACTAGAGTGTG		bacterium clone					
				Pseudoxanthomo nas sp.	6.00E-140	97%	Proteoba cteria	Xanthomona daceae	Pseudoxantho monas
Oligotype 79	AGAGAGG GGTTAGA TACGAGG TGTT	TGGGGAATATTGGACAATG GGCGCAAGCCTGATCCAGC CATACCGCGTGGGTGAAGA AGGCCTTCGGGTTGTAAGC CCTTTGTTGGGAAGAAATC CTGCAGGTTAATAACCAGT AGGGATGACGGTACCCAAA GAATAAGCACCGGCTAACT TCGTGCCAGCAGCCGCGGT AATACGAAGGGTGCAAGC GTTACTCGGAATTACTGGG CGTAAAGCGTGCGTAGGTG GTTGTTTAAGTCTGCTGTG AAAGCCCGGCTCAACCTGG GAATGCAGTGGATACTGGG CGACTAGAGTGTGGTA	1	Uncultured bacterium clone	2.00E-144	98%	-	-	-
				Pseudoxanthomo nas sp.	2.00E-144	98%	Proteoba cteria	Xanthomona daceae	Pseudoxantho monas
Oligotype 80	AGAGAGG GGTCGGT GGGGGCA GAGT	TGGGGAATATTGGACAATG GGCGCAAGCCTGATCCAGC CATACCGCGTGGGTGAAGA AGGCCTTGGGTTGTAAGCC CTTTTGTTGGGAAGAAATC CTGCAGGTTAATAACCAGT AGGGATGACGGTACCCAAA GAATAAGCACCGGCTAACT TCGTGCCAGCAGCCGCGGT AATACGAAGGGTGCAAGC GTTACTCGGAATTACTGGG CGTAAAGCGTGCGTAGGTG GTTGTTTAAGTCTGCTGTG AAAGCCCCGGGCTCAACCT	1	Uncultured bacterium clone	1.00E-146	98%	-	-	-
				Pseudoxanthomo nas sp.	1.00E-146	98%	Proteoba cteria	Xanthomona daceae	Pseudoxantho monas

		GGGAATTGCAGTGGATACTGGGCGACTAGAGTGTG							
Oligotype 81	ACTTGTATACGAATGGGATGGTAGA	TGGGGAATATTGGACAAATGGGGGCAACCCTGATCCAGCAATGCCCGCGTGTGTGAAAGAAGGCCTTCGGGTTGTAAAGCACTTTTATTGGGAACGAAAAGCGATGGGTTAATATCTTATCGTGCTGACGGTACCCAAGGAATAAGCACCGGCTAACTTCGTGCCAGCAGCCGCGGTAATACGAAGGGTGCAAGCGTTAATCGGAATTACTGGGCGTAAAGGGTGCGTAGGCGGTTATTTAAGTCTGTTGTGAAAGCCCTGGGCTCAACCTGGGAATGGCAATGGATACTGGGTAGCTAG	1	Uncultured bacterium clone	5.00E-126	99%	-	-	-
				Uncultured Rhodanobacter sp.	8.00E-119	94%	Proteobacteria	Xanthomonadaceae	Rhodanobacter
Oligotype 82	ACTCTTTTGGGGTACTACTGGTGGG	TAGGGAATATTGGACAATGGGTGCGAGCCTGATCCAGCCATGCCGCGTGCAGGAAGACGGCCTTCTGGGTTGTAAACTGCTTTTACCAGGGGATAAAAGACCATGCGTGGGACATTGCCGGTACCAGATGAATAAGCACCGGCTAACTCCGTGCCAGCAGCCGCGGTAATACGGAGGGTGCAAGCGTTGTCCGGATTTATTGGGTTTAAAGGTGCGTAGGTGGTTGTTTAAGTCTGCTGTGAAAGCCCCGGGCTCAACCTGGGAATTGCAGTGGATACTGGGCGACTAGAGTGTGGTAGA	1	Uncultured bacterium clone	4.00E-97	93%	-	-	-
Oligotype 83	ACAAGTATACGAAATTGGATGGCGT	TGGGGAATATTGGACAATGGGCGCAAGCCTGATCCAGCCATACCGCGTGGGTGAAGAAGGCCTTCGGGTTGTAAAGACCCTTTTGTTTGGGAAAGAAATACCTGCAGGTTAATAACCAGTAGGGATGACGGTACCCAAAGAATAAGCACCGGCTAACTTCGTGCCAGCAGCCGCGGTAATACGAAGGGTG	1	Uncultured bacterium clone	3.00E-143	98%	-	-	-
				Pseudoxanthomonas sp.	3.00E-143	98%	Proteobacteria	Xanthomonadaceae	Pseudoxanthomonas

		CAAGCGTTACTCGGAATTACTGGGCGTAAAGCGTGCGTAGGTGGTTGTTTAAGTCTGCTGTGAAAGCCCCCGGGCTCAACCTGGGAATTGCAGTGGATACTGGGCGACTA							
Oligotype 84	AAACGGGGTACCGGGAAGTATGGCC	TGGGGAATATTGGACAATGGGCGCAAGCCTGATCCAGCCAATGCCGCGTAGTGTGAAGAAGGCCTTCGGGTTGTAAAGACACTTTTGTCCGGGAAGAAAAGACACCGGGTCAATACCCTCGGTGTCATGACGGTACCGGAAGAATAAGCACCGGCTAACTTCGTGCCAGCAGCCGCGGTAATACGAAGGGTGCAAAGCGTTACTCGGAATTACTGGGCGTAAAGCGTGCGTAGGTGGTTGTTTAAGTCTGCTGTAGAAAGCCCCGGGCTCAACCTGGGAATTGCAGTGATACTGGCGACT	1	Uncultured bacterium clone	3.00E-143	98%	-	-	-
				Lysobacter sp.	4.00E-122	94%	Proteobacteria	Xanthomonadaceae	Lysobacter

Supplementary Table 3: Elaborated PICRUSt results at KEGG level 2 found in all three laminae (U1, U2, and U3) of the conical microbial mat from Lake Untersee, Antarctica.

	**Relative frequency (%)**		
**Level2**	**U1**	**U2**	**U3**
Energy metabolism	11.89138	8.199587	7.1746
Carbohydrate Metabolism	9.296634	11.36183	11.68234
Genetic Information Processing	3.322108	2.549536	2.481704
Amino Acid Metabolism	10.52104	11.58771	11.72825
Cellular Processes and Signaling	3.481018	3.335685	3.58969
Folding, Sorting, and Degradation	3.469229	2.876053	2.736253
Replication and Repair	7.203122	7.66769	7.8705

Transport and Catabolism	0.126109	0.276976	0.321507
Translation	5.153554	4.897501	4.854273
Transcription	1.369528	2.283908	2.481662
Metabolism of Cofactors and Vitamins	6.569245	5.511531	5.072587
Signal Transduction	2.012908	2.060833	2.124958
Glycan Biosynthesis and Metabolism	2.099122	1.867456	2.004959
Signaling Molecules and Interaction	0.12071	0.176034	0.188935
Cell Motility	1.666703	1.750925	1.995287
Enzyme Families	3.223916	2.437874	2.282523
Membrane Transport	9.913693	12.15913	12.56327
Metabolism of Other Amino Acids	2.131889	1.999419	1.932856
Cell Growth and Death	0.787679	0.572525	0.531145
Poorly Characterized	5.090673	5.179542	5.303082
Nucleotide Metabolism	3.874769	3.714677	3.732172
Biosynthesis of Other Secondary Metabolites	1.340206	1.297615	1.306199
Lipid Metabolism	2.965564	3.669127	3.66454
Metabolism of Terpenoids and Polyketides	2.369206	2.566839	2.376703

Supplementary Table 4: Summarized PICRUSt results in (a) cyanobacteria and (b) heterotrophs at KEGG level 3 found in all three laminae (U1, U2, and U3) of the conical microbial mat from Lake Untersee, Antarctica. The results also have been presented in a bar graph format in Supplementary Figure 3.

Supplementary Table 4(a)

**KEGG LEVEL 3**	**U1-Cyanobacteria**	**U2C-Cyanobacteria**	**U3C-Cyanobacteria**
**Amino Acid Metabolism**			
Alanine, aspartate and glutamate metabolism	84082	27076	15129
Amino acid related enzymes	107383	34548	19305
Arginine and proline metabolism	145654	46000	25708
Cysteine and methionine metabolism	109531	33430	18805

Glycine, serine and threonine metabolism	69197	21786	12190
Histidine metabolism	58891	18356	10245
Lysine biosynthesis	50210	16110	9055
Lysine degradation	16310	5112	2839
Phenylalanine metabolism	35273	10682	5870
Phenylalanine, tyrosine and tryptophan biosynthesis	78877	24780	13937
Tryptophan metabolism	29273	9066	5014
Tyrosine metabolism	54930	16872	9373
Valine, leucine and isoleucine biosynthesis	69863	22302	12520
Valine, leucine and isoleucine degradation	20608	6904	3852
Total	930082	293024	163842
**Carbohydrate Metabolism**			
Amino sugar and nucleotide sugar metabolism	140750	43886	24563
Butanoate metabolism	33912	11142	6206
C5-Branched dibasic acid metabolism	32303	9928	5603
Citrate cycle (TCA cycle)	45611	14206	7995
Fructose and mannose metabolism	64469	20986	11796
Galactose metabolism	32648	10226	5820
Glycolysis / Gluconeogenesis	101692	33090	18572
Glyoxylate and dicarboxylate metabolism	46586	14908	8448
Inositol phosphate metabolism	9985	3204	1832
Pentose and glucuronate interconversions	25994	8102	4624
Pentose phosphate pathway	86747	27630	15311
Propanoate metabolism	36601	11746	6616
Pyruvate metabolism	91137	29640	16826
Starch and sucrose metabolism	61880	20640	11840
Ascorbate and aldarate metabolism	9680	3020	1747
Total	819995	262354	147799
**Cell Motility**			
Bacterial chemotaxis	22009	6528	3772

Bacterial motility proteins	101720	33084	18838
Total	123729	39612	22610
**Energy Metabolism**			
Carbon fixation in photosynthetic organisms	70196	22526	12747
Carbon fixation pathways in prokaryotes	69851	22272	12530
Methane metabolism	88412	28914	16322
Nitrogen metabolism	76116	23922	13463
Oxidative phosphorylation	146994	47042	26568
Photosynthesis	201875	65224	36747
Photosynthesis proteins	286070	91070	51058
Photosynthesis - antenna proteins	80868	24786	13713
Sulfur metabolism	39579	12448	6959
Total	1059961	338204	190107
**Lipid Metabolism**			
Biosynthesis of unsaturated fatty acids	23871	7718	4214
Fatty acid biosynthesis	46562	14820	8344
Lipid biosynthesis proteins	64425	20884	11626
Fatty acid metabolism	27599	9294	5189
Total	162457	52716	29373
**Membrane Transport**			
ABC transporters	278664	91388	51630
Bacterial secretion system	37544	12316	6889
Transporters	445880	146472	82467
Phosphotransferase system (PTS)	0	2	0
Total	762088	250178	140986
**Metabolism of Cofactors and Vitamins**			
Pantothenate and CoA biosynthesis	50579	16468	9376
Porphyrin and chlorophyll metabolism	197208	63166	35413
Riboflavin metabolism	33262	10608	5904
Thiamine metabolism	45936	14410	8146

Total	326985	104652	58839
**Nucleotide Metabolism**			
Purine metabolism	213109	67762	37923
Pyrimidine metabolism	130323	41650	23360
Total	343432	109412	61283
**Enzyme Families**			
Peptidases	206892	66188	37198
Protein kinases	79824	25344	14496
Cyanophycin	3335	1077	656
Total	286716	91532	51694
**Signal Transduction**			
Two-component system	161549	53536	30704
Total	161549	53536	30704
**Replication and Repair**			
DNA repair and recombination proteins	202304	66702	37337
Total	202304	66702	37337
**Signaling Molecules and Interaction**			
Bacterial toxins	3652	1300	749
Total	3652	1300	749
**Translation**			
Ribosome	182981	58400	32852
Total	182981	58400	32852

Supplementary Table 4(b)

**KEGG LEVEL 3**	**U1-Heterotrophs**	**U2H-Heterotrophs**	**U3H-Heterotrophs**
**Amino Acid Metabolism**			
Alanine, aspartate and glutamate metabolism	1129	52453	59666
Amino acid related enzymes	1255	63686	74792

Arginine and proline metabolism	1511	68456	82019
Cysteine and methionine metabolism	1017	42172	52336
Glycine, serine and threonine metabolism	992	48594	56556
Histidine metabolism	657	32087	36575
Lysine biosynthesis	596	30637	35626
Lysine degradation	537	28369	27445
Phenylalanine metabolism	523	21176	25329
Phenylalanine, tyrosine and tryptophan biosynthesis	726	37009	44482
Tryptophan metabolism	787	37148	39417
Tyrosine metabolism	627	26674	31087
Valine, leucine and isoleucine biosynthesis	878	42724	48680
Valine, leucine and isoleucine degradation	928	51730	49952
Total	12163	582915	663962
**Carbohydrate Metabolism**			
Amino sugar and nucleotide sugar metabolism	1230	56891	68524
Butanoate metabolism	1172	58429	58924
C5-Branched dibasic acid metabolism	324	16491	18871
Citrate cycle (TCA cycle)	1095	49547	56033
Fructose and mannose metabolism	778	33836	40410
Galactose metabolism	594	25776	31202
Glycolysis / Gluconeogenesis	1473	63919	72464
Glyoxylate and dicarboxylate metabolism	843	43440	46540
Inositol phosphate metabolism	243	13036	13062
Pentose and glucuronate interconversions	722	30185	39706
Pentose phosphate pathway	956	41705	50490
Propanoate metabolism	925	52898	51928
Pyruvate metabolism	1226	63260	70535
Starch and sucrose metabolism	786	37930	47247
Ascorbate and aldarate metabolism	188	9056	10649
Total	12555	596399	676585

**Cell Motility**			
Bacterial chemotaxis	173	19357	24553
Bacterial motility proteins	296	40943	56238
Total	469	60300	80791
**Energy Metabolism**			
Carbon fixation in photosynthetic organisms	464	23765	27292
Carbon fixation pathways in prokaryotes	875	51652	56604
Methane metabolism	988	52560	56797
Nitrogen metabolism	719	37422	44790
Oxidative phosphorylation	1123	71165	79321
Photosynthesis	269	14563	16752
Photosynthesis proteins	270	14843	17155
Photosynthesis - antenna proteins	0	0	0
Sulfur metabolism	236	16269	18119
Total	4944	282239	316830
**Lipid Metabolism**			
Biosynthesis of unsaturated fatty acids	289	14090	16571
Fatty acid biosynthesis	449	28080	31664
Lipid biosynthesis proteins	727	40781	44637
Fatty acid metabolism	764	41580	38507
Total	2229	124531	131379
**Membrane Transport**			
ABC transporters	4895	206211	225819
Zinc transport system	14	224	298
Bacterial secretion system	552	27719	37043
Transporters	8488	342786	388935
Phosphotransferase system (PTS)	192	5319	7113
Arsenical resistance protein	0	30	120
Arsenical pump membrane protein	0	80	134
Total	14127	582035	658910

**Metabolism of Cofactors and Vitamins**			
Pantothenate and CoA biosynthesis	609	31682	35625
Porphyrin and chlorophyll metabolism	610	46988	45947
Riboflavin metabolism	298	15390	16610
Thiamine metabolism	367	18921	19857
Total	1884	112981	118039
**Nucleotide Metabolism**			
Purine metabolism	2048	97858	115534
Pyrimidine metabolism	1517	73630	86846
Total	3565	171488	202380
**Enzyme Families**			
Peptidases	1528	72947	87331
Protein kinases	467	20028	22347
Total	1995	92975	109678
**Signal Transduction**			
Two-component system	1598	88468	106623
Total	1598	88468	106623
**Replication and Repair**			
DNA repair and recombination proteins	2738	126356	149637
Total	2738	126356	149637
**Signaling Molecules and Interaction**			
Bacterial toxins	77	4389	4759
Total	77	4389	4759
**Translation**			
Ribosome	1896	95642	109506
Total	1896	95642	109506
