# Supplementary material for: Microbial Communities and Their Predicted Metabolic Functions in Growth Laminae of a Unique Large Conical Mat from Lake Untersee, East Antarctica
Source: Front Microbiol. 2017 Aug 4;8:1347. doi: 10.3389/fmicb.2017.01347 (PMC5543034; doi:10.3389/fmicb.2017.01347)
Supplement: Supplementary file 2 [file Image_1.pdf]

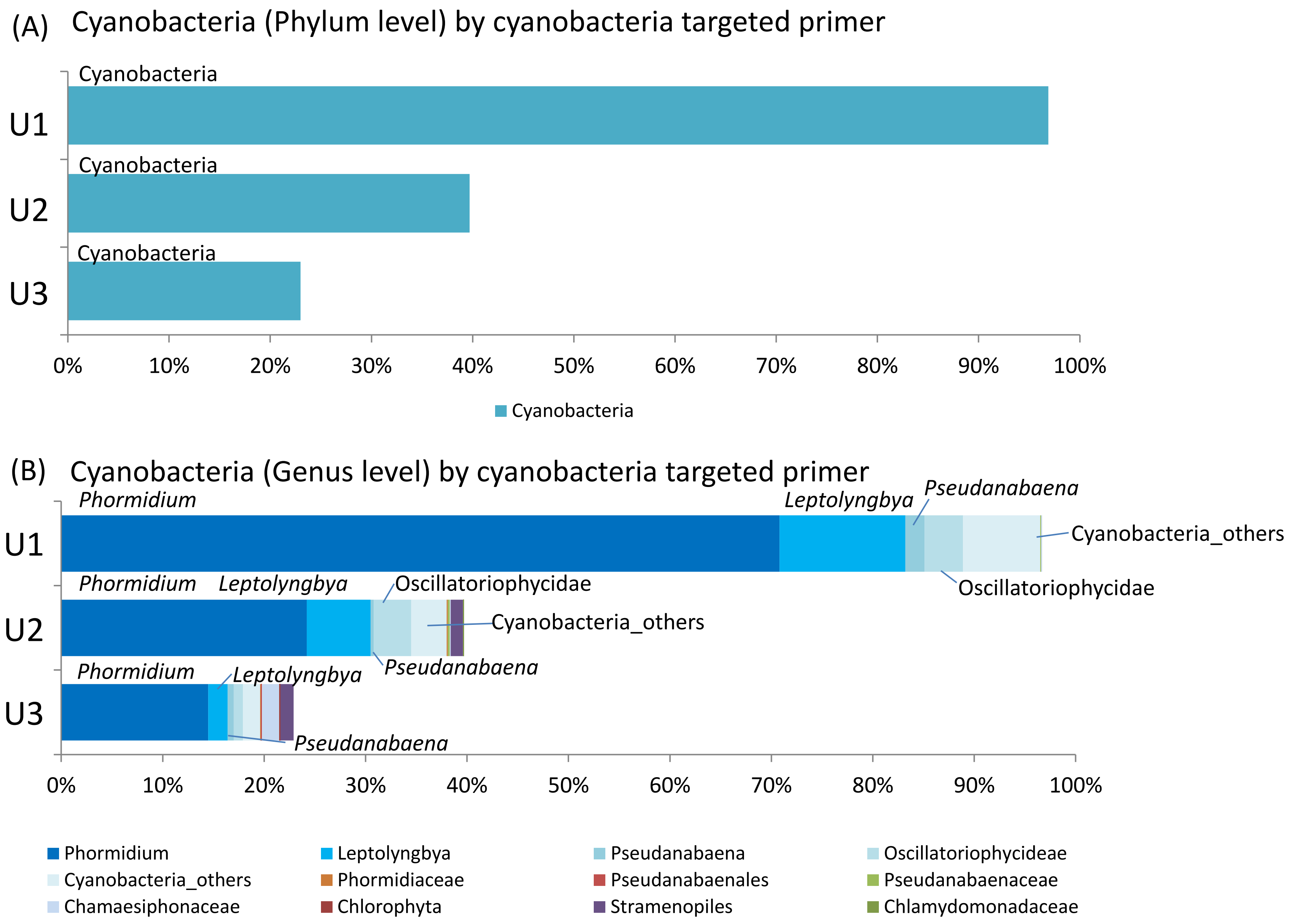

(A) Cyanobacteria (Phylum level) by cyanobacteria targeted primer
Cyanobacteria
U1
Cyanobacteria
U2
Cyanobacteria
U3
0%
10%
20%
30%
40%
50%
60%
70%
80%
90%
100%
Cyanobacteria
(B) Cyanobacteria (Genus level) by cyanobacteria targeted primer
Phormidium
Leptolyngbya
Pseudanabaena
U1
Cyanobacteria_others
Oscillatoriophycidae
Phormidium
Leptolyngbya
Oscillatoriophycidae
U2
Cyanobacteria_others
Pseudanabaena
Phormidium
Leptolyngbya
U3
Pseudanabaena
0%
10%
20%
30%
40%
50%
60%
70%
80%
90%
100%
Phormidium
Leptolyngbya
Pseudanabaena
Oscillatoriophycideae
Cyanobacteria_others
Phormidiaceae
Pseudanabaenales
Pseudanabaenaceae
Chamaesiphonaceae
Chlorophyta
Stramenopiles
Chlamydomonadaceae

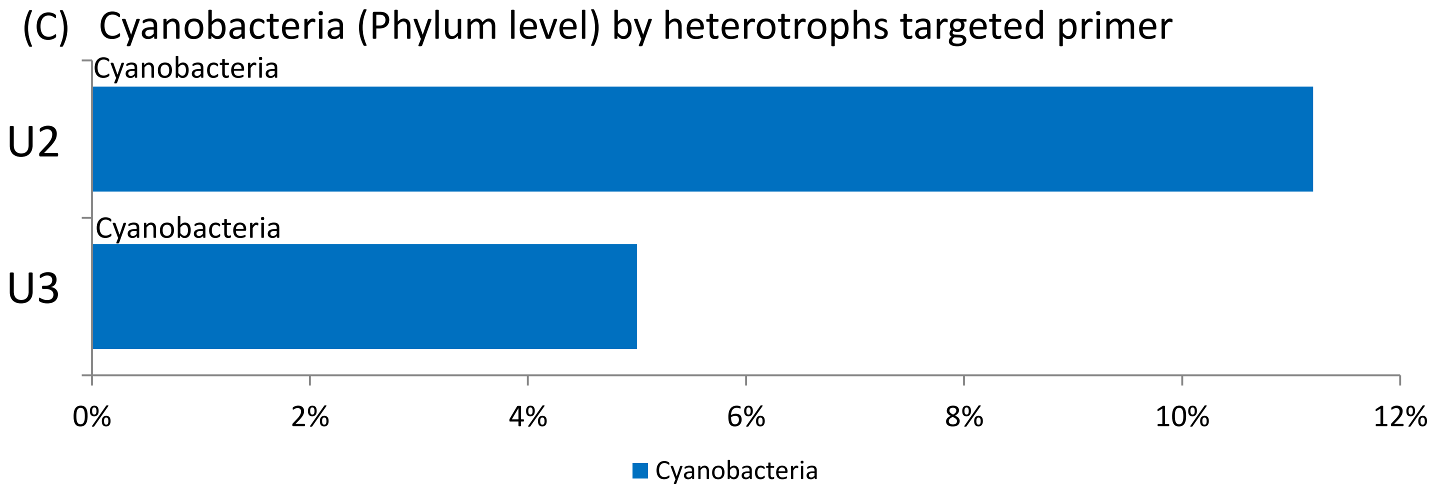

(C) Cyanobacteria (Phylum level) by heterotrophs targeted primer
Cyanobacteria
U2
Cyanobacteria
U3
0%
2%
4%
6%
8%
10%
12%
Cyanobacteria

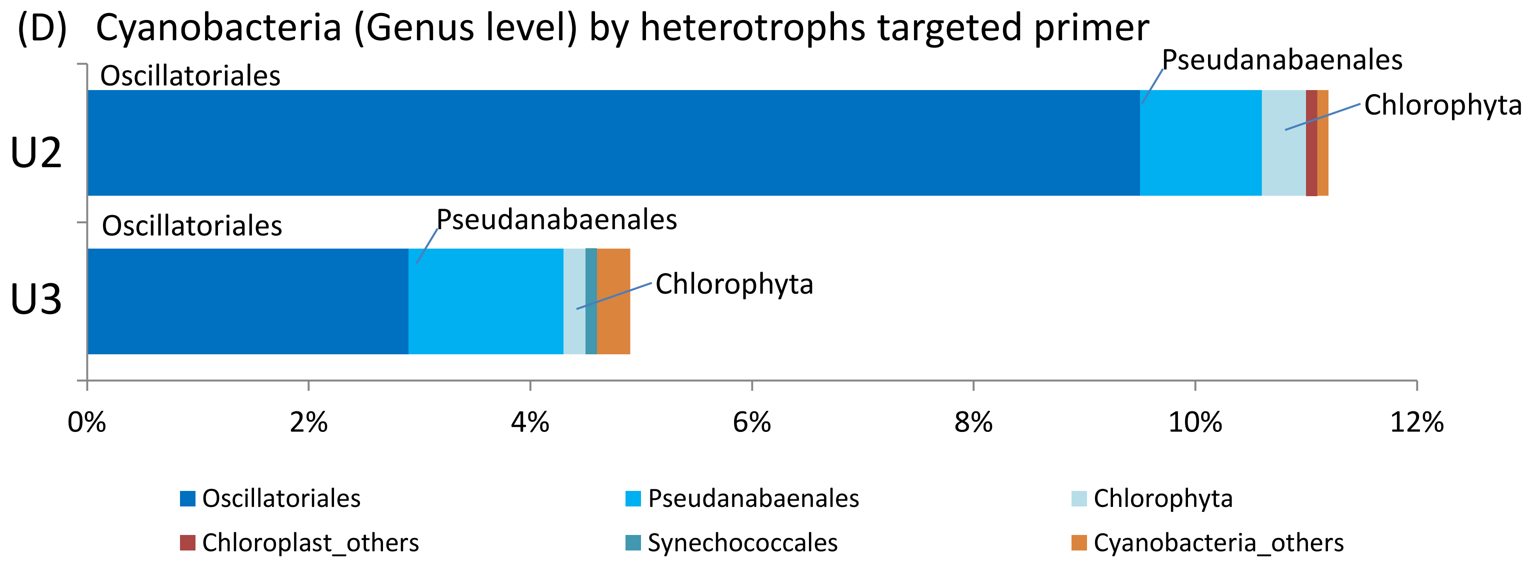

(D) Cyanobacteria (Genus level) by heterotrophs targeted primer
Oscillatoriales
Pseudanabaenales
Chlorophyta
U2
Oscillatoriales
Pseudanabaenales
Chlorophyta
U3
0%
2%
4%
6%
8%
10%
12%
Oscillatoriales
Pseudanabaenales
Chlorophyta
Chloroplast_others
Synechococcales
Cyanobacteria_others

(E) Heterotrophs (Phylum level) by cyanobacteria targeted primer

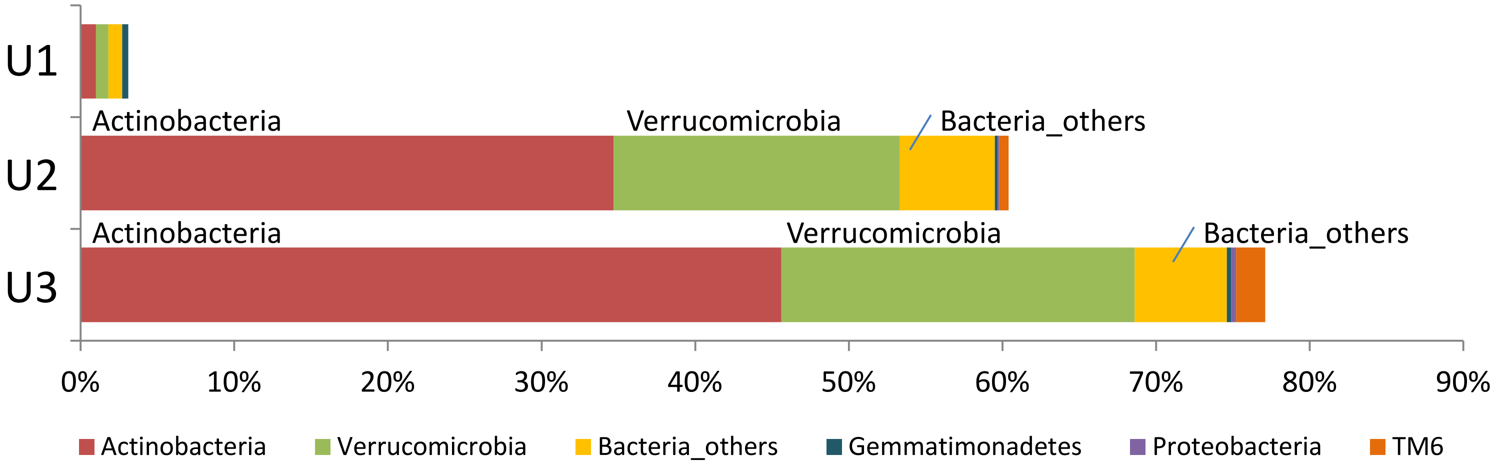

(F) Heterotrophs (Genus level) by cyanobacteria targeted primer

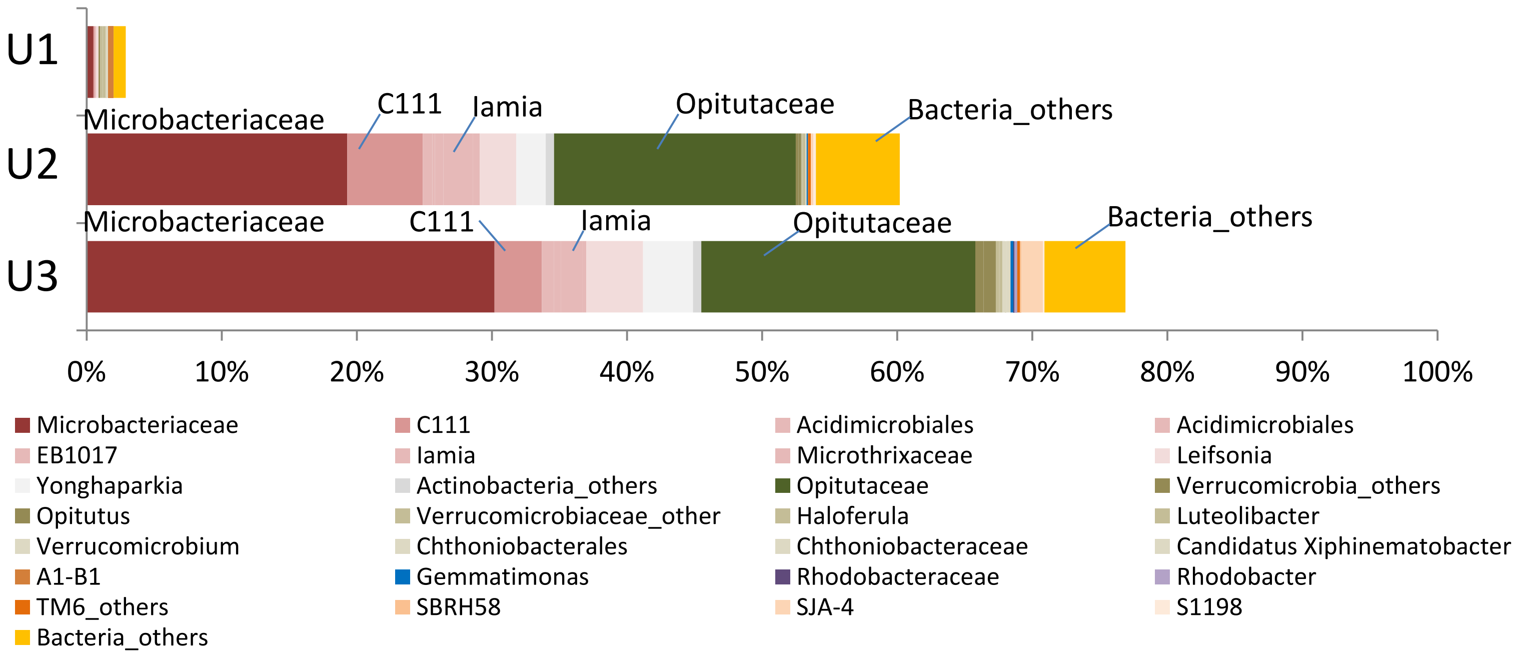

(G) Heterotrophs (Phylum level) by heterotrophs targeted primer

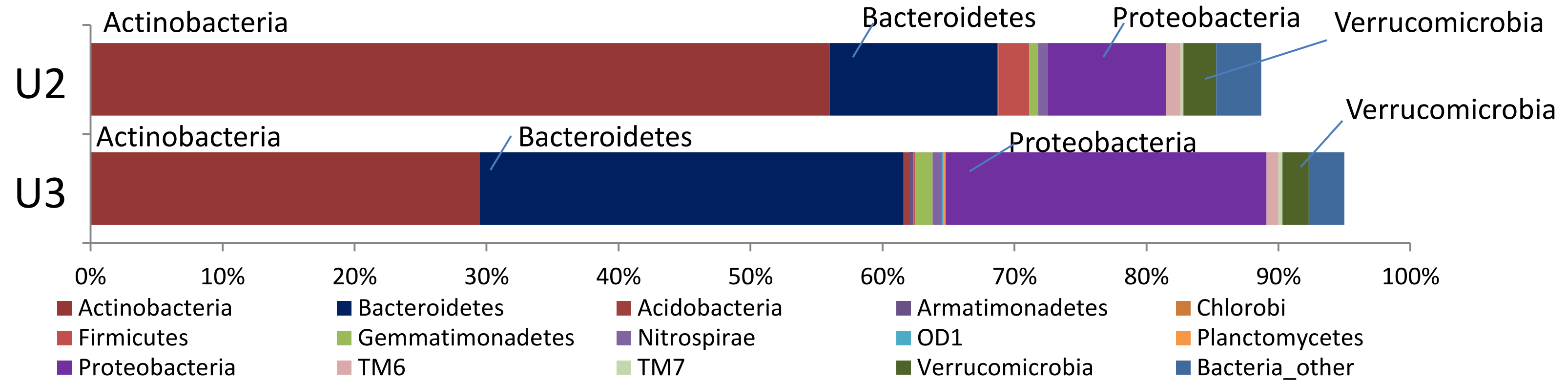

(H) Heterotrophs (Genus level) by heterotrophs targeted primer

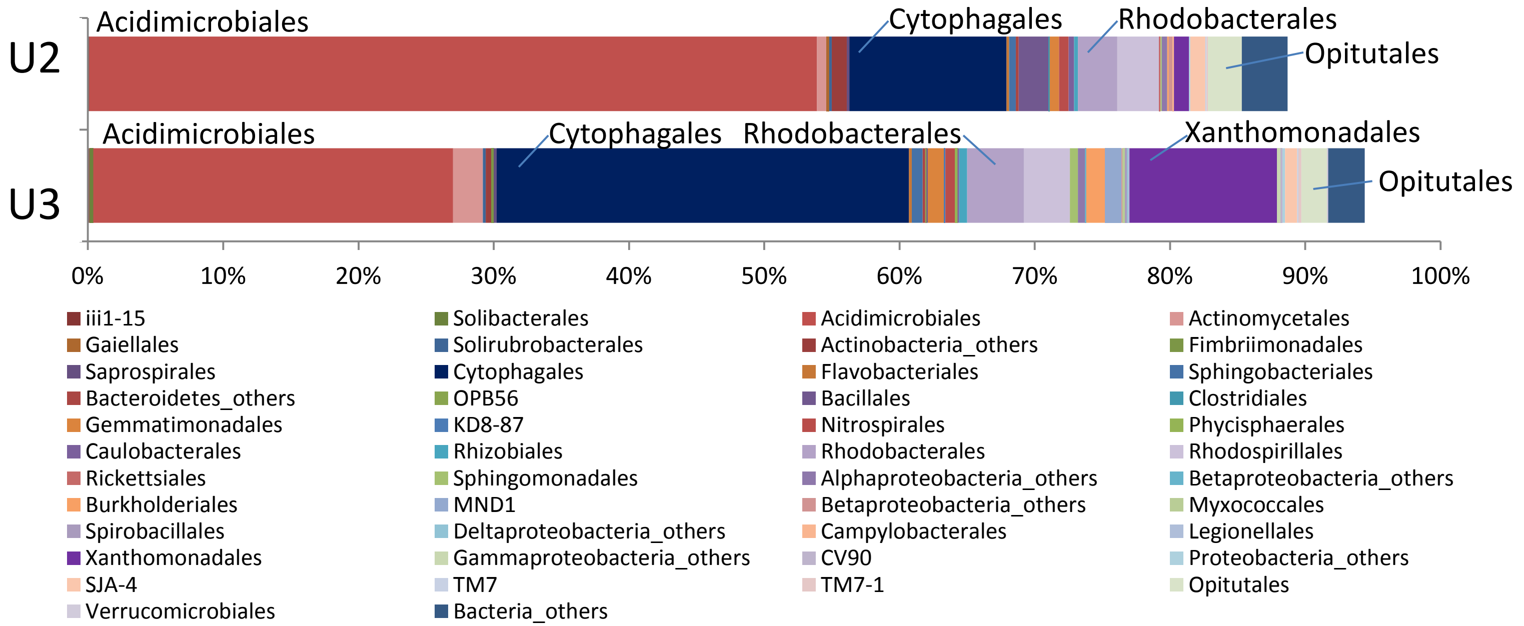

Supplementary Figure 1: Stacked column bar graphs ,(A) to (H), representing the relative abundances from individual group specific sequence data (cyanobacteria and heterotrophs) across three laminae (U1, U2 and U3) of a large conical mat from Lake Untersee, Antarctica. The relative abundances at the phylum and the genus level in cyanobacteria were shown from (A) through (D), and for heterotrophs from (E) through (H). Cyanobacteria targeted primers were used to analyze the relative abundances of cyanobacteria (A and B) and heterotrophs (E and F). The heterotroph targeted primers were used to examine the relative abundances of cyanobacteria (C and D) and heterotrophs (G and H).

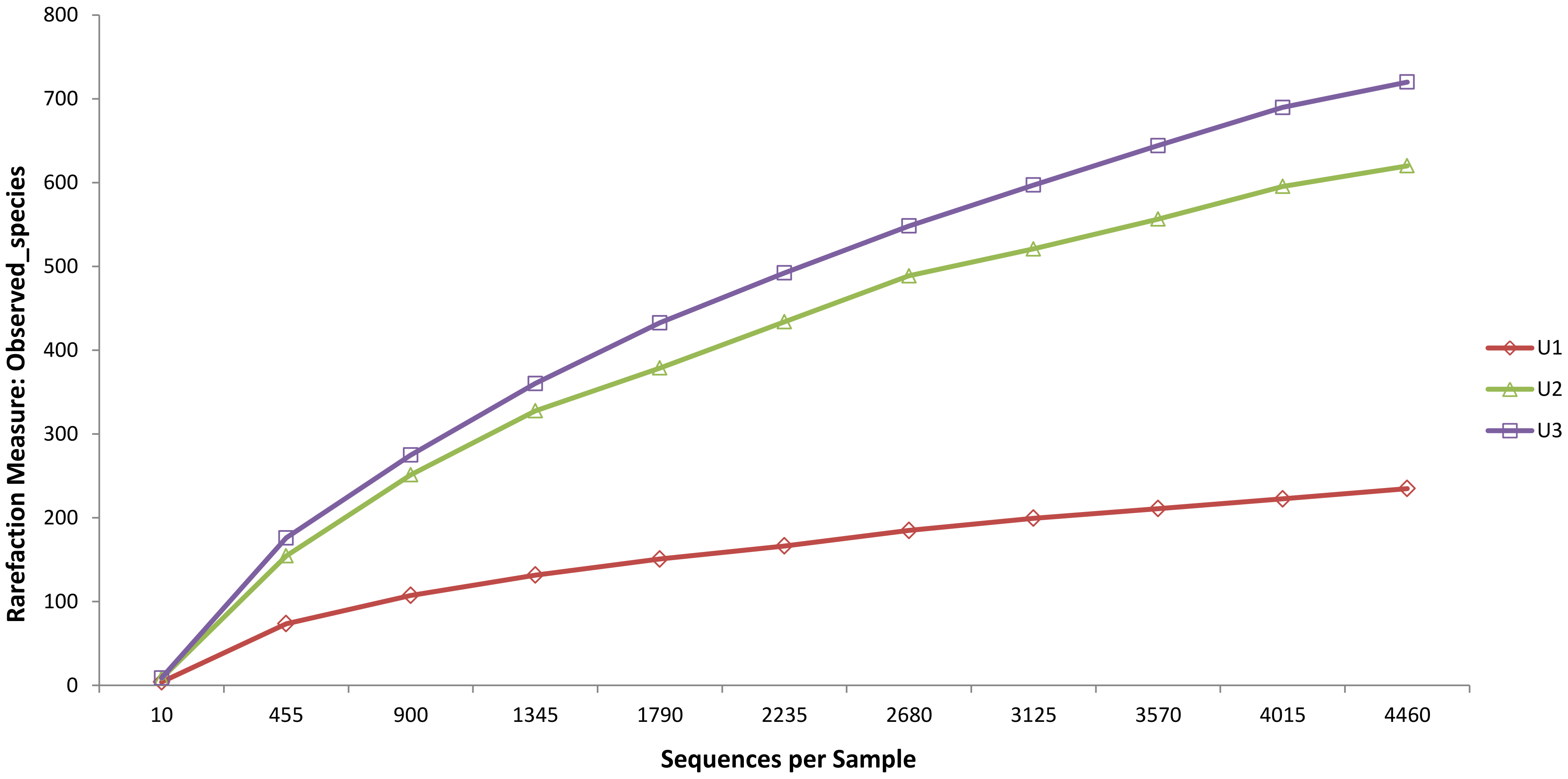

Supplementary Figure 2: **Rarefaction curves of the observed_species across three laminae of a large conical mat of Lake Untersee, Antarctica**. The observed_species at 97% sequence identity was calculated by QIIME (v1.8.0). The rarefaction curve showed that communities from all three laminae were saturated with respect to the species richness and diversity. (U1=top lamina; U2=middle lamina, and U3=inner lamina).

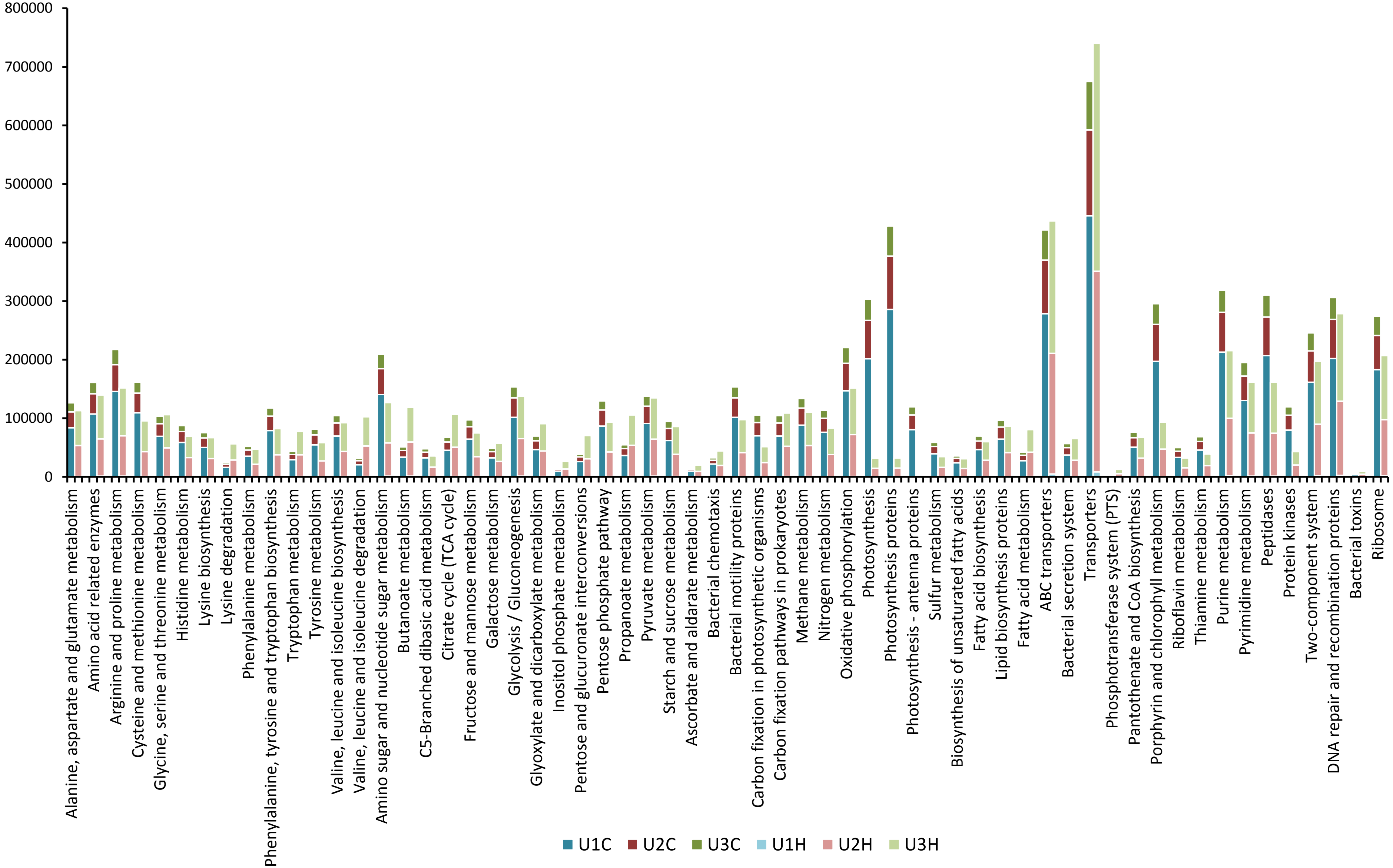

Supplementary Figure 3: Stacked column bar representing the predictive key metabolic attributes with highest resolution in U1, U2, and U3 laminae of a large conical mat from Lake Untersee, Antarctica. The cyanobacterial and the heterotrophic bacterial sequence reads were used to predict functions against the KEGG database (http://www.genome.jp/kegg/), which is implemented in PICRUSt (http://picrust.github.io/picrust/) bioinformatics software package. This supplementary figure represents graphical output of the KEGG level 3 functional attribute elaborated in Supplementary Table 4. (U1C = cyanobacterial sequence reads from the U1 lamina; U2C = cyanobacterial sequence reads from the U2 lamina; U3C = cyanobacterial sequence reads from the U3 lamina; U1H = heterotrophic bacterial sequence reads from the U1; U2H = heterotrophic bacterial sequence reads from the U2; U3H = heterotrophic bacterial sequence reads from the U3)
